# Investigating Mesozoic Climate Trends and Sensitivities With a Large Ensemble of Climate Model Simulations

**DOI:** 10.1029/2020PA004134

**Published:** 2021-06-05

**Authors:** Jan Landwehrs, Georg Feulner, Stefan Petri, Benjamin Sames, Michael Wagreich

**Affiliations:** ^1^ Department of Geology University of Vienna Vienna Austria; ^2^ Earth System Analysis Potsdam Institute for Climate Impact Research Member of the Leibniz Association Potsdam Germany

**Keywords:** paleoclimate, climate modeling, Pangaea, Triassic, Jurassic, Cretaceous

## Abstract

The Mesozoic era (∼252 to 66 million years ago) was a key interval in Earth's evolution toward its modern state, witnessing the breakup of the supercontinent Pangaea and significant biotic innovations like the early evolution of mammals. Plate tectonic dynamics drove a fundamental climatic transition from the early Mesozoic supercontinent toward the Late Cretaceous fragmented continental configuration. Here, key aspects of Mesozoic long‐term environmental changes are assessed in a climate model ensemble framework. We analyze so far the most extended ensemble of equilibrium climate states simulated for evolving Mesozoic boundary conditions covering the period from 255 to 60 Ma in 5 Myr timesteps. Global mean temperatures are generally found to be elevated above the present and exhibit a baseline warming trend driven by rising sea levels and increasing solar luminosity. Warm (Triassic and mid‐Cretaceous) and cool (Jurassic and end‐Cretaceous) anomalies result from pCO_2_ changes indicated by different reconstructions. Seasonal and zonal temperature contrasts as well as continental aridity show an overall decrease from the Late Triassic‐Early Jurassic to the Late Cretaceous. Meridional temperature gradients are reduced at higher global temperatures and less land area in the high latitudes. With systematic sensitivity experiments, the influence of paleogeography, sea level, vegetation patterns, pCO_2_, solar luminosity, and orbital configuration on these trends is investigated. For example, long‐term seasonality trends are driven by paleogeography, but orbital cycles could have had similar‐scale effects on shorter timescales. Global mean temperatures, continental humidity, and meridional temperature gradients are, however, also strongly affected by pCO_2_.

## Key Points

- We assess global long‐term climate trends through the Mesozoic era with an ensemble of climate model simulations
- Varying carbon dioxide levels cause anomalies around an overall warming trend due to changing paleogeography and increasing insolation
- Seasonal and zonal temperature contrasts as well as aridity decrease with time, while meridional gradients vary with paleogeography

## 1 Introduction

The Mesozoic Era, comprising the Triassic (∼252–201 Ma), Jurassic (∼201–145 Ma), and Cretaceous (∼145–66 Ma) periods (Cohen et al., 2013), marked a transition from the last supercontinent Pangaea toward today's fragmented continental configuration and from “old” (Paleozoic) to “new” (Cenozoic) biota that are now integral parts of the modern world (e.g., mammals, birds, angiosperms, scleractinian corals, and calcareous plankton like coccolithophores) (Stanley & Luczaj, 2015). It is furthermore characterized by some of the most incisive events in Earth history, including Large Igneous Province (LIP) volcanism, Ocean Anoxic Events (OAE) and mass extinctions, bracketing, for example, the rise and fall of the dinosaurs (Clapham & Renne, 2019; Takashima et al., 2006).

Tectonic dynamics (Golonka, 2007; Müller et al., 2016; Scotese & Wright, 2018) profoundly influenced global climatic conditions throughout the Mesozoic (Donnadieu et al., 2006a, 2009), for example, by the aggregation of all continents into a contiguous Pangaean landmass which began in the Carboniferous and culminated in the Triassic with a supercontinent almost extending from pole to pole (Parrish, 1993). This unique paleogeographic constellation shaped global climate patterns during Permian, Triassic, and Early Jurassic times, with the most prominent feature purportedly being pronounced monsoonal circulation patterns, also termed “megamonsoon” (Kutzbach & Gallimore, 1989; Parrish, 1993; Wang et al., 2014). The symmetric arrangement of huge landmasses, further enlarged by low sea levels, around the warm equatorial Tethys Sea provided optimum conditions for this phenomenon and contributed to an overall strongly seasonal climate (Parrish, 1993).

**Figure 1:**
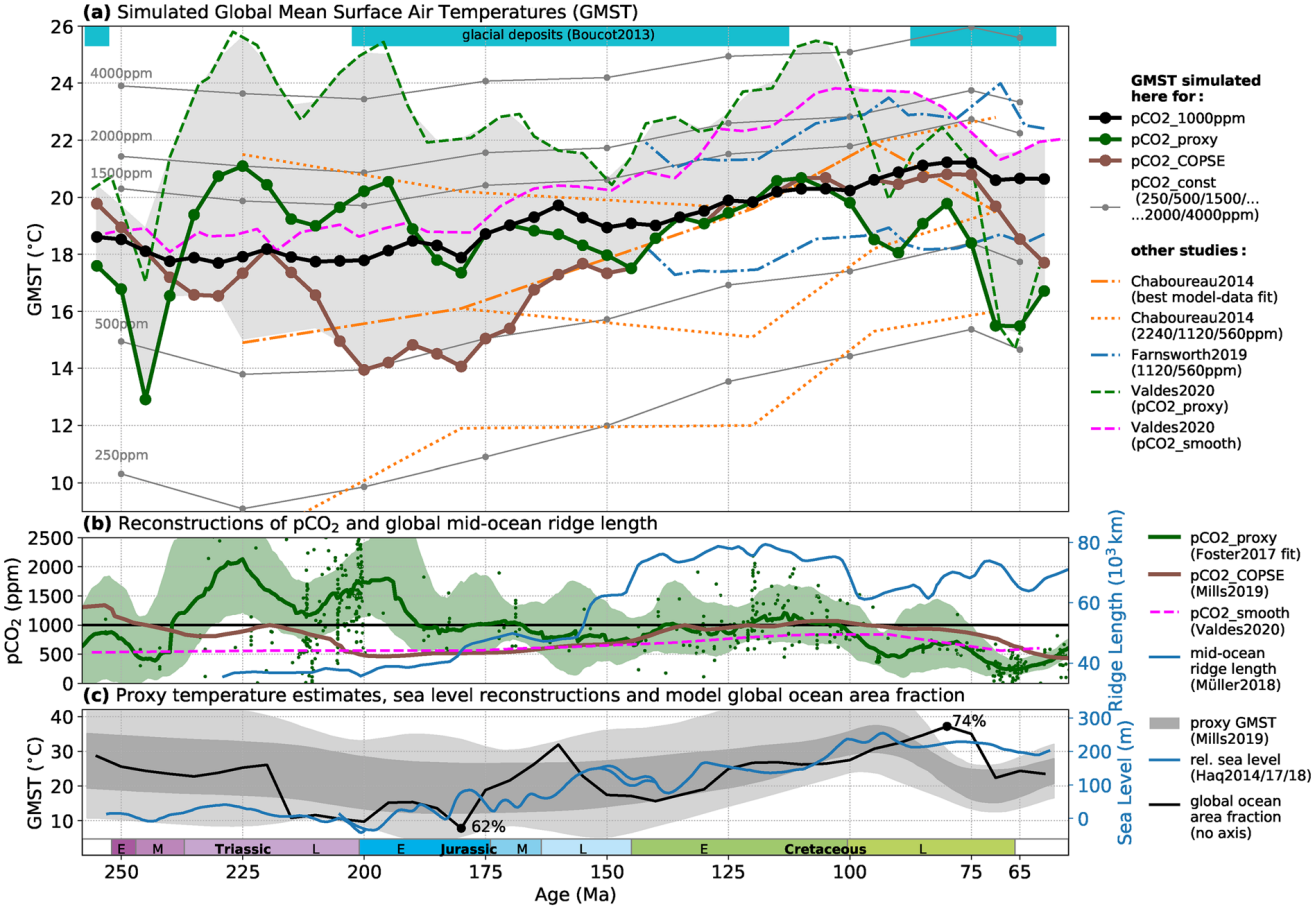
Simulated Mesozoic long‐term global mean temperature evolution (a) for different pCO_2_ pathways (b), compared with other relevant proxy and model data (a–c). (a) Each dot represents one simulated equilibrium climate state. Solid lines connect states belonging to the same pCO_2_ pathway. Green and brown dots correspond to the pathways using the proxy‐ and model‐based reconstructions *pCO*_2__*proxy* (Foster et al., 2017) and *pCO*_2__*COPSE* (Mills et al., 2019) indicated in the same colors in (b). Black and gray dots and lines correspond to the pathways with constant pCO_2_ of 1,000 ppm (black) or 250–4,000 ppm (gray). The gray‐shaded area indicates the envelope of temperatures simulated here (for *pCO*_2__*proxy* and *pCO*_2__*COPSE*) and in other climate modeling studies (broken lines, Chaboureau et al., 2014; Valdes et al., 2020). Also shown are results from Farnsworth, Lunt, O'Brien, et al. (2019). Cyan‐colored bars indicate the occurrence of glacial deposits (Boucot et al., 2013; Cao et al., 2018). (b) *pCO*_2__*proxy* (green) and *pCO*_2__*COPSE* (brown) reconstructions. For *pCO*_2__*proxy*, small dots indicate the raw data points while the line and the shading indicate the LOESS fit provided by Foster et al. (2017) and its 95% confidence intervals. The dashed pink line is the *pCO*_2__*smooth* reconstruction used by Valdes et al. (2020), which yields the pink dashed temperature curve in (a). Mid‐ocean ridge length reconstruction (blue line, Müller & Dutkiewicz, 2018). (c) The gray shading shows GMST envelope from Mills et al. (2019) (Figure 4b) which was obtained from δ^18^O, TEX86, and Mg/Ca low‐latitude sea‐water temperature data through scaling relations (see supporting information section 6 for further discussion). The black line indicates the global ocean area fraction exhibited by the paleogeographies implemented in the simulations based on Scotese and Wright (2018). The blue line represents the long term sea‐level reconstruction from Haq (2014) and Haq (2017, 2018).

Rifting of the supercontinent began during the Triassic, but the major breakup took place in the Jurassic with the opening of the North Atlantic and continued into the Cretaceous (Holz, 2015). In the course of this fragmentation, the length of global mid‐ocean ridges doubled from the Triassic into the Early Cretaceous (Müller & Dutkiewicz, 2018), which contributed to a pronounced sea‐level rise (Haq, 2014, 2017, 2018; Haq et al., 1988; Miller et al., 2005; Müller et al., 2008) and high tectonic carbon degassing rates (Brune et al., 2017; Müller & Dutkiewicz, 2018; Wong et al., 2019) (see Figures 1b and 1c). The opening of new seaways between the fragmenting continents and flooding of almost one third of the continental area increased the marine influence in many places, leading to more temperate and humid conditions with reduced seasonality (Chaboureau et al., 2014; Donnadieu et al., 2006a). Consequently, also the megamonsoon broke down during the Jurassic and more zonal climates were established (Parrish, 1993).

The Mesozoic is generally thought of as a prolonged greenhouse climate period between the Late Paleozoic and the current Cenozoic ice ages, characterized by an absence of major glaciations (Hallam, 1985; Frakes et al., 1992; Holz, 2015; Price, 2009). Evidence for continental ice is sparse and mainly occurs during Jurassic to Early Cretaceous times (Price, 1999), but the potential for ice sheet formation is still being investigated (e.g., Ladant & Donnadieu, 2016). Three major phases in the global temperature evolution have been traditionally proposed: A warm greenhouse climate from the Triassic into the Jurassic, slightly cooler conditions in late Jurassic and early Cretaceous, followed by a pronounced mid to late Cretaceous warm greenhouse and cooling into the Paleogene (Frakes et al., 1992). Studying past warm climate states is not only important to improve our understanding of Earth's history, but also becomes increasingly relevant in the light of anthropogenic global warming.

The most frequently considered proxy for Mesozoic temperature trends are δ^18^O measurements on marine fossils (Song et al., 2019; Veizer et al., 2000; Veizer & Prokoph, 2015), which yield positive temperature anomalies during the Triassic and especially the mid‐Cretaceous Cenomanian to Turonian periods (∼100–90 Ma) (Farnsworth, Lunt, O'Brien, et al., 2019; Friedrich et al., 2012). Trends of the atmospheric CO_2_ concentration, considered a major driver of Phanerozoic climate, have been reconstructed from both proxies (Foster et al., 2017; Royer, 2014) and biogeochemistry box models (Berner, 2006; Mills et al., 2019) (see Figure 1b). Compilations of lithologic climate indicators, including coals and evaporites, have been used to infer climatic zonation at different stages of the Mesozoic (Boucot et al., 2013; Cao et al., 2018; Chumakov, 2004; Scotese et al., 2014; Ziegler et al., 2003).

A number of modeling studies provide insights into global climatic conditions during certain Triassic (e.g., Huynh & Poulsen, 2005; Winguth et al., 2015), Jurassic (e.g., Sellwood & Valdes, 2008), and especially Cretaceous (e.g., Donnadieu et al., 2006a; Fluteau et al., 2007; Laugié et al., 2020; Tabor et al., 2016; Zhou et al., 2012, and references therein) time intervals or events. However, only few climate model‐based studies have been conducted that can represent long‐term climatic changes during the Mesozoic in a continuous and consistent framework. These include Donnadieu et al. (2006a) and Donnadieu et al. (2009), who performed coupled climate‐biogeochemistry‐vegetation simulations for seven Mesozoic timeslices, and found, among other things, that the fragmentation of Pangaea induced increasingly humid conditions with higher weathering CO_2_ drawdown, which alone would have caused an overall cooling through the Mesozoic. Chaboureau et al. (2014) applied similar atmosphere‐ocean and vegetation model components to five Mesozoic timeslices and assessed the match to various proxy data for three different pCO_2_ values, respectively. The authors find an expansion of temperate continental areas at the expense of arid belts and argue that this potentially fostered the diversification and expansion of angiosperms in the Cretaceous. Lunt et al. (2016) and Farnsworth, Lunt, O'Brien, et al. (2019) investigate recent ensembles of comprehensive atmosphere‐ocean‐vegetation model simulations for each Cretaceous to Eocene geological stage (∼145–34 Ma). These authors conclude that global mean temperatures within this time frame are determined by pCO_2_, increasing solar luminosity and paleogeography, mainly by the changing ocean area, as well as ocean circulation modes and feedbacks like the water vapor feedback. Based on these simulations, Farnsworth, Lunt, Robinson, et al. (2019) suggest the existence of an East Asian monsoon system since the Early Cretaceous. Very recently, a large data set of simulations with the same model for the whole Phanerozoic (Valdes et al., 2020), has become available, but a specific analysis with respect to Mesozoic climate has not yet been published. Previously, Sellwood and Valdes (2006) had discussed atmosphere‐sea ice model simulations with a similar model for one Triassic, Jurassic, and Late Cretaceous timeslice, respectively, with regard to available climate proxy distributions.

Here, the CLIMBER‐3α climate model (Montoya et al., 2005) is employed to explore stepwise long‐term climatic trends in the Mesozoic, using a recent set of paleogeographic reconstructions (Scotese & Wright, 2018). For this, equilibrium climate states for 40 geologic timeslices from 255 to 60 Ma were simulated. By systematically varying paleogeography, sea level, vegetation patterns, solar luminosity, the orbital configuration, and pCO_2_ levels (considering different recent pCO_2_ reconstructions for the Mesozoic: Foster et al., 2017; Mills et al., 2019), impacts of these respective boundary conditions are quantified. This represents the so far most extended ensemble of climate states simulated for the Mesozoic and provides the opportunity to investigate, among other things, the climatic transition from the Triassic supercontinent regime to the hot mid‐Cretaceous in a continuous and consistent framework. This study investigates trends of global mean temperatures, seasonal, meridional, and zonal temperature contrasts as well as the continental aridity. Many previous concepts of Mesozoic climates are supported and can be consolidated in this framework. Other researchers are invited to use the model output data (Landwehrs et al., 2021) for further investigations.

## 2 Methods

### 2.1 Model Description

All simulations were performed with the CLIMBER‐3α Earth System Model of Intermediate Complexity (EMIC) (Montoya et al., 2005), consisting of a modified version of the ocean general circulation model MOM3 (Hofmann & Morales Maqueda, 2006; Pacanowski & Griffies, 2000), run at a horizontal resolution of 3.75° × 3.75° with 24 vertical levels, a dynamic/thermodynamic sea‐ice model (Fichefet & Maqueda, 1997), and a fast statistical‐dynamical atmosphere model (Petoukhov et al., 2000) with a coarse spatial resolution of 22.5° in longitude and 7.5° in latitude. The main difference between CLIMBER‐3α and a coupled atmosphere‐ocean general circulation model (AOGCM) is the simplified atmosphere model which describes the dynamics of large‐scale circulation patterns, but statistically parametrizes the effects of synoptic‐scale systems rather than solving the fundamental equations. This makes the model computationally efficient and allows running large ensembles of simulations. Although CLIMBER‐3α has evolved over the years, differences between simulated and observed climatic variables under present‐day boundary conditions are still broadly similar to the original version described in Montoya et al. (2005). Specifically, surface air temperatures are overestimated in mountain regions like the Andes or the Tibetan plateau. Furthermore, simulated temperatures are generally too high over present‐day Antarctica, in particular during Southern winter, and too low over the Northern Atlantic during Northern winter. The model has been successfully used in a number of paleoclimate studies (Brugger et al., 2017, 2019; Feulner, 2017; Landwehrs et al., 2020), and compares well with other models in model intercomparison projects (Eby et al., 2013; Zickfeld et al., 2013).

### 2.2 Boundary Conditions

Climate simulations were conducted for a set of 40 timeslices with a 5 Myr spacing from 255 to 60 Ma (denoted as *T*_*5Myr*_), using paleotopographies/‐bathymetries based on the reconstruction of Scotese and Wright (2018) (also see Figure S1). These were adjusted regarding model requirements, for example, by avoiding narrow ocean channels and isolated ocean cells on the model grid resolution (see supporting information section 1). Vegetation cover was prescribed for each timeslice based on the proxy‐based estimates of climatic zonation by Boucot et al. (2013) and Scotese (2016) (also see Figure S1). Their five climate types were represented by certain fractions of tree, grass/shrub, and bare soil cover for tropical/boreotropical (75%, 20%, and 5%), arid (15%, 35%, and 60%), warm temperate (70%, 20%, and 10%), cold temperate (65%, 15%, and 20%), and polar (10%, 25%, and 65%) climates, using Bonan et al. (2002), Poulter et al. (2015), and Pfadenhauer and Klötzli (2015) for orientation. From these spatially heterogeneous vegetation patterns (*VegHet*), homogeneous vegetation patterns with average compositions over the entire land area were defined for sensitivity experiments: *VegHom* reflects the average composition for each respective timeslice, while *VegFix* has a constant average composition (51% trees, 25% grass/shrub, and 24% bare soil) over all timeslices (see Table  S1 and Figure S3). Runoff is routed along topographic gradients, with necessary modifications, for example, where internal basins occur. The solar constant (S_0_) increases approximately linearly from 1,332.7 to 1,354.7 W/m^2^ (∼97.9% to 99.5%; from 255 to 60 Ma) according to standard solar evolution (Bahcall et al., 2001) relative to its present‐day value of 1,361 W/m^2^ (Kopp & Lean, 2011).

Different pCO_2_ pathways are tested here to account for the large uncertainties associated with existing pCO_2_ reconstructions for the Mesozoic: The *P*_*pCO2_proxy*_ pathway corresponds to the fit to compiled proxy data provided by Foster et al. (2017) (see Figure 1b). This is contrasted with *P*_*pCO2_COPSE*_, which reflects the evolution of pCO_2_ obtained with the COPSE carbon cycle model (Mills et al., 2019). This model estimate was included because it correlates reasonably with proxy‐based global temperature trends (Mills et al., 2019) and helps to illustrate the spread of different proxy‐ and model‐based pCO_2_ reconstruction approaches. For the purpose of this study, the pCO_2_ values of both reconstruction pathways were rounded to the nearest hundred at each timeslice. A fixed value of pCO_2_ = 1,000 ppm (*P*_*pCO2_1000ppm*_) appears a reasonable median for most of the Mesozoic, except the Middle and Late Jurassic and the latest Cretaceous where both considered reconstructions agree on lower values (see Figure 1b). Additional simulations were performed for a subset *T*_*25Myr*_ (250, 225, 200, 175, 150, 125, 100, 75, and 65 Ma) of the timeslices, to further test the sensitivity to different boundary conditions: *P*_*pCO2_const*_ for pCO_2_ fixed at (250, 500, 1,500, 2,000, 4,000) ppm, *P*_*S0ini*_ for a fixed S_0_ of 1,332.7 W/m^2^ as well as *P*_*VegHom*_ and *P*_*VegFix*_ for homogeneous vegetation patterns (see Table 1).

To test the systematic effect of changing sea levels on global climates, further experiments were performed for raised or lowered sea levels and subsequently modified land–sea distributions for the 200 Ma (sea‐level offset ΔSL = [+40, +200] m), 150 Ma ([−40, +40] m) and 100 Ma ([−200, −40, +40] m) timeslices. The ΔSL = ±200 m offsets were chosen to represent potential effects of the long‐term sea‐level rise from the Late Triassic to the mid‐Cretaceous that has been reconstructed by various methods (e.g., Wright et al., 2020, Figure 16). The ΔSL = ±40 m offsets were tested to represent the variability of sea levels on the million‐year scale captured by sedimentary regression‐transgression sequences throughout the Mesozoic (Haq et al., 1988; Miller et al., 2005; Ray et al., 2019; Sames et al., 2020) and because 40 m is the vertical resolution of the elevation models from Scotese and Wright (2018). In the latter, coastlines were inferred from plate‐tectonic modeling and lithofacies and should represent the average best‐guess paleogeography for the covered time interval (Scotese & Wright, 2018). Independently from the determination of the coastlines, the authors assume land‐ice where glacial deposits occur (Boucot et al., 2013), but for the Mesozoic only minor ice volumes at 140–120 Ma have been inferred by Scotese (2018). For the purpose of this study, continental ice‐sheets were thus assumed to be absent in all simulations.

In most of the simulations, Earth's orbital parameters were fixed at a configuration representing a circular orbit (eccentricity *e* = 0, precession angle *ω* = 0°) with an intermediate obliquity (*ϵ* = 23.5°). To represent effects of lower‐/upper‐end obliquities and of a strongly eccentric orbit, four additional configurations with *ϵ* = 22.0° and *ϵ* = 24.5°, *e* = 0.06, *ω* = [90, 180, 270] ° were tested for the *T*_*25Myr*_ subset of timeslices. All 221 simulation runs were initialized with the same idealized modern ocean temperature and salinity profile without sea ice and were integrated for 5,000 model years (see also supporting information section 2). All analyses are based on averages over the last 500 years. For the plots and analyses in Sections 3.2, 3.3, 3.4, 3.5, atmospheric model data were interpolated on the ocean model grid to enable investigating the influence of changing land and sea areas. Table 1 provides an overview over the described ensemble of boundary conditions and an additional table with information on all individual simulation runs is included in Table S1.

**Table 1:** Overview of the Ensemble of Boundary Conditions for the Climate Model Simulations

Pathway P	Timeslices	pCO_2_ (ppm)	S_0_ (W/m^2^)	Vegetation pattern	
Baseline (fixed pCO_2_; increasing S_0_; changing paleogeography and heterogeneous vegetation patterns)					
*P*_*pCO2_1000ppm*_	*T*_*5Myr*_	1,000	1,333–1,355	*VegHet*	
Sensitivity: pCO_2_ (two different pCO_2_ reconstructions, see Figure 1b; additional constant pCO_2_ pathways)					
*P* *_pCO2_proxy_*	*T*_*5Myr*_	Foster et al. (2017) fit to proxy data	1,333–1,355	*VegHet*	
*P* *_pCO2_COPSE_*	*T*_*5Myr*_	Mills et al. (2019) COPSE model	1,333–1,355	*VegHet*	
*P* *_pCO2_const_*	*T*_*25Myr*_	[250, 500, 1,500, 2,000, 4,000]	1,333–1,355	*VegHet*	
Sensitivity: Solar Constant (S_0_ fixed at value at 255 Ma)					
*P* *_S0ini_*	*T*_*25Myr*_	1,000	1,333	*VegHet*	
Sensitivity: Homogeneous Vegetation Patterns (avg. composition of *VegHet* for individual timeslice [→ *VegHom*] or over all timeslices [→ *VegFix*])					
*P* *_VegHom_*	*T*_*25Myr*_	1,000	1,333–1,355	*VegHom*	
*P* *_VegFix_S0ini_*	*T*_*25Myr*_	1,000	1,333	*VegFix*	
Sensitivity: Orbital Configuration					[obl., ecc., prec.]
*P* *_orb_*	*T*_*25Myr*_	1,000	1,333–1,355	*VegHet*	[22.0°, 0.00, 0°]
					[24.5°, 0.06,
					90/180/270°]
Sensitivity: Sea Level (modified elevations and land‐sea masks)					Sea‐Level Offset (m)
	200 Ma	1,000	1,339	*VegHom*	[+40, +200]
*P_SL_*	150 Ma	1,000	1,344	*VegHom*	[−40, +40]
	100 Ma	1,000	1,350	*VegHom*	[− 200, −40, +40]

*Note*. See Section 2.2 for further explanation.

## 3 Results

### 3.1 Global Mean Temperature Evolution

**Figure 2:**
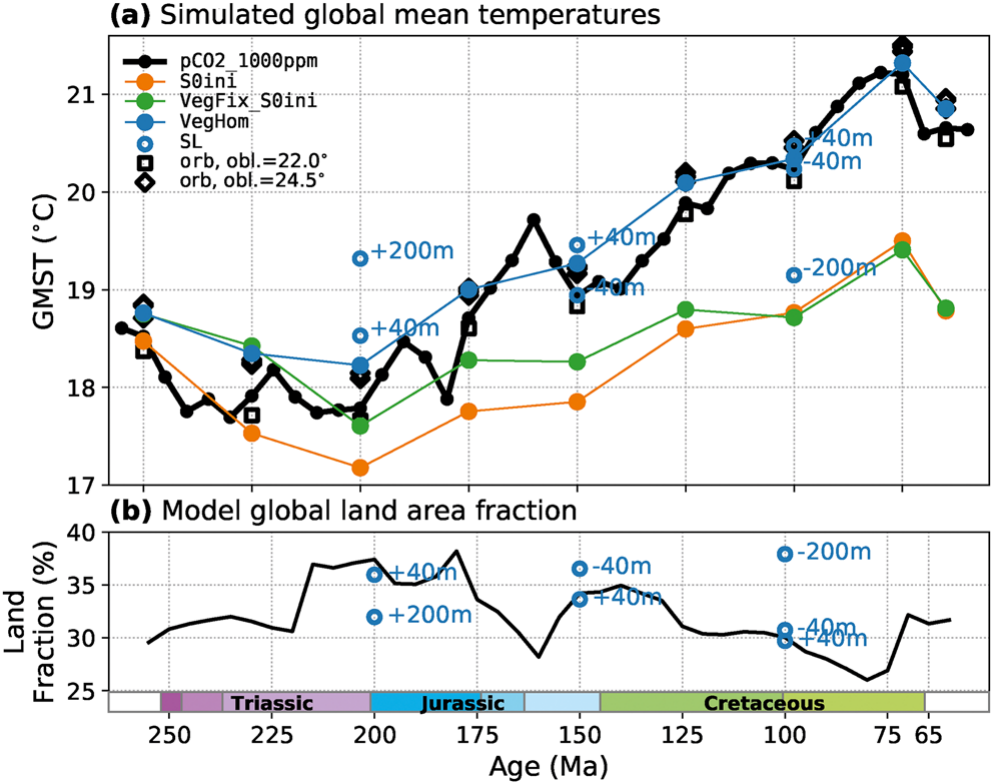
Simulated global mean surface air temperatures (GMST) in several sensitivity experiments at pCO_2_ = 1,000 ppm (a), and global land area fractions in the implemented paleogeographies (b). (a) Each dot represents one equilibrium climate simulation. Black dots and lines correspond to the *P*_*pCO2_1000ppm*_ pathway and are the same as in Figure 1a. Information on the respective sensitivity experiments is given in Section 2.2 and Table 1. (a and b) Blue circles indicate the GMST (a) and global land area fraction (b) in sensitivity experiments with modified relative sea levels.

The simulated global mean surface air temperatures (GMST) for all tested pCO_2_ pathways are summarized in Figure 1a, and for all other sensitivity experiments in Figure 2a. Overall, the GMSTs at pCO_2_ = 1,000 ppm (*P*_*pCO2_1000ppm*_, black dots and lines in Figures 1a and 2a) exhibit a slight cooling through the Triassic to a minimum of 17.7°C in the Late Triassic and then a +3.5°C warming to up to 21.2°C at 80 Ma in the Late Cretaceous. The two considered pCO_2_ reconstructions (*P*_*pCO2_proxy*_ and *P*_*pCO2_COPSE*_, Figure 1b) do, however, suggest deviations of up to ∼5°C from this median pathway (Figure 1a). To decompose these temperature trends, we further examine the *P*_*pCO2_1000ppm*_ pathway, which reflects the effects of changing paleogeography, vegetation patterns, and the increasing solar constant S_0_. The latter elevates the GMST at 65 Ma by +1.9°C (difference between *P*_*pCO2_1000ppm*_ and *P*_*S0ini*_ in Figure 2a, also see Table S1), which indicates the order of warming through the Mesozoic caused by the increasing solar luminosity if everything else would have remained the same.

Despite fixed pCO_2_ and S_0_, the *P*_*S0ini*_ temperatures exhibit variations with time (see Figure 2a) that reflect the changing distribution and extent of land masses and vegetation patterns: The GMST decreases from 18.5°C at 250 Ma to 17.2°C at 200 Ma and subsequently rises to 19.5°C at 75 Ma. These trends correlate with changes in the fraction of Earth's surface occupied by land (Figure 2b, black line), which has a higher average albedo than ocean areas and thus influences Earth's energy balance. The global land fraction in the implemented paleogeographies is highest during the Late Triassic‐Early Jurassic (up to 38%) and lowest in the Late Cretaceous (down to 26%) which is related to the change of relative sea levels (Figure 1c, blue line) that are implicitly included in the employed paleogeographic reconstructions (Scotese & Wright, 2018). The effect of raised or lowered sea levels and the resulting flooding/exposure of continental area is also demonstrated for individual timeslices (Figure 2, blue circles). For example, the global land fraction increases by +8% for a 200 m sea‐level fall at 100 Ma and the GMST is reduced by −1.2°C. In contrast, a +1.2°C global warming is obtained for a +200 m sea‐level rise at 200 Ma. These findings suggest that the lower sea level and larger continental area in the Triassic‐Jurassic would have contributed to relatively low temperatures, and that the subsequent sea‐level rise provided a major part of the ≈+2°C global warming toward the Late Cretaceous, exhibited by the *P*_*S0ini*_ pathway. To test to which extent these trends are dependent on the uncertain reconstruction of paleotopography, lapse‐rate corrected global mean temperatures at sea level were calculated (see Figure S12). On the global scale, they follow the same trend although differences can be noted, especially around 100 Ma where the global mean topography height reaches a maximum. On a regional scale, differences resulting from different paleogeographic reconstructions can potentially be much more pronounced.

The described temperature rise between 200 and 75 Ma is ∼0.5°C larger than for a fixed homogeneous vegetation composition (*P*_*VegFix_S0ini*_, Figure 2a, green line), which yields slightly elevated temperatures in the Triassic–Jurassic. This is related to the large portion of bare land area in the Pangaean low‐to mid‐latitudes (for *VegHet*), which contributes to a higher surface albedo in these regions with high insolation and thus slightly lower GMSTs in the Triassic and Jurassic than with a homogeneous vegetation (see difference between *P*_*VegHom*_ and *P*_*pCO2_1000ppm*_, Figure 2a). In our modeling framework, the trend toward denser vegetation, especially in low‐latitude land areas, thus contributes to a Late Triassic‐Late Cretaceous warming, although on a slightly smaller scale than changing sea levels and solar luminosity. On the *P*_*VegFix_S0ini*_ pathway, the distribution of land area is the only varying boundary condition if lapse‐rate corrected air temperatures at sea level are considered. We find that global mean sea‐level temperatures are indeed strongly negatively correlated with the global land fraction, but also correlate negatively with the average absolute latitude of land area which peaks around 90 Ma (see Figure S13). This indicates that paleogeographic changes contribute to the warming toward the mid‐Cretacous both by the decreasing land area and by its distribution to higher latitudes. The simulated GMSTs also vary on the order of ∼0.4°C–0.6°C with the orbital configuration, with low‐obliquity configurations (*ϵ* = 22.0°, *e* = 0) being generally cooler than those with higher obliquity and eccentricity (*ϵ* = 24.5°, *e* = 0.06) (see Figure 2a).

As already noted, the amplitudes of GMST variations resulting from the two considered pCO_2_ reconstruction pathways exceed those of the other discussed boundary conditions. The pCO_2_ proxy data and the provided fit (Foster et al., 2017) exhibit a large spread in the Triassic, yielding relatively low temperatures in the Early–Middle Triassic but very high temperatures in the Late Triassic to earliest Jurassic (up to 21.1°C at 225 Ma with 2,000 ppm). The COPSE model‐derived pCO_2_ falls to ∼500 ppm in the Early Jurassic, yielding relatively low temperatures from the latest Triassic to the Middle Jurassic (down to 13.9°C at 200 Ma with 500 ppm). Although discrepancies between both pathways are evident, common relative trends can be identified: Elevated temperatures in the Triassic which decrease into the Jurassic, but subsequently rise again toward the mid‐Cretaceous. For both reconstructions, mid‐Cretaceous pCO_2_ levels do not exceed the relatively high values around the Late Triassic, but the additional warming from the previously discussed drivers, including rising sea levels and solar luminosity, contributes to similar or higher temperatures. The *P*_*pCO2_proxy*_ pathway does not capture the prominent Cenomanian–Turonian peak (∼100–90 Ma) suggested by δ^18^O proxy data (Friedrich et al., 2012), whereas *P*_*pCO2_COPSE*_ yields sustained high temperatures above 20°C from 110 to 75 Ma and generally yields long‐term trends similar to the temperature proxies (see Figure 1c), as already noted by Mills et al. (2019). Overall, the simulated GMSTs for both reconstructions are elevated above pre‐industrial values of ∼14°C for most of the Mesozoic, and for the mid‐Cretaceous (around 110 Ma) both agree on temperatures >20°C. For the latest Cretaceous, they consistently yield a pronounced cooling.

### 3.2 Seasonal Temperature Contrasts

**Figure 3:**
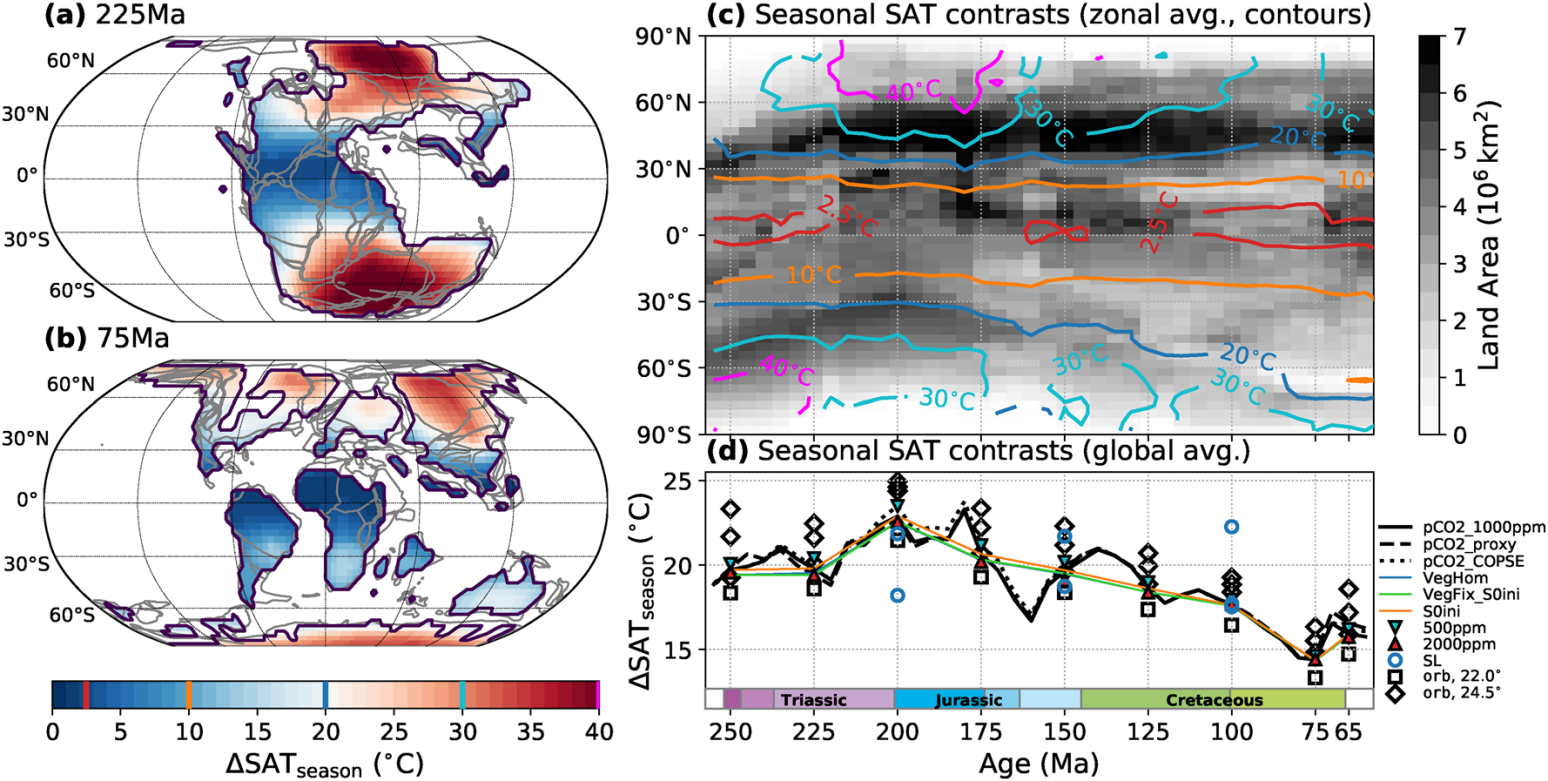
Simulated seasonality of surface air temperatures (SAT). (a and b) Seasonal SAT changes (max. difference between the DJF, MAM, JJA, and SON seasonal averages) for the 225 and 75 Ma timeslices of the *P*_*pCO2_1000ppm*_ pathway. Gray lines indicate the tectonic plate boundaries in the rotation model of Scotese (2008) (as published by Cao et al., 2018). (c) The gray shading shows the changing latitudinal land area distribution implemented in the simulations. Contours indicate the zonal mean SAT seasonality in the *P*_*pCO2_1000ppm*_ simulations, resolved by latitude and geologic time. For this, zonal mean data from all 5 Myr timeslices were aggregated with each timeslice represented by one grid column. The values associated the contour lines are also indicated in the colorbar of panel (b). (d) Globally averaged continental SAT seasonality for different boundary conditions.

It has been previously suggested, that the climate on the Pangaean supercontinent was characterized by large seasonal contrasts and intense monsoons (e.g., Kutzbach & Gallimore, 1989; Parrish, 1993), but that these conditions decayed with continental breakup through the Mesozoic (e.g., Parrish, 1993). Here, we assess the systematic impact of paleogeography and other boundary conditions on the global‐scale amplitude of seasonal temperature contrasts. For this, maximum surface air temperature (SAT) differences between the DJF, MAM, JJA, and SON seasonal averages were calculated. To illustrate the evolution of regional patterns through the Mesozoic, maps of the resulting temperature seasonality are shown for one Late Triassic (225 Ma) and one Late Cretaceous (75 Ma) timeslice of the *P*_*pCO2_1000ppm*_ pathway in Figures 3a and 3b. It can be seen that the vast mid‐ and high‐latitude continents of Pangaea experience strong seasonal temperature variations in the early Mesozoic (Figure 3a), while these contrasts are reduced in the fragmented continental configuration of the Late Cretaceous (Figure 3b).

The evolution of the zonal mean continental SAT seasonality obtained from all 40 timeslices of the *P*_*pCO2_1000ppm*_ pathway is summarized in Figure 3c (contours). The gray shading indicates the changing latitudinal distribution of land area in the paleogeographies implemented for these simulations. This illustrates the correlation of the amplitude of seasonal temperature changes with the extent of land area, especially in the mid to high latitudes. These amplitudes are highest during the Early Triassic–Early Jurassic on the Southern Hemisphere and during the Late Triassic–Early Jurassic on the Northern Hemisphere and reach up to 40°C. Toward the Late Cretaceous, the mid to high latitude seasonal temperature contrasts are considerably reduced on both hemispheres and only reach ∼20°C–30°C. These trends are also apparent in the tropical latitudes but are strongly dampened and remain below ∼10°C.

On a global scale, the average continental SAT seasonality (Figure 3d) peaks at ∼22°C in the Late Triassic–Early Jurassic and falls down to ∼15°C in the Late Cretaceous. As pCO_2_, S_0_ and vegetation patterns do not significantly affect these trends, the latter are mainly driven by paleogeographic changes and closely mirror the change of the global land area fraction (see Figure 2b). The major contribution of sea‐level rise to reduced seasonality in the Late Cretaceous relative to the Late Triassic‐Early Jurassic is demonstrated by the experiments with elevated or lowered sea level at 200 and 100 Ma: with a sea level elevated by 200 m, at 200 Ma, the global SAT seasonality is reduced to levels comparable to those at 100 Ma, and vice versa. Although these results suggest that the long‐term seasonality trends were indeed determined by sea‐level changes and continental fragmentation, we also find that different orbital configurations modify SAT seasonality to a similar degree (Figure 3d). The global SAT seasonality generally varies by about 4°C–5°C between orbital configurations which yield a lower seasonality (lower obliquity *ϵ* = 22.0°) and higher seasonality (higher obliquity *ϵ* = 24.5°). This suggests that orbital cycles would have considerably overprinted Mesozoic long‐term seasonality trends.

### 3.3 Meridional Temperature Contrasts

**Figure 4:**
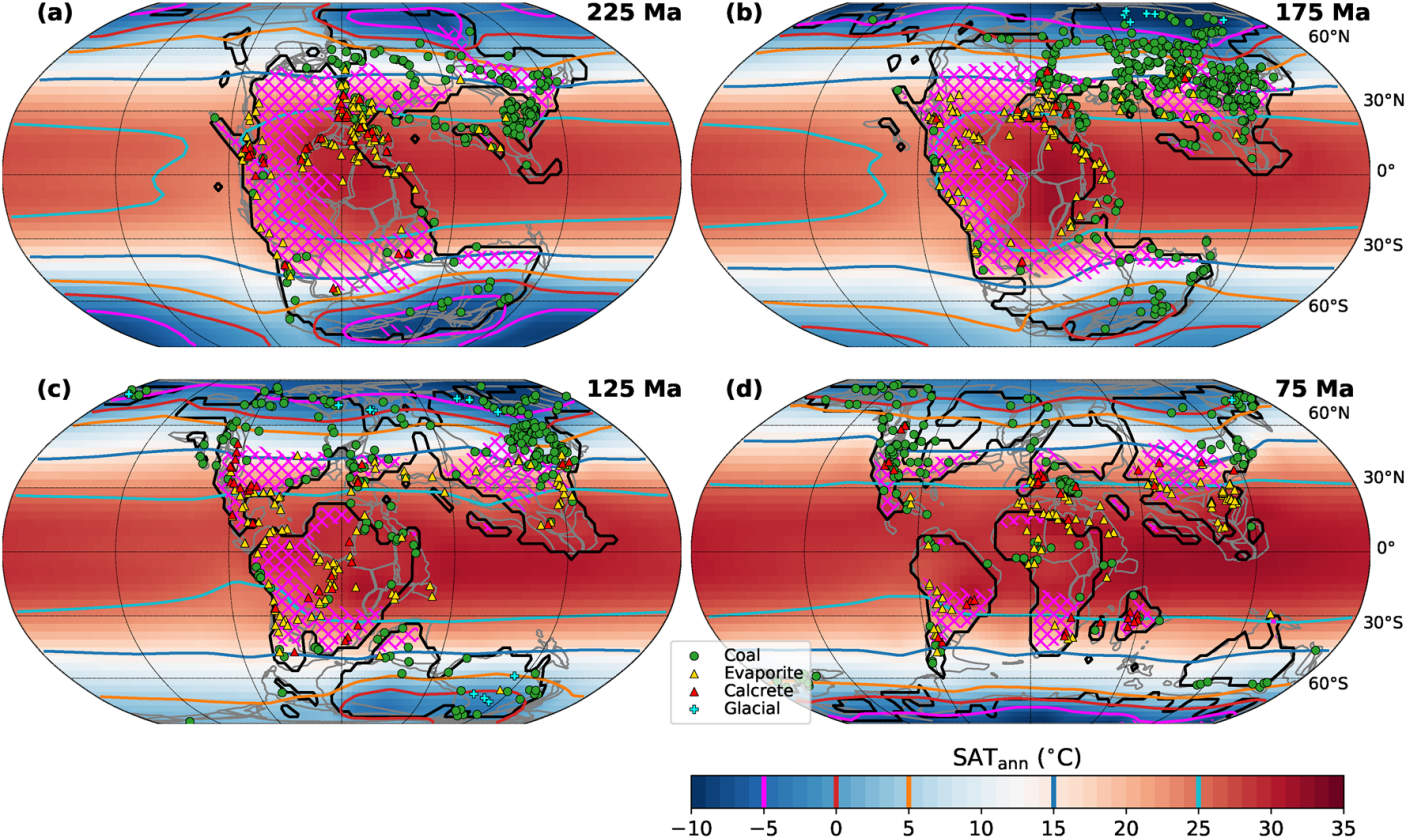
Simulated annual mean surface air temperatures (SAT_ann_, colors, and contours) and arid regions (pink hatches) for four selected Mesozoic timeslices (225, 175, 125, 75 Ma) at pCO_2_ = 1,000 ppm. Colors and contours: annual mean SAT for the *P*_*pCO2_1000ppm*_ pathway. Pink hatches: Relatively dry regions based on an aridity index <1.5 (see Section 3.5 for further explanation). “///” hatches are for 1,000 ppm and “\\\” hatches for 500 ppm. Markers: Locations of climatic indicators from Boucot et al. (2013). Coal and evaporite/calcrete occurrences are generally viewed as indicators for humid or dry conditions, respectively, and are thus qualitatively compared with the extent of simulated hatched arid regions here (see Section 3.5). In contrast to, for example, glacial deposits, they cannot be interpreted primarily as temperature proxies. Gray lines indicate the tectonic plate boundaries in the rotation model of Scotese (2008) (as published by Cao et al., 2018).

Simulated annual mean SAT for four selected timeslices at 1,000 ppm (*P*_*pCO2_1000ppm*_ pathway) are shown in Figure 4. It can be seen that for these simulations equatorial SATs mostly exceed 25°C, while temperatures below 0°C occur especially where extensive land masses exist in polar latitudes. To systematically assess the evolution of meridional temperature contrasts and their dependence on paleogeographic changes and other boundary conditions, the equator‐to‐pole thermal gradients where calculated for all conducted simulations. Here, these were defined as the difference between low‐latitude (<30° lat.) and high latitude (>60° lat.) annual and zonal mean SATs.

**Figure 5:**
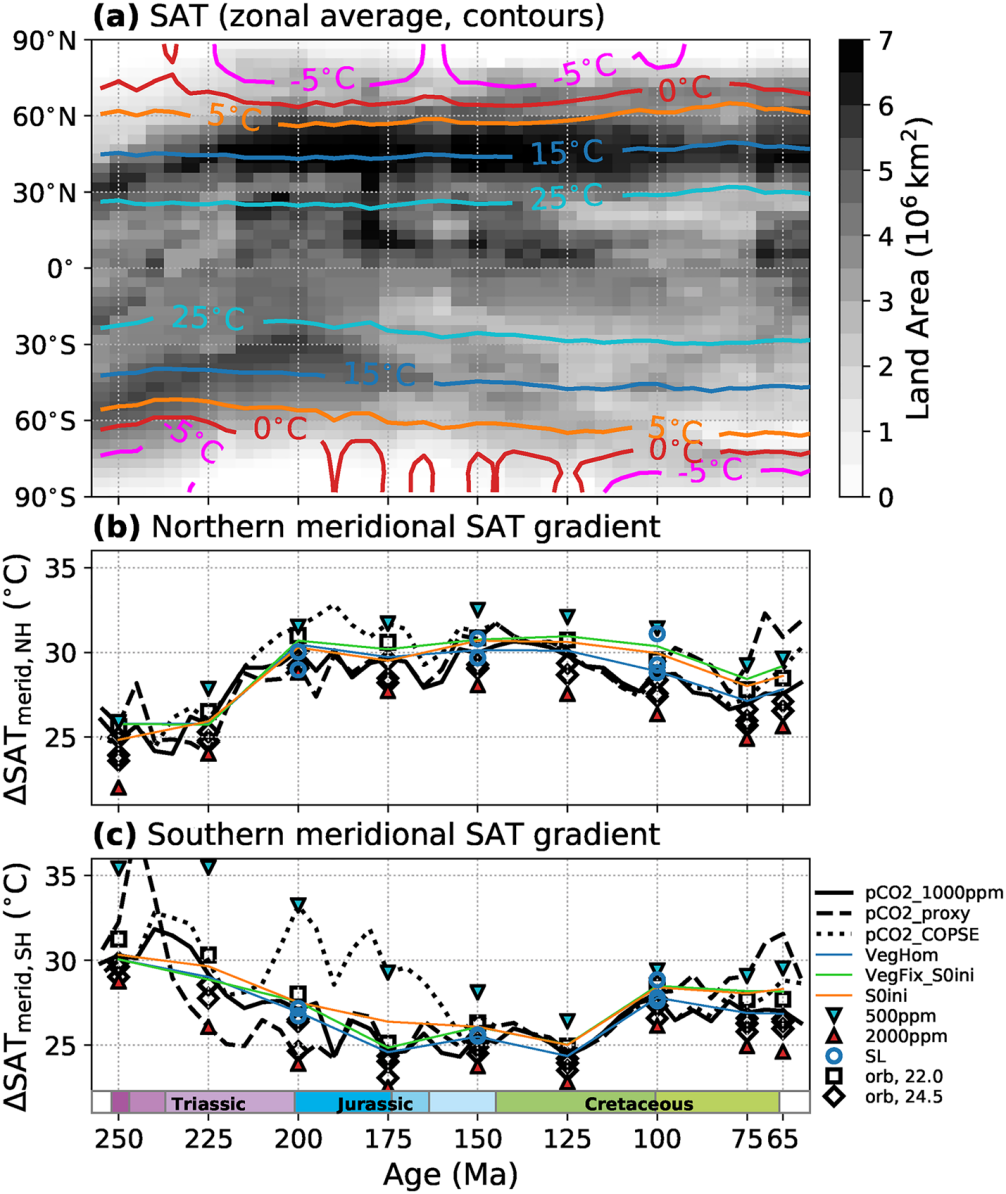
Evolution of simulated zonal mean surface air temperatures (SAT) (a), and the thermal contrast between low‐latitude and northern (b) and southern (c) high‐latitude regions. (a) Contours indicate annual zonal mean SAT for all runs of the *P*_*pCO2_1000ppm*_ pathway, resolved by latitude and geologic time. For this, zonal mean SAT data from all 5 Myr timeslices were aggregated with each timeslice represented by one grid column. The contour colors correspond to those in Figure 4. The gray shading indicates the changing latitudinal distribution of land area in the model paleogeographies. (b and c) Annual zonal mean SAT difference between low‐latitude (<30° lat.) a northern (b) and southern (c) high‐latitude regions (>60° lat.) through time for the different tested boundary conditions.

Plots of zonal mean SAT and SST profiles for all timeslices are included in the accompanying data repository (also see Figure S16). The evolution of zonal mean SATs for the *P*_*pCO2_1000ppm*_ pathway is summarized in Figure 5a (contours). On the one hand, the contours reflect the previously discussed general warming trend from the Late Triassic‐Early Jurassic to the Late Cretaceous. On the other hand, it can be inferred that low polar temperatures and high meridional temperature gradients (Figures 5b and 5c) correlate to first order with the amount of land in polar latitudes. From the end‐Permian to the Late Triassic, Pangaea shifted northwards, which leads to a high‐latitude warming and a ∼5°C lower thermal gradient (Figure 5c) in the southern hemisphere, whereas the opposite is observed in the northern hemisphere (Figure 5b). The northern hemisphere thermal gradient shows a decrease from the Late Jurassic into the Late Cretaceous in line with the general decrease of land area related to sea‐level rise. In contrast, the southern hemisphere meridional temperature gradient increases during this time due to the migration of Antarctica toward the South Pole. Despite the primary control of paleogeography on these trends, our experiments suggest considerable effects of other boundary conditions. For example, the northern hemisphere thermal gradients generally decrease (on the order of ∼5°C) in response to an increase of pCO_2_ from 500 to 2,000 ppm and the associated warming. This effect can be related to the snow and sea‐ice albedo feedback and is even more pronounced in the southern hemisphere especially before the Middle Jurassic. Relatively low pCO_2_ levels suggested by the tested reconstructions for the Jurassic and latest Cretaceous could thus have contributed to higher equator‐to‐pole thermal gradients relative to the mid‐Cretaceous. Through the ice albedo feedback, the warming associated with the increased solar luminosity also contributes to lower thermal gradients in the Late Cretaceous on a smaller, but notable scale.

Periods with lower northern and southern temperature gradients are also found to be times with enhanced meridional heat transport by the ocean in the respective hemisphere (see supporting information section 8 and Figure S18). This also appears to be related to enhanced meridional overturning (see also Figure S19) and ocean mixing (Figures  S20 and S21) and deep water formation. These are enhanced at high northern latitudes in the early Triassic as well as again in the mid‐ and late Cretaceous, and at high southern latitudes from the Early Triassic to the mid‐Cretaceous. However, it will require further work to disentangle these phenomena and evaluate their significance. Ocean model output data are included in the accompanying data repository.

### 3.4 Zonal Temperature Contrasts

Our simulations suggest the existence of considerable zonal climatic contrasts prior to the breakup of Pangaea, as can be inferred from the simulated SAT patterns and the distribution of arid regions (pink hatches, see Section 3.5 for explanation) in Figures 4a and 4b: High temperatures and humid conditions are inferred for the tropical latitudes of eastern Pangaea, in contrast to its western low‐ to mid‐latitudes, where temperatures are generally lower and arid regions are more extended. These differences are related to circulation patterns in the Panthalassa ocean where an anticyclonic subtropical gyre in each hemisphere transports warm waters along the equator into the Tethys, but colder water from higher latitudes to the western Pangaean tropics and subtropics. These provide less moisture and precipitation and lower temperatures in contrast to the very warm Tethys Sea. However, the opposite effect occurs in the mid‐ to high latitudes where cyclonic subpolar gyres warm the western coasts compared to the eastern ones.

**Figure 6:**
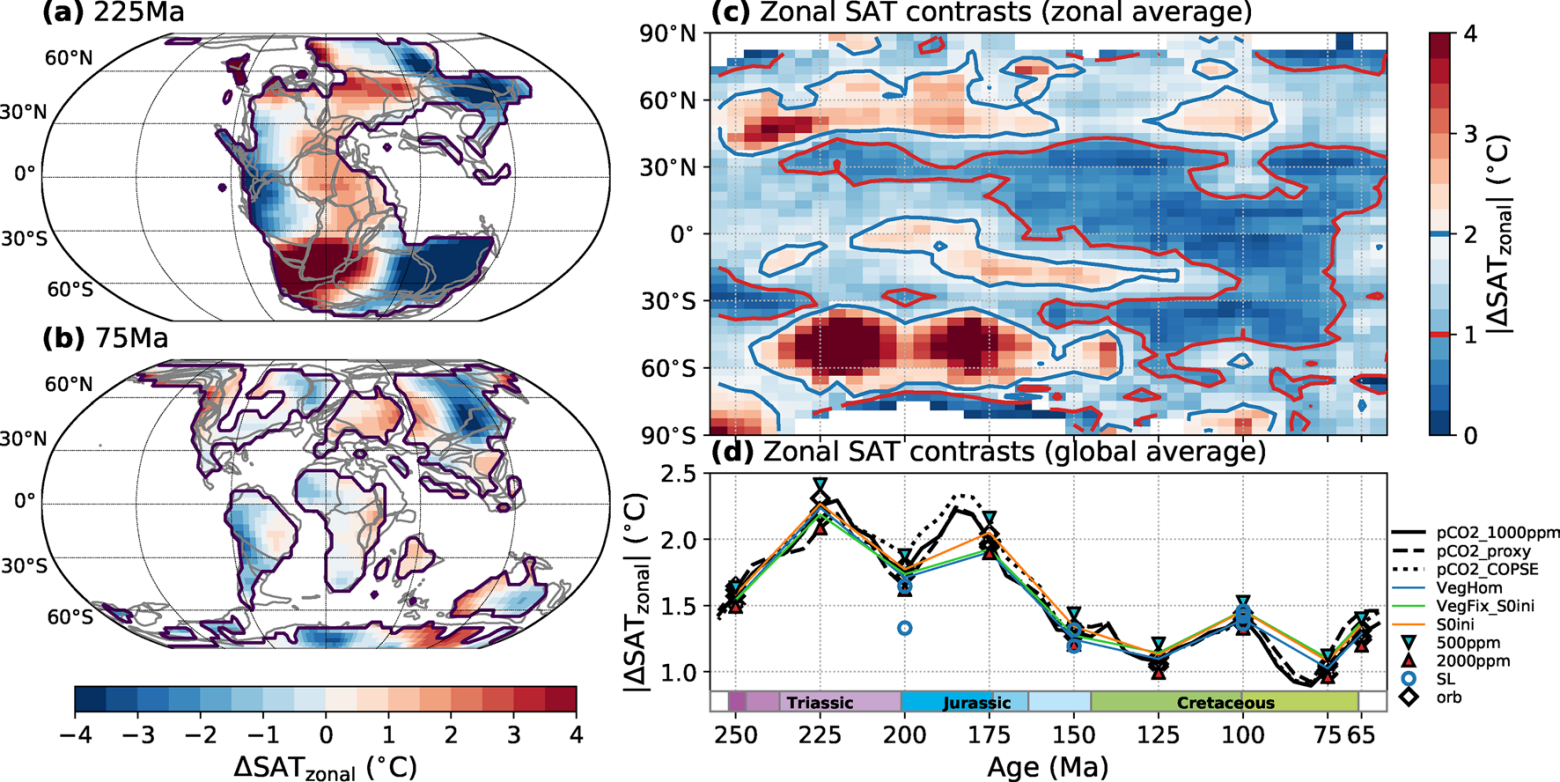
Zonal contrasts in simulated continental surface air temperature patterns (SAT) through the Mesozoic. (a and b) Deviation of continental annual mean SATs from their zonal mean values for two timeslices of the *P*_*pCO2_1000ppm*_ pathway. Gray lines indicate the tectonic plate boundaries in the rotation model of Scotese (2008) (as published by Cao et al., 2018). (c) Zonal mean of the absolute values of these deviations for all timeslices of the *P*_*pCO2_1000ppm*_ pathway, resolved by latitude and geologic time. For this, zonal mean data from all 5 Myr timeslices were aggregated with each timeslice represented by one grid column. The blue (red) contour lines illustrate times and periods with relatively high (low) zonal contrasts. (d) Global mean of the absolute values of these deviations for different boundary conditions.

To systematically characterize the evolution of zonal temperature contrasts on the continents through the Mesozoic, the deviation of local annual mean SATs from their zonal mean values is calculated. For two selected timeslices of the *P*_*pCO2_1000ppm*_ pathway, the resulting patterns are shown in Figures 6a and 6b. As described above, the eastern tropics and the western mid‐ to high‐latitudes of Pangaea are warmer by up to ∼8°C compared to the respective western or eastern regions (Figure 6a). For the fragmented late Cretaceous continental configuration, these east–west contrasts are reduced (Figure 6b). Figure 6c summarizes the zonal mean of the absolute value of these deviations for all considered geologic timeslices of the *P*_*pCO2_1000ppm*_ pathway. This indicates that zonal temperature contrasts are amplified in the mid–high latitudes as well as the tropical‐subtropical latitudes throughout the Triassic‐Jurassic but are clearly reduced after the Middle Jurassic. More uniform zonal temperature patterns were thus established in the mid‐Mesozoic while they were considerably perturbed by the supercontinent configuration before. This trend is also indicated by the global average of the absolute SAT deviations from their zonal mean (Figure 6d), which is interpreted as an indicator of global temperature “azonality”. It peaks at ∼2.2°C in the Late Triassic and Early Jurassic and is halved in the Late Cretaceous. Changes in boundary conditions other than paleogeography only lead to relatively small deviations from this trend. Our simulations thus illustrate a general trend toward decreased east–west temperature contrasts through the Mesozoic, mainly driven by continental fragmentation.

### 3.5 Aridity

**Figure 7:**
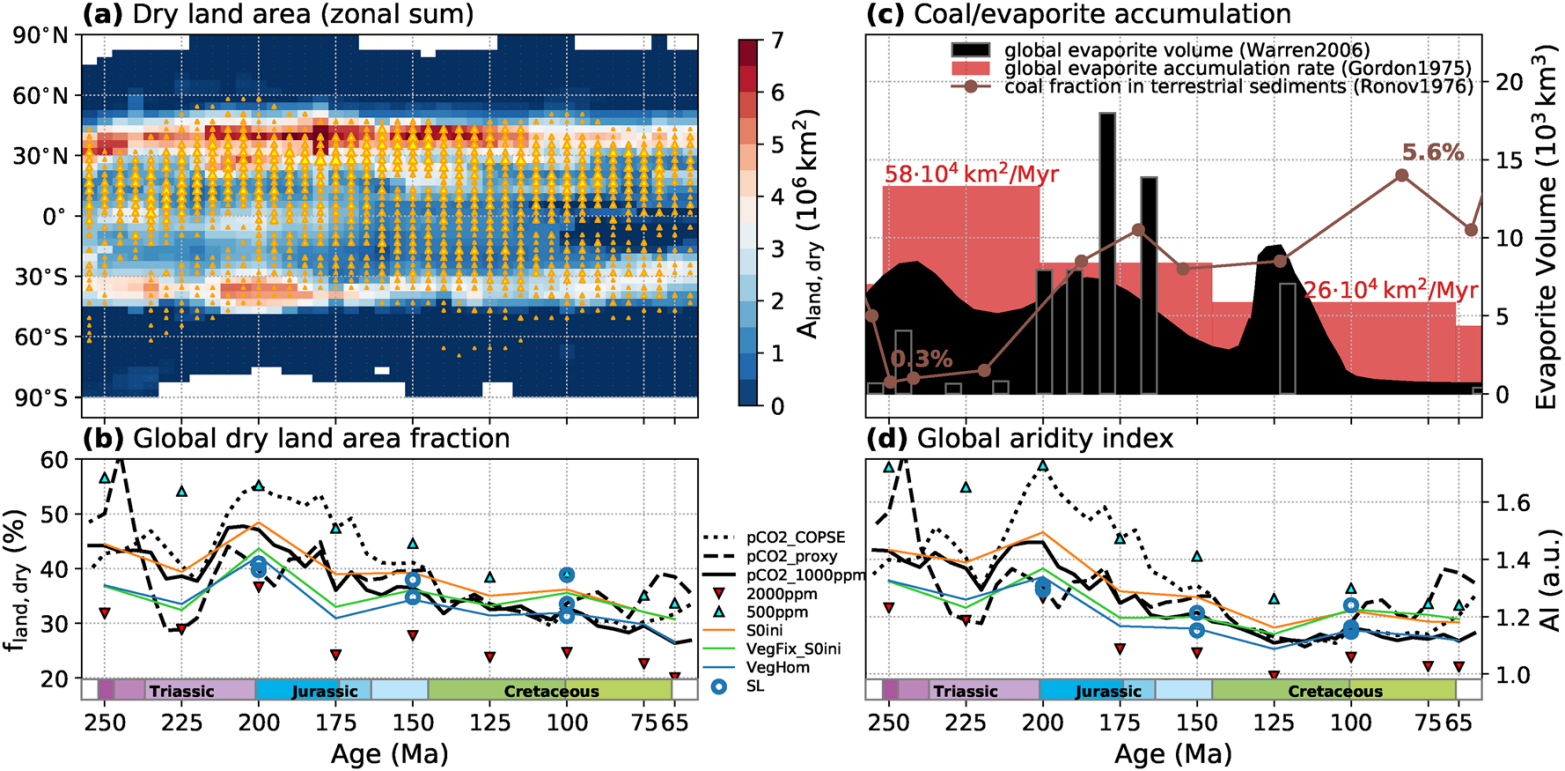
Extent (a and b) and intensity (d) of simulated dry continental climate conditions through the Mesozoic. (a) Zonal sum of dry land area (aridity index AI < 1.5, see Section 3.5) for all timeslices of the *P*_*pCO2_1000ppm*_ pathway (also see hatched areas in Figure 4). Triangles indicate the occurrence and frequency of evaporites in the data set of Boucot et al. (2013) at a given time and latitude. The size of the triangles scales with the number of occurrences in the respective time interval and latitudinal band (normalized with respect to its area). (b) Global fraction of land area experiencing dry climate conditions (AI < 1.5) for different boundary condition pathways. (d) Global aridity index based on the global mean values of precipitation and surface radiation balance on land. (c) Proxy estimates of global coal and evaporite accumulation (Gordon, 1975; Ronov et al., 1980; Warren, 2006).

Previous modeling work (Chaboureau et al., 2014; Donnadieu et al., 2009) suggested a reduction of the extent of arid climate conditions from the Late Triassic to the Late Cretaceous. To test this, we determine the simulated extent of relatively dry areas using the “radiative index of dryness” of Budyko (1974) which expresses the ratio of potential evaporation (RB/L) to precipitation (PRC): AI = RB/(L ⋅PRC) (Stadler, 2005). Here, RB is the annual mean net surface radiation balance and *L* is the latent heat of water evaporation and higher values of AI indicate drier conditions. The transition from humid forest climates to dry steppe climates occurs at values around 1.5 today (Budyko, 1974; Mabbutt, 1978). The changing extent of dry land area is indicated in Figure 4 (pink hatches) for four timeslices and in Figure 7a for all timeslices of the *P*_*pCO2_1000ppm*_ pathway. It can be seen in Figure 7a that the dry regions have their largest extent in the subtropical to mid‐latitudes during the Late Triassic‐Early Jurassic. In the low latitudes, dry regions are extended during the Triassic–Middle Jurassic, especially in western Pangaea (see Figure 4), but successively disappear in the further course, which indicates the establishment of more continuous tropical humid conditions. The relative portion of global arid land area on the *P*_*pCO2_1000ppm*_ pathway (Figure 7b) peaks in the latest Triassic and continuously decreases into the Late Cretaceous. In agreement with Chaboureau et al. (2014), the fraction of arid land is found to decrease with warming from elevated pCO_2_. The relatively low pCO_2_ suggested by the COPSE model reconstruction for the latest Triassic to Middle Jurassic would thus have significantly contributed to increased aridity during this time which would have amplified the humidification trend toward the mid‐Cretaceous. Also the continuous warming from increasing solar luminosity contributes to more humid conditions through the Mesozoic (Figure 7b, difference between *P*_*pCO2_1000ppm*_ and *P*_*S0ini*_). The cooling in the latest Cretaceous resulting from decreasing pCO_2_ in both considered reconstructions is found to increase aridity and to reverse the Early Jurassic to mid‐Cretaceous trend toward increasingly humid climates.

Coal and evaporite occurrences are frequently used as paleoclimatic indicators of humid or arid conditions (Markwick, 2007; Ziegler et al., 2003), and a certain agreement can be observed between their distributions and the humid and dry zones simulated here for our baseline pathway *P*_*pCO2_1000ppm*_ (Figure 4; proxy data from Boucot et al. (2013) and Cao et al. (2018), locations reconstructed with pyGPlates, see supporting information section 1, for further explanation). For example, many of the coal deposits occur in humid mid‐ to high‐latitude or tropical regions, while evaporites and calcretes are frequent in the relatively dry regions of the western Pangaean continents. This simple scheme can, however, not completely explain these spatial distributions (e.g., evaporites in tropical humid regions). This can be due to a variety of factors, including the range of possible local depositional environments and their variation on shorter (e.g., orbital) timescales. Considering these difficulties and the limitations of the employed climate model, a comprehensive assessment that takes into account the various boundary conditions is beyond the scope of this study. It can still be noted, that Cao et al. (2018) found a statistical Mesozoic–Cenozoic trend from an unimodal to a bimodal zonal distribution in the evaporite occurrence data of Boucot et al. (2013). Our simulations suggest that this could express the establishment of a more consistent tropical humid belt and two arid belts during the fragmentation of Pangaea (see Figure 4d), which could have displaced evaporite formation from equatorial regions especially in the eastern Panthalassa margin. The simulated overall trend toward more humid and less arid continental climates through the Mesozoic also conforms qualitatively with opposite trends of increasing coal versus decreasing evaporite deposition (Figure 7c).

## 4 Discussion

More than 200 equilibrium climate states for varied boundary conditions (paleogeography, sea level, vegetation patterns, pCO_2_, solar constant S_0_, and orbital configuration) were simulated and analyzed in this study, among other things, to obtain constraints on long‐term global mean temperature trends through the Mesozoic. Considering a recent proxy pCO_2_ reconstruction (Foster et al., 2017), the simulated global mean temperatures are elevated above pre‐industrial values for most of the Mesozoic and exceed 20°C in the Late Triassic‐Early Jurassic and mid‐Cretaceous. For the same proxy pCO_2_ reconstruction, Valdes et al. (2020) obtained similar global mean temperature trends, but up to 4°C higher absolute temperatures (Figure 1a). This expresses the higher climate sensitivity of the HadCM3 model, which is on the upper end of the likely 1.5°C–4.5°C range suggested by Stocker et al. (2014) (Farnsworth, Lunt, O'Brien, et al., 2019). The climate sensitivity exhibited by CLIMBER‐3α is more moderate in this regard, and compares reasonably with that of other Earth System Models of Intermediate Complexity (EMICs; Eby et al., 2013; Pfister & Stocker, 2017). In our simulations the GMST increase in response to a pCO_2_ doubling from 500 to 1,000 ppm ranges from 2.8°C for the warm late Cretaceous (at 75 Ma) to 4.1°C for the cooler Late Triassic (225 Ma). At similar pCO_2_ concentrations, Chaboureau et al. (2014) obtained slightly less elevated Mesozoic temperatures with their model (Figure 1a) but their best‐guess temperature curve agrees reasonably with that of our *P*_*pCO2_COPSE*_ pathway. Together, results from these three models illustrate the large range of uncertainty for simulated Mesozoic global mean temperatures which overall range from ∼14°C to 25°C. The gray shaded area in Figure 1a indicates the temperature envelope spanned by the different models and pCO_2_ pathways. Overall, warm temperature anomalies appear in the Late Triassic to earliest Jurassic and the late Early Cretaceous to early Late Cretaceous, while relatively lower temperatures are suggested for the Early‐Middle Triassic, parts of the Early Jurassic‐Early Cretaceous and the latest Cretaceous. To first order, these trends roughly conform with those inferred from proxy data (Figure 1c and Mills et al., 2019) and the occurrence of glacial deposits (Figure 1a), cyan colored bars, from Boucot et al., 2013 and Cao et al., 2018), which mainly occur in the middle Mesozoic and the latest Cretaceous and are mostly absent during the Triassic and mid‐Cretaceous.

One of the major challenges in the study of warm climate periods, for example, in the Cretaceous (e.g., Laugié et al., 2020; O'Brien et al., 2017; Tabor et al., 2016) or the early Eocene (e.g., Huber & Caballero, 2011; Lunt et al., 2012; Zhu et al., 2019), is the discrepancy between more strongly elevated global temperatures with low meridional gradients inferred from proxies and the conditions simulated by climate models (e.g., Huber, 2012; Upchurch et al., 2015). Challenges lie, for example, in the correct calibration and conversion of proxy data into local and global temperature estimates. In climate models, targeted modifications, for example, of atmospheric microphysical processes, can contribute to increased climate sensitivity and high‐latitude temperatures and thus an improved model‐proxy agreement at reconstructed pCO_2_ levels (Upchurch et al., 2015; Zhu et al., 2019). However, implementing and testing such modifications in CLIMBER‐3α was beyond the scope of this work. For comparison of simulated meridional temperature gradients, the same analysis as in Section 3.3 was performed with model output data from Valdes et al. (2020) and Farnsworth, Lunt, O'Brien, et al. (2019) (see supporting information section 7 with Figure S17). The zonal mean SAT profiles and the southern hemisphere high‐low latitudes contrast exhibit very similar patterns in the simulations of Valdes et al. (2020), which use the same paleogeographic reconstruction (compare Figure 5 and Figure S17). In the northern hemisphere, the HadCM3 gradient is slightly higher at comparable GMSTs but also shows a similar trend, only that it decreases more strongly toward the mid‐Cretaceous. This is probably mostly due to the more pronounced global warming due to increasing pCO_2_ (*P*_*pCO2_smooth*_ pathway, see Figure 1a). The trends in the Cretaceous simulations from Farnsworth, Lunt, O'Brien, et al. (2019) are quite different, which may derive from differences in the paleogeographic reconstruction, as they also employ the HadCM3 model. This indicates the need to assess different reconstructions and models in consistent intercomparison frameworks. Lapse‐rate corrected temperatures at sea level can provide a better comparability and can be obtained from the data repository for all CLIMBER‐3α simulations (also see Figure S12). Paleogeography was also identified as a major control of temperature gradients by Laugié et al. (2020), who compiled proxy and climate model estimates of zonal mean temperatures for the Cenomanian‐Turonian period (∼94 Ma). The gradients simulated here for this period fall within the range spanned by other models (Laugié et al., 2020), although some are closer to the lower gradients suggested by proxies (see supporting information section 7 and Figures  S16a and b). At ∼125 Ma, simulated SST gradients compare well with those from Steinig et al. (2020), who argue for an improved agreement with proxies when applying updated calibrations (also see Figures  S16c and S16d). We conclude that this study cannot solve the issues of high climate sensitivity and flat temperature gradients in warm climate periods, but offers a new systematic perspective on the effects of different changing boundary conditions, including paleography, through the Mesozoic.

To compare the continental SAT seasonality simulated here (see Section 3.2) with other available climate simulations, we performed the same analysis with results from Valdes et al. (2020, for *pCO*_2__*smooth*) and Farnsworth, Lunt, O'Brien, et al. (2019) (see Figure S22). In both studies, the globally averaged seasonality is systematically higher, but the data from Valdes et al. (2020) yields very similar trends, both of zonally and globally averaged seasonality (compare Figure 3 and Figure S22). This agreement, despite a different model set‐up and a slightly different global mean temperature evolution (see Figure 1a), supports the robustness of the presented trends at least for the paleogeographic reconstruction used in both studies. For a different reconstruction, the data from Farnsworth, Lunt, O'Brien, et al. (2019) also suggests a decrease of seasonality toward a minimum in the mid‐Late Cretaceous.

Our *P*_*SL*_ experiments demonstrate that long‐term sea‐level change could have contributed to a temperature rise on the order of 1°C from the Triassic‐Jurassic to the Late Cretaceous (see Section 3.1) and that also smaller scale sea‐level fluctuations can have a small, but notable effect on global climate characteristics if they significantly modify coastlines. As reconstructions of sea levels and paleo‐coastlines will be constantly revised by using additional data (e.g., Cao et al., 2017; Kocsis & Scotese, 2021), it appears important to reflect their uncertainty and variability in climate model simulations. Reconstructed maximum high‐ and low‐stand paleogeographies for each time‐interval could be particularly useful.

Depending on the respective model set‐up, deep‐time paleoclimate simulations require information on various boundary conditions, including paleogeography, pCO_2_, vegetation patterns, solar constant S_0_, and orbital configuration. In agreement with previous work (e.g., Donnadieu et al., 2006a; Donnadieu et al., 2009; Valdes et al., 2020), we find that these critically affect Earth's climatic evolution through the Mesozoic. However, large uncertainties are associated with these boundary conditions and it is essential to systematically assess their respective effects in climate model ensembles. EMICs like the CLIMBER‐3α model are flexible and computationally less demanding tools to perform large numbers of climate simulations with varied parameters, which are often not affordable with more comprehensive atmosphere‐ocean General Circulation Models. However, this comes at the cost of reduced complexity and spatial resolution. For the CLIMBER‐3α model this concerns especially its atmosphere module and its prescribed vegetation patterns. The latter do not dynamically respond to changes in other boundary conditions which can, for example, affect high‐latitude temperatures (e.g., Zhou et al., 2012; Hunter et al., 2013). However, proxy‐based vegetation patterns could sometimes be more realistic, despite large uncertainties, depending on the performance of the climate and vegetation models. The latter allow for a mechanistic assessment of climate‐vegetation feedbacks although they might not capture important long‐term evolutionary changes in terrestrial vegetation (Boyce & Lee, 2017). Here, we focus on certain large‐scale aspects of the Mesozoic climate evolution and systematic sensitivity tests for various boundary conditions. Further research with more comprehensive models is clearly required.

In our attempt to model a Mesozoic long‐term climate evolution, the most important factors appear to be pCO_2_ and paleogeography, both of which suffer from considerable uncertainties in the reconstructions. Model‐related uncertainties (e.g., in terms of climate sensitivity, the inability to model high‐latitude warmth or the treatment of vegetation) add to the difficulties. The understanding of mechanisms governing the Earth System in deep time and their representation in climate models is still limited. These uncertainties are ideally addressed by developing more comprehensive proxy reconstructions and as well as improved paleoclimate models and by systematically testing them in comprehensive intercomparison projects like DeepMIP (Hollis et al., 2019; Lunt et al., 2017, 2021).

It should be noted that the kind of study presented here provides sequences of equilibrium climate states which should be representative of long‐term climate trends on timescales of several million years. Here, the considered timeslices are spaced with 5 million years so that dynamics on shorter timescales cannot be represented. However, significant climatic variations occur within these timescales due to internal variability of the climate system as well as due to external factors like orbital cycles, related sea‐level fluctuations, meteorite impacts, volcanism, and solar activity changes. Therefore, the obtained long‐term trends can only be understood as a baseline around which Earth System states fluctuate. Individual empirical evidence may, however, often only reflect temporary conditions, for example, under a certain orbital configuration. The various sensitivity experiments, for example, with modified orbital configuration, indicate to a certain extent the degree of variation expected on these shorter time‐scales.

## 5 Conclusions

During the Mesozoic, Earth's climate experienced a fundamental transformation from the Pangaean supercontinent constellation into the Late Cretaceous with its fragmented continents and high sea levels. Here, we systematically assess aspects of the Mesozoic long‐term climate evolution in a continuous quantitative climate modeling framework. Simulations were performed for 40 timeslices, covering the time from 255 to 60 Ma in 5 Myr steps, using a recent paleogeographic reconstruction (Scotese & Wright, 2018). For each timeslice, multiple equilibrium climate states were simulated for varied boundary conditions, including pCO_2_, the solar constant, sea level, vegetation patterns, and orbital configuration. This ensemble was assessed here regarding global‐scale trends in the simulated global mean temperatures, seasonal, meridional, and zonal temperature contrasts and the extent of arid climate conditions.

Regarding the global mean temperatures, we find that paleogeographic changes, including rising sea levels, as well as increasing solar luminosity and changing vegetation patterns provided a baseline warming trend of ≈+3.5°C from the Late Triassic to the Late Cretaceous. The two considered pCO_2_ reconstructions do suggest deviations of up to 5°C from this baseline. These simulations, together with results from the few comparable climate model studies (Chaboureau et al., 2014; Donnadieu et al., 2009; Valdes et al., 2020) and proxy estimates (e.g., Mills et al., 2019; Veizer & Prokoph, 2015), support warm anomalies in the Triassic and mid‐Cretaceous. However, the spread resulting from uncertain long‐term pCO_2_ changes and the model climate sensitivity remains large, so that the different models and pCO_2_ pathways yield Mesozoic global mean temperatures in the range of ∼14°C–25°C. The average seasonality of continental SAT is found to be highest in Late Triassic and is significantly reduced toward the Late Cretaceous, driven by the changing extent and distribution of land masses. However, we find that different orbital configurations could have modified the globally averaged SAT seasonality to a similar degree on the timescales of orbital cycles. Regarding the high‐ to low‐latitude temperature gradients, our simulations indicate that these varied with the changing extent of land area in the high latitudes. In the baseline scenario with constant pCO_2_, the northern hemisphere gradient thus increases through the Triassic and falls in the course of the Cretaceous, while the southern hemisphere experiences the opposite trend. However, the thermal gradients also vary on a similar scale with global mean temperature, due to the snow and sea‐ice albedo feedback, with higher pCO_2_ and solar luminosity contributing to smaller gradients. We also observe an overall reduction of east‐west contrasts in the continental annual mean SATs from the Late Triassic‐Early Jurassic to the Late Cretaceous related to continental fragmentation. In agreement with previous studies, continental aridity is most widespread during the Late Triassic‐Early Jurassic and is reduced toward the Late Cretaceous. Our simulations do suggest, that not only continental fragmentation and rising sea levels contributed to this trend, but that higher global mean temperatures, for example, from elevated pCO_2_ and solar luminosity generally enhance humidity on the continents. Relatively low pCO_2_ levels suggested for parts of the Jurassic could thus have enhanced aridity and amplified the humidifying trend toward the Late Cretaceous, together with the subtle effect of warming from the rising solar luminosity.

This study aims to contribute to a consolidated picture of long‐term Mesozoic climate change. The presented ensemble of simulated climate states illustrates aspects of Mesozoic climate transition from a strongly seasonal, azonal and arid Pangaean climate toward a more balanced and humid Late Cretaceous warm climate. Existing concepts of Mesozoic long‐term climate change can overall be supported in this systematic ensemble approach, using an intermediate complexity Earth System Model. The provided model data (Landwehrs et al., 2021) are expected to prove useful for further investigations.

## Conflict of Interest

The authors declare that they have no conflict of interest.

## Supporting information

Supporting Information S1

## Data Availability

The presented data derive from simulations with the CLIMBER‐3α climate model (Montoya et al., 2005) with the boundary conditions described in Section 2. The model input and output data sets, the scripts used to generate the figures in the paper as well as additional maps for all simulation runs are available at the institutional repository of the Potsdam Institute for Climate Impact Research (Landwehrs et al., 2021, https://doi.org/10.5880/PIK.2020.009).
