# Supplementary material for: Investigating Mesozoic Climate Trends and Sensitivities With a Large Ensemble of Climate Model Simulations
Source: Paleoceanogr Paleoclimatol. 2021 Jun 5;36(6):e2020PA004134. doi: 10.1029/2020PA004134 (PMC8251552; doi:10.1029/2020PA004134)
Supplement: Supplementary file 1 — Supporting Information S1 [file PALO-36-0-s001.pdf]

# Supporting Information for "Investigating Mesozoic Climate Trends and Sensitivities with a Large Ensemble of Climate Model Simulations"

Jan Landwehrs[1,2], Georg Feulner[2], Stefan Petri[2], Benjamin Sames[1] and Michael Wagreich[1]

[1]Department of Geology, University of Vienna, Althanstraße 14, 1090 Vienna, Austria

[2]Earth System Analysis, Potsdam Institute for Climate Impact Research, Member of the Leibniz Association, P.O. Box 601203, D-14412 Potsdam, Germany

**Contents of this file**

1. Sections 1–10
2. Table S1
3. Figures S1–S22

**Additional Supporting Information (Files uploaded separately):**
See data repository at

`https://doi.org/10.5880/PIK.2020.009`

Corresponding author: J. Landwehrs, jan.landwehrs@univie.ac.at

## 1. Boundary Conditions: Orography, Bathymetry and Vegetation (Figs. S1–S3)

The topography (see Fig. S1) and bathymetry (see Fig. S2) implemented in the model simulations are based on the paleogeographic reconstructions of Scotese and Wright (2018). After interpolating onto the ocean model grid, the following necessary adjustments were made: Narrow straits with less than two grid cell widths were closed or widened. Isolated ocean cells needed to be removed or connected to the global ocean. To improve model stability, the minimum ocean depth was increased to 461 m and the upper-/lowermost grid row were set to a uniform median depth. Also, random variations of the deepest sea floor were introduced where it was completely flat in the original elevation model. The adjustments were kept as small and consistent as possible. As this study assesses global scale surface climates over a long geological time span, it was beyond the scope of this work to review specific features like ocean gateway configurations with regard to proxy evidence. Given the uncertainties in the paleogeographic reconstructions itself, such a review would certainly be warranted when focusing on the connection between tectonic changes, ocean circulation and climate for specific time periods and geographical regions.

For Fig. 4 and Fig. 7b, locations at the respective timeslice were reconstructed with pyGPlates using the data and the (Scotese, 2008) paleorotation model provided by (Cao et al., 2018). For Fig. 7b, the occurrences were sampled on the $3.75\times3.75\,^{\circ}$ grid and the size of the triangles scales with the frequency of the occurrences on this grid. Because the paleogeography changes within the relatively broad time-bins of the proxy data, the locations show a certain drift for timeslices within these time-bins.

## 2. Spin-Up (Figs. S4–S6)

Fig. S4 shows GMST timeseries (Fig. S4 during the 5000 yr spin-up for all simulations.

For a subset of runs ($\mathtt{P}_{\mathtt{pCO2\_1000ppm}}$ for $\mathtt{T}_{\mathtt{25Myr}}$), Fig. S6 shows global mean ocean seawater temperature and salinity at different depths over time.

For the same subset, Fig. S5 shows the relation of global mean top-of-the-atmosphere radiation balance (RBTP) and GMST during spin-up.

Fig. S6 shows global mean seawater temperature and salinity at three depth levels during spinup.

Similar plots for each individual run are included in the accompanying data repository (AllRuns_Spinup.pdf).

## 3. Atmosphere Fields (Figs. S7–S11)

Figures S7 to S10 illustrate the simulated surface air temperatures, precipitation and surface radiation balance for the four exemplary timeslices 75, 125, 175 and 225 Ma presented in the main article figures (similar maps for all simulation runs are included in the data repository). Figure S11 indicates the simulated change of precipitation and the surface radiation balance on land through time.

## 4. Global Mean Sea-Level Temperatures (Fig. S12)

Fig. S12 shows simulated global mean surface air temperatures compared to lapse-rate corrected global mean temperatures at sea-level for the $\mathtt{P_{pCO2\_1000ppm}}$ and the $\mathtt{P_{VegFix\_S0ini}}$ pathways.

## 5. Correlation of Global Mean Temperatures with the Total Area and Distribution of Land (Fig. S13)

Fig. S13 shows the correlation of lapse-rate corrected global mean air temperatures at sea level ($GMT_{SL}$) with the global land area fraction (a) and the average absolute latitude of land area (b). For the simulations of the $\mathtt{P_{VegFix\_S0ini}}$ pathway.

## 6. Proxy and Model Temperature Estimates (Figs. S14–S15)

Climate models inherently yield self-constrained global temperature fields, but might miss important processes that shaped these in reality. Proxy approaches often aim to compile consistent and globally distributed datasets to generate meridional or global SST or SAT distributions to obtain GMST estimates. Retrieving and calibrating such datasets is challenging and converting them into GMSTs is non-trivial. For example, the GMST estimates from (Mills et al., 2019, Fig 4b) (also see Fig. 1c of the present article) were mainly obtained from reconstructed tropical SST anomalies via a scaling factor of 1.5 (Mills et al., 2019):

$$\Delta \mathrm{GMST} = \mathrm{GMST_{ref}} + 1.5 \cdot \Delta \mathrm{SST_{trop}} \tag{1}$$

Where $\mathrm{GMST_{ref}} = 15\,°\mathrm{C}$ and $\Delta \mathrm{SST_{trop}} = -4.55 \cdot \delta^{18}\mathrm{O_{trop}}$ (Mills et al., 2019).

We tested the correlation of $\mathrm{SST_{trop}}$ and GMST (see Fig. S14a) in our model ensemble and indeed found an overall solid linear relationship (see Fig. S15a). However, we obtain a scaling factor of $\sim$1.79, indicating a stronger amplification of temperature changes at higher latitudes. Our results also suggest that the scaling is slightly higher for cooler climate states (see Fig. S15b), which could to some extent explain its variation through geologic time (Fig. S15c). For the latter panel, the correlation was calculated separately for the 9 $\mathtt{T_{25Myr}}$ timeslices and their climate states at $pCO_2$ =

$[250, 500, 1000, 1500, 2000, 4000]$ ppm. The 75 Ma timeslice, which is warm-biased by other boundary conditions (e.g. $S_0$, paleogeography), exhibits a slightly smaller scaling factor compared to the cold-biased 200 Ma timeslice (Fig. S15c, see inset).

Using the mean scaling factor of 1.79 and a reference GMST of 15 °C (as in Mills et al., 2019), we can calculate tropical SST anomalies for our simulations (Fig. S14b). These can be compared with the results from (Mills et al., 2019). (For this, we had to scale their GMST envelope back to $\Delta SST_{trop}$ via Eq. 1, as the authors did not report the underlying low-latitude SST anomalies in the same form.) As described in Sec. 3.1 of the paper, there appears to be a qualitative agreement on a slight cooling towards the end-Triassic to Early Jurassic, a warming towards the mid-Cretaceous, and a cooling at the end of the Cretaceous. However, the proxy estimates of $\Delta SST_{trop}$ are amplified by a factor of 2 or 3 compared to those in our model (note the different y-axis scales in Fig. S14b). As discussed, this can have several reasons, including a too low model climate sensitivity or uncertainties in the proxy temperature calibration. In future work, we aim at more comprehensive proxy–model comparisons than could be achieved within the framework of this study.

## 7. Meridional Temperature Gradients (Figs. S16–S17)

Simulated zonal mean SAT and SST profiles for the 95 and 125 Ma timeslices are shown in Fig. S16 for comparison with Laugié et al. (2020) and Steinig et al. (2020) as discussed in the main text (Sec. 4). Similar plots for all timeslices are included in the accompanying data repository. The figures also indicate numerical values of the global, low latitude (<30 °), high latitude (>60 °) averages and the difference between the latter two (upper left corner). As in Laugié et al. (2020) the meridional temperature gradient was also determined as the average change in °C per ° lat. via a linear regression between 30 and 80 ° latitude (upper right corner). The figures also include zonal mean temperatures calculated from model output from Valdes, Scotese, and Lunt (2020) and Farnsworth et al. (2019a).

For comparison with Fig. 5, zonal mean SAT and low–high latitude SAT differences are shown for data from Valdes et al. (2020) and Farnsworth et al. (2019a) in Fig. S17.

## 8. Meridional Heat Transport (Fig. S18)

Fig. S18 shows the change of the zonal and global mean meridional atmospheric/ocean/total heat transport through time. On a global average, the MHT varies less than ±0.2 PW around ∼2.6 PW between all simulations. It tends to be increased in cooler states, for low-obliquity orbital configurations (which are also cooler) and for homogeneous vegetation patterns. Three phases with relative strengthening ocean MHT in the northern (until ∼220 Ma), southern (∼220–110 Ma) and again northern (∼110–60 Ma)

hemisphere can be identified. To some degree, these periods also appear to coincide to with enhanced overturning (see Fig. S19 and AllRuns_OceanCrossections.pdf) and mixing in the respective hemisphere (see Fig. S21).

## 9. Ocean Fields (Figs. S19–S21)

Fig. S19 include meridional ocean cross-sections showing the annual mean ocean temperatures as a zonal mean, at 180 °W and 60 °E. They also show the global mean meridional overturning streamfunction. Similar plots for every simulation of the whole ensemble are included in the data repository (AllRuns_OceanCrosssections.pdf).

Fig. S20 show annual mean sea surface temperatures, surface salinity and mixed layer depth for simulations of the $P_{pCO2\_1000ppm}$ pathway. Similar plots for every simulation of the whole ensemble are included in the data repository (AllRuns_Maps.pdf).

Fig. S21 shows the zonal and annual mean mixed layer depth through time, aggregated for the $P_{pCO2\_1000ppm}$ pathway.

## 10. Seasonality (Fig. S22)

The same analysis of continental SAT seasonality as presented in Sec. 3.2 of the main paper was performed with modeling results from Valdes et al. (2020) and Farnsworth et al. (2019a) (see Fig. S22).

# 11. Table of runs

Table S1: List of all equilibrium climate simulations included in the analyses.

| | Run | Age (Ma) | $pCO_2$ (ppm) | $pCO_2$ pathway | $S_0$ ($W/m^2$) | Land fraction (%) | $T_{ann}$ (°C) | $PRC_{ann}$ (mm/a) | $T_{ann,trend}$ (°C/500yrs) | Radiation imbalance ($W/m^2$) |
|---|---|---|---|---|---|---|---|---|---|---|
| 0 | 060Ma_400ppm | 60 | 400 | proxy | 1354.7 | 31.7 | 16.7 | 1022 | 0.015 | 0.042 |
| 1 | 060Ma_500ppm | 60 | 500 | COPSE | 1354.7 | 31.7 | 17.7 | 1059 | -0.044 | 0.065 |
| 2 | 060Ma_1000ppm | 60 | 1000 | | 1354.7 | 31.7 | 20.6 | 1178 | 0.002 | 0.022 |
| 3 | 065Ma_250ppm | 65 | 250 | | 1354.1 | 31.3 | 14.7 | 954 | 0.003 | 0.033 |
| 4 | 065Ma_300ppm | 65 | 300 | proxy | 1354.1 | 31.3 | 15.5 | 984 | -0.003 | 0.028 |
| 5 | 065Ma_500ppm | 65 | 500 | | 1354.1 | 31.3 | 17.7 | 1070 | 0.000 | 0.035 |
| 6 | 065Ma_600ppm | 65 | 600 | COPSE | 1354.1 | 31.3 | 18.5 | 1101 | 0.003 | 0.022 |
| 7 | 065Ma_1000ppm | 65 | 1000 | | 1354.1 | 31.3 | 20.7 | 1188 | -0.004 | 0.04 |
| 8 | 065Ma_1000ppm_S0ini | 65 | 1000 | | 1332.7 | 31.3 | 18.8 | 1101 | -0.057 | 0.027 |
| 9 | 065Ma_1000ppm_S0ini_VegFix | 65 | 1000 | | 1332.7 | 31.3 | 18.8 | 1092 | 0.006 | 0.013 |
| 10 | 065Ma_1000ppm_VegHom | 65 | 1000 | | 1354.1 | 31.3 | 20.9 | 1195 | 0.004 | 0.048 |
| 11 | 065Ma_1000ppm_orb_22p0-0p00-000 | 65 | 1000 | | 1354.1 | 31.3 | 20.5 | 1191 | 0.007 | 0.039 |
| 12 | 065Ma_1000ppm_orb_24p5-0p06-090 | 65 | 1000 | | 1354.1 | 31.3 | 20.9 | 1195 | 0.015 | 0.094 |
| 13 | 065Ma_1000ppm_orb_24p5-0p06-180 | 65 | 1000 | | 1354.1 | 31.3 | 21.0 | 1198 | 0.006 | 0.025 |
| 14 | 065Ma_1000ppm_orb_24p5-0p06-270 | 65 | 1000 | | 1354.1 | 31.3 | 20.9 | 1197 | 0.012 | 0.029 |
| 15 | 065Ma_1500ppm | 65 | 1500 | | 1354.1 | 31.3 | 22.2 | 1256 | 0.009 | 0.039 |
| 16 | 065Ma_2000ppm | 65 | 2000 | | 1354.1 | 31.3 | 23.3 | 1302 | 0.012 | 0.023 |
| 17 | 065Ma_4000ppm | 65 | 4000 | | 1354.1 | 31.3 | 25.6 | 1405 | 0.016 | 0.133 |
| 18 | 070Ma_300ppm | 70 | 300 | proxy | 1353.5 | 32.2 | 15.5 | 983 | 0.004 | -0.001 |
| 19 | 070Ma_800ppm | 70 | 800 | COPSE | 1353.5 | 32.2 | 19.7 | 1142 | 0.004 | 0.036 |
| 20 | 070Ma_1000ppm | 70 | 1000 | | 1353.5 | 32.2 | 20.6 | 1180 | 0.006 | 0.037 |
| 21 | 075Ma_250ppm | 75 | 250 | | 1352.9 | 26.9 | 15.4 | 985 | 0.027 | 0.003 |
| 22 | 075Ma_500ppm | 75 | 500 | proxy | 1352.9 | 26.9 | 18.4 | 1096 | 0.001 | 0.032 |
| 23 | 075Ma_900ppm | 75 | 900 | COPSE | 1352.9 | 26.9 | 20.8 | 1193 | 0.006 | 0.04 |
| 24 | 075Ma_1000ppm | 75 | 1000 | | 1352.9 | 26.9 | 21.2 | 1211 | -0.015 | 0.054 |
| 25 | 075Ma_1000ppm_S0ini | 75 | 1000 | | 1332.7 | 26.9 | 19.5 | 1130 | 0.000 | 0.025 |
| 26 | 075Ma_1000ppm_S0ini_VegFix | 75 | 1000 | | 1332.7 | 26.9 | 19.4 | 1111 | -0.010 | 0.036 |
| 27 | 075Ma_1000ppm_VegHom | 75 | 1000 | | 1352.9 | 26.9 | 21.3 | 1209 | 0.008 | 0.055 |
| 28 | 075Ma_1000ppm_orb_22p0-0p00-000 | 75 | 1000 | | 1352.9 | 26.9 | 21.1 | 1214 | -0.000 | 0.038 |
| 29 | 075Ma_1000ppm_orb_24p5-0p06-090 | 75 | 1000 | | 1352.9 | 26.9 | 21.5 | 1217 | 0.010 | 0.064 |
| 30 | 075Ma_1000ppm_orb_24p5-0p06-180 | 75 | 1000 | | 1352.9 | 26.9 | 21.5 | 1221 | 0.001 | 0.025 |
| 31 | 075Ma_1000ppm_orb_24p5-0p06-270 | 75 | 1000 | | 1352.9 | 26.9 | 21.4 | 1221 | 0.016 | 0.027 |
| 32 | 075Ma_1500ppm | 75 | 1500 | | 1352.9 | 26.9 | 22.7 | 1276 | 0.009 | 0.081 |
| 33 | 075Ma_2000ppm | 75 | 2000 | | 1352.9 | 26.9 | 23.7 | 1321 | 0.025 | 0.107 |
| 34 | 075Ma_4000ppm | 75 | 4000 | | 1352.9 | 26.9 | 26.0 | 1422 | 0.012 | 0.169 |
| 35 | 080Ma_700ppm | 80 | 700 | proxy | 1352.4 | 26.0 | 19.8 | 1149 | -0.009 | 0.075 |
| 36 | 080Ma_900ppm | 80 | 900 | COPSE | 1352.4 | 26.0 | 20.8 | 1192 | -0.005 | 0.036 |
| 37 | 080Ma_1000ppm | 80 | 1000 | | 1352.4 | 26.0 | 21.2 | 1209 | 0.018 | 0.02 |
| 38 | 085Ma_600ppm | 85 | 600 | proxy | 1351.8 | 27.1 | 19.1 | 1122 | -0.038 | 0.019 |
| 39 | 085Ma_900ppm | 85 | 900 | COPSE | 1351.8 | 27.1 | 20.7 | 1190 | 0.001 | 0.036 |
| 40 | 085Ma_1000ppm | 85 | 1000 | | 1351.8 | 27.1 | 21.1 | 1208 | 0.005 | 0.041 |
| 41 | 090Ma_500ppm | 90 | 500 | proxy | 1351.2 | 28.0 | 18.1 | 1086 | 0.147 | 0.087 |
| 42 | 090Ma_900ppm | 90 | 900 | COPSE | 1351.2 | 28.0 | 20.5 | 1185 | 0.007 | 0.049 |
| 43 | 090Ma_1000ppm | 90 | 1000 | | 1351.2 | 28.0 | 20.9 | 1203 | 0.016 | 0.044 |
| 44 | 095Ma_600ppm | 95 | 600 | proxy | 1350.6 | 28.7 | 18.5 | 1099 | 0.004 | 0.045 |
| 45 | 095Ma_1000ppm | 95 | 1000 | COPSE | 1350.6 | 28.7 | 20.6 | 1185 | -0.001 | 0.036 |
| 46 | 100Ma_250ppm | 100 | 250 | | 1350.0 | 30.0 | 14.4 | 949 | -0.009 | 0.016 |
| 47 | 100Ma_500ppm | 100 | 500 | | 1350.0 | 30.0 | 17.4 | 1060 | -0.016 | 0.016 |
| 48 | 100Ma_900ppm | 100 | 900 | proxy | 1350.0 | 30.0 | 19.8 | 1158 | 0.003 | 0.072 |
| 49 | 100Ma_1000ppm | 100 | 1000 | COPSE | 1350.0 | 30.0 | 20.2 | 1176 | 0.002 | 0.068 |
| 50 | 100Ma_1000ppm_S0ini | 100 | 1000 | | 1332.7 | 30.0 | 18.8 | 1106 | 0.000 | 0.026 |
| 51 | 100Ma_1000ppm_S0ini_VegFix | 100 | 1000 | | 1332.7 | 30.0 | 18.7 | 1098 | -0.005 | -0.06 |
| 52 | 100Ma_1000ppm_SL+40 | 100 | 1000 | | 1350.0 | 29.7 | 20.4 | 1180 | 0.142 | 0.031 |
| 53 | 100Ma_1000ppm_SL-200 | 100 | 1000 | | 1350.0 | 37.9 | 19.1 | 1120 | 0.014 | 0.015 |
| 54 | 100Ma_1000ppm_SL-40 | 100 | 1000 | | 1350.0 | 30.7 | 20.1 | 1172 | 0.005 | 0.034 |
| 55 | 100Ma_1000ppm_VegHom | 100 | 1000 | | 1350.0 | 30.0 | 20.3 | 1184 | 0.005 | 0.068 |
| 56 | 100Ma_1000ppm_VegHom_SL+40 | 100 | 1000 | | 1350.0 | 29.7 | 20.5 | 1188 | 0.074 | 0.046 |
| 57 | 100Ma_1000ppm_VegHom_SL-200 | 100 | 1000 | | 1350.0 | 37.9 | 19.1 | 1124 | -0.013 | 0.059 |
| 58 | 100Ma_1000ppm_VegHom_SL-40 | 100 | 1000 | | 1350.0 | 30.7 | 20.2 | 1180 | 0.000 | 0.01 |
| 59 | 100Ma_1000ppm_orb_22p0-0p00-000 | 100 | 1000 | | 1350.0 | 30.0 | 20.1 | 1178 | 0.005 | 0.067 |
| 60 | 100Ma_1000ppm_orb_24p5-0p06-090 | 100 | 1000 | | 1350.0 | 30.0 | 20.5 | 1187 | 0.007 | 0.043 |
| 61 | 100Ma_1000ppm_orb_24p5-0p06-180 | 100 | 1000 | | 1350.0 | 30.0 | 20.5 | 1185 | 0.011 | 0.051 |
| 62 | 100Ma_1000ppm_orb_24p5-0p06-270 | 100 | 1000 | | 1350.0 | 30.0 | 20.5 | 1183 | 0.007 | 0.045 |
| 63 | 100Ma_1500ppm | 100 | 1500 | | 1350.0 | 30.0 | 21.8 | 1243 | 0.025 | 0.081 |
| 64 | 100Ma_2000ppm | 100 | 2000 | | 1350.0 | 30.0 | 22.8 | 1289 | 0.017 | 0.114 |
| 65 | 100Ma_4000ppm | 100 | 4000 | | 1350.0 | 30.0 | 25.1 | 1394 | 0.011 | 0.158 |
| 66 | 105Ma_1000ppm | 105 | 1000 | proxy | 1349.5 | 30.5 | 20.3 | 1178 | 0.005 | 0.081 |
| 67 | 105Ma_1100ppm | 105 | 1100 | COPSE | 1349.5 | 30.5 | 20.7 | 1195 | -0.001 | 0.077 |
| 68 | 110Ma_1000ppm | 110 | 1000 | | 1348.9 | 30.6 | 20.3 | 1161 | 0.001 | 0.035 |
| 69 | 110Ma_1100ppm | 110 | 1100 | proxyCOPSE | 1348.9 | 30.6 | 20.7 | 1177 | 0.002 | 0.027 |
| 70 | 115Ma_1000ppm | 115 | 1000 | COPSE | 1348.3 | 30.3 | 20.2 | 1152 | 0.002 | 0.052 |
| 71 | 115Ma_1100ppm | 115 | 1100 | proxy | 1348.3 | 30.3 | 20.6 | 1169 | 0.003 | 0.053 |
| 72 | 120Ma_1000ppm | 120 | 1000 | proxyCOPSE | 1347.7 | 30.4 | 19.8 | 1128 | -0.000 | 0.058 |
| 73 | 125Ma_250ppm | 125 | 250 | | 1347.2 | 31.1 | 13.5 | 900 | -0.003 | 0.033 |
| 74 | 125Ma_500ppm | 125 | 500 | | 1347.2 | 31.1 | 16.9 | 1014 | -0.000 | 0.027 |
| 75 | 125Ma_900ppm | 125 | 900 | proxy | 1347.2 | 31.1 | 19.5 | 1116 | 0.001 | 0.034 |
| 76 | 125Ma_1000ppm | 125 | 1000 | COPSE | 1347.2 | 31.1 | 19.9 | 1135 | 0.003 | 0.039 |
| 77 | 125Ma_1000ppm_S0ini | 125 | 1000 | | 1332.7 | 31.1 | 18.6 | 1074 | 0.000 | 0.032 |
| 78 | 125Ma_1000ppm_S0ini_VegFix | 125 | 1000 | | 1332.7 | 31.1 | 18.8 | 1090 | 0.001 | 0.042 |
| 79 | 125Ma_1000ppm_VegHom | 125 | 1000 | | 1347.2 | 31.1 | 20.1 | 1152 | 0.003 | 0.048 |
| 80 | 125Ma_1000ppm_orb_22p0-0p00-000 | 125 | 1000 | | 1347.2 | 31.1 | 19.8 | 1135 | 0.002 | 0.035 |
| 81 | 125Ma_1000ppm_orb_24p5-0p06-090 | 125 | 1000 | | 1347.2 | 31.1 | 20.2 | 1146 | 0.003 | 0.008 |
| 82 | 125Ma_1000ppm_orb_24p5-0p06-180 | 125 | 1000 | | 1347.2 | 31.1 | 20.2 | 1149 | 0.003 | 0.026 |
| 83 | 125Ma_1000ppm_orb_24p5-0p06-270 | 125 | 1000 | | 1347.2 | 31.1 | 20.1 | 1147 | 0.003 | 0.015 |
| 84 | 125Ma_1500ppm | 125 | 1500 | | 1347.2 | 31.1 | 21.5 | 1206 | 0.006 | 0.062 |
| 85 | 125Ma_2000ppm | 125 | 2000 | | 1347.2 | 31.1 | 22.6 | 1255 | 0.009 | 0.083 |
| 86 | 125Ma_4000ppm | 125 | 4000 | | 1347.2 | 31.1 | 24.9 | 1367 | 0.021 | 0.136 |
| 87 | 130Ma_900ppm | 130 | 900 | proxyCOPSE | 1346.6 | 33.5 | 19.1 | 1092 | -0.015 | 0.023 |
| 88 | 130Ma_1000ppm | 130 | 1000 | | 1346.6 | 33.5 | 19.5 | 1111 | -0.004 | 0.022 |
| 89 | 135Ma_1000ppm | 135 | 1000 | proxyCOPSE | 1346.0 | 34.2 | 19.3 | 1103 | 0.002 | 0.017 |
| 90 | 140Ma_900ppm | 140 | 900 | proxyCOPSE | 1345.4 | 34.9 | 18.6 | 1077 | -0.001 | 0.01 |

| | | | | | | | | | | |
|---|---|---|---|---|---|---|---|---|---|---|
| 91 | 140Ma_1000ppm | 140 | 1000 | | 1345.4 | 34.9 | 19.0 | 1096 | -0.001 | 0.023 |
| 92 | 145Ma_700ppm | 145 | 700 | proxyCOPSE | 1344.8 | 34.3 | 17.5 | 1027 | -0.013 | 0.022 |
| 93 | 145Ma_1000ppm | 145 | 1000 | | 1344.8 | 34.3 | 19.1 | 1093 | -0.000 | 0.02 |
| 94 | 150Ma_250ppm | 150 | 250 | | 1344.3 | 34.2 | 12.0 | 825 | -0.003 | 0.004 |
| 95 | 150Ma_500ppm | 150 | 500 | | 1344.3 | 34.2 | 15.7 | 946 | -0.005 | -0.01 |
| 96 | 150Ma_700ppm | 150 | 700 | COPSE | 1344.3 | 34.2 | 17.3 | 1007 | -0.035 | 0.027 |
| 97 | 150Ma_800ppm | 150 | 800 | proxy | 1344.3 | 34.2 | 18.0 | 1032 | -0.009 | 0.023 |
| 98 | 150Ma_1000ppm | 150 | 1000 | | 1344.3 | 34.2 | 18.9 | 1072 | -0.004 | 0.034 |
| 99 | 150Ma_1000ppm_S0ini | 150 | 1000 | | 1332.7 | 34.2 | 17.9 | 1023 | -0.023 | 0.038 |
| 100 | 150Ma_1000ppm_S0ini_VegFix | 150 | 1000 | | 1332.7 | 34.2 | 18.3 | 1066 | 0.003 | 0.012 |
| 101 | 150Ma_1000ppm_SL+40 | 150 | 1000 | | 1344.3 | 33.6 | 19.1 | 1074 | -0.001 | 0.025 |
| 102 | 150Ma_1000ppm_SL-40 | 150 | 1000 | | 1344.3 | 36.5 | 18.6 | 1050 | 0.005 | 0.029 |
| 103 | 150Ma_1000ppm_VegHom | 150 | 1000 | | 1344.3 | 34.2 | 19.3 | 1110 | 0.001 | 0.016 |
| 104 | 150Ma_1000ppm_VegHom_SL+40 | 150 | 1000 | | 1344.3 | 33.6 | 19.5 | 1114 | -0.000 | 0.015 |
| 105 | 150Ma_1000ppm_VegHom_SL-40 | 150 | 1000 | | 1344.3 | 36.5 | 18.9 | 1087 | -0.007 | 0.022 |
| 106 | 150Ma_1000ppm_orb_22p0-0p00-000 | 150 | 1000 | | 1344.3 | 34.2 | 18.8 | 1071 | -0.003 | 0.031 |
| 107 | 150Ma_1000ppm_orb_24p5-0p06-090 | 150 | 1000 | | 1344.3 | 34.2 | 19.2 | 1084 | 0.001 | 0.024 |
| 108 | 150Ma_1000ppm_orb_24p5-0p06-180 | 150 | 1000 | | 1344.3 | 34.2 | 19.2 | 1086 | 0.001 | 0.007 |
| 109 | 150Ma_1000ppm_orb_24p5-0p06-270 | 150 | 1000 | | 1344.3 | 34.2 | 19.2 | 1084 | 0.004 | 0.016 |
| 110 | 150Ma_1500ppm | 150 | 1500 | | 1344.3 | 34.2 | 20.6 | 1143 | 0.002 | 0.046 |
| 111 | 150Ma_2000ppm | 150 | 2000 | | 1344.3 | 34.2 | 21.7 | 1193 | 0.010 | 0.074 |
| 112 | 150Ma_4000ppm | 150 | 4000 | | 1344.3 | 34.2 | 24.2 | 1309 | 0.016 | 0.093 |
| 113 | 155Ma_700ppm | 155 | 700 | COPSE | 1343.7 | 31.9 | 17.7 | 1018 | 0.057 | 0.043 |
| 114 | 155Ma_800ppm | 155 | 800 | proxy | 1343.7 | 31.9 | 18.3 | 1043 | 0.153 | 0.007 |
| 115 | 155Ma_1000ppm | 155 | 1000 | | 1343.7 | 31.9 | 19.3 | 1083 | -0.002 | 0.02 |
| 116 | 160Ma_600ppm | 160 | 600 | COPSE | 1343.1 | 28.2 | 17.3 | 1005 | 0.000 | 0.025 |
| 117 | 160Ma_800ppm | 160 | 800 | proxy | 1343.1 | 28.2 | 18.7 | 1059 | 0.001 | 0.032 |
| 118 | 160Ma_1000ppm | 160 | 1000 | | 1343.1 | 28.2 | 19.7 | 1101 | 0.000 | 0.034 |
| 119 | 165Ma_600ppm | 165 | 600 | COPSE | 1342.5 | 30.5 | 16.8 | 978 | 0.007 | 0.027 |
| 120 | 165Ma_900ppm | 165 | 900 | proxy | 1342.5 | 30.5 | 18.8 | 1059 | 0.157 | -0.029 |
| 121 | 165Ma_1000ppm | 165 | 1000 | | 1342.5 | 30.5 | 19.3 | 1078 | 0.000 | 0.007 |
| 122 | 170Ma_500ppm | 170 | 500 | COPSE | 1341.9 | 32.5 | 15.4 | 919 | -0.005 | 0.021 |
| 123 | 170Ma_1000ppm | 170 | 1000 | proxy | 1341.9 | 32.5 | 19.0 | 1053 | -0.000 | 0.023 |
| 124 | 175Ma_250ppm | 175 | 250 | | 1341.4 | 33.6 | 10.9 | 785 | 0.000 | 0.042 |
| 125 | 175Ma_500ppm | 175 | 500 | COPSE | 1341.4 | 33.6 | 15.0 | 914 | 0.001 | 0.024 |
| 126 | 175Ma_1000ppm | 175 | 1000 | proxy | 1341.4 | 33.6 | 18.7 | 1051 | -0.001 | 0.027 |
| 127 | 175Ma_1000ppm_S0ini | 175 | 1000 | | 1332.7 | 33.6 | 17.8 | 1012 | 0.027 | 0.035 |
| 128 | 175Ma_1000ppm_S0ini_VegFix | 175 | 1000 | | 1332.7 | 33.6 | 18.3 | 1065 | 0.001 | 0.012 |
| 129 | 175Ma_1000ppm_VegHom | 175 | 1000 | | 1341.4 | 33.6 | 19.0 | 1097 | 0.000 | 0.016 |
| 130 | 175Ma_1000ppm_orb_22p0-0p00-000 | 175 | 1000 | | 1341.4 | 33.6 | 18.6 | 1050 | 0.004 | 0.009 |
| 131 | 175Ma_1000ppm_orb_24p5-0p06-090 | 175 | 1000 | | 1341.4 | 33.6 | 18.9 | 1060 | -0.000 | 0.015 |
| 132 | 175Ma_1000ppm_orb_24p5-0p06-180 | 175 | 1000 | | 1341.4 | 33.6 | 19.0 | 1067 | -0.001 | 0.002 |
| 133 | 175Ma_1000ppm_orb_24p5-0p06-270 | 175 | 1000 | | 1341.4 | 33.6 | 19.0 | 1070 | -0.000 | -0.008 |
| 134 | 175Ma_1500ppm | 175 | 1500 | | 1341.4 | 33.6 | 20.4 | 1127 | 0.002 | 0.014 |
| 135 | 175Ma_2000ppm | 175 | 2000 | | 1341.4 | 33.6 | 21.6 | 1180 | -0.005 | 0.006 |
| 136 | 175Ma_4000ppm | 175 | 4000 | | 1341.4 | 33.6 | 24.1 | 1305 | -0.000 | 0.018 |
| 137 | 180Ma_500ppm | 180 | 500 | COPSE | 1340.9 | 38.2 | 14.1 | 865 | -0.009 | 0.017 |
| 138 | 180Ma_900ppm | 180 | 900 | proxy | 1340.9 | 38.2 | 17.4 | 980 | -0.007 | 0.001 |
| 139 | 180Ma_1000ppm | 180 | 1000 | | 1340.9 | 38.2 | 17.9 | 1000 | 0.005 | 0.015 |
| 140 | 185Ma_500ppm | 185 | 500 | COPSE | 1340.3 | 35.8 | 14.5 | 887 | 0.091 | -0.005 |
| 141 | 185Ma_900ppm | 185 | 900 | proxy | 1340.3 | 35.8 | 17.8 | 1005 | -0.002 | 0.006 |
| 142 | 185Ma_1000ppm | 185 | 1000 | | 1340.3 | 35.8 | 18.3 | 1026 | 0.018 | 0.005 |
| 143 | 190Ma_500ppm | 190 | 500 | COPSE | 1339.8 | 35.1 | 14.8 | 886 | 0.001 | 0.023 |
| 144 | 190Ma_1000ppm | 190 | 1000 | | 1339.8 | 35.1 | 18.5 | 1016 | -0.000 | 0.038 |
| 145 | 190Ma_1100ppm | 190 | 1100 | proxy | 1339.8 | 35.1 | 18.9 | 1033 | -0.000 | 0.033 |
| 146 | 195Ma_500ppm | 195 | 500 | COPSE | 1339.2 | 35.1 | 14.2 | 864 | 0.000 | 0.029 |
| 147 | 195Ma_1000ppm | 195 | 1000 | | 1339.2 | 35.1 | 18.1 | 1001 | -0.016 | 0.005 |
| 148 | 195Ma_1700ppm | 195 | 1700 | proxy | 1339.2 | 35.1 | 20.5 | 1098 | -0.114 | 0.059 |
| 149 | 200Ma_250ppm | 200 | 250 | | 1338.7 | 37.4 | 9.9 | 731 | 0.002 | 0.031 |
| 150 | 200Ma_500ppm | 200 | 500 | COPSE | 1338.7 | 37.4 | 13.9 | 847 | -0.010 | 0.028 |
| 151 | 200Ma_1000ppm | 200 | 1000 | | 1338.7 | 37.4 | 17.8 | 978 | 0.006 | 0.027 |
| 152 | 200Ma_1000ppm_S0ini | 200 | 1000 | | 1332.7 | 37.4 | 17.2 | 952 | 0.001 | 0.02 |
| 153 | 200Ma_1000ppm_S0ini_VegFix | 200 | 1000 | | 1332.7 | 37.4 | 17.6 | 1003 | -0.009 | 0.016 |
| 154 | 200Ma_1000ppm_SL+200 | 200 | 1000 | | 1338.7 | 32.0 | 19.0 | 1011 | -0.009 | 0.013 |
| 155 | 200Ma_1000ppm_SL+40 | 200 | 1000 | | 1338.7 | 36.0 | 18.1 | 990 | -0.017 | 0.016 |
| 156 | 200Ma_1000ppm_VegHom | 200 | 1000 | | 1338.7 | 37.4 | 18.2 | 1030 | 0.006 | 0.014 |
| 157 | 200Ma_1000ppm_VegHom_SL+200 | 200 | 1000 | | 1338.7 | 32.0 | 19.3 | 1060 | -0.043 | 0.078 |
| 158 | 200Ma_1000ppm_VegHom_SL+40 | 200 | 1000 | | 1338.7 | 36.0 | 18.5 | 1042 | -0.147 | 0.009 |
| 159 | 200Ma_1000ppm_orb_22p0-0p00-000 | 200 | 1000 | | 1338.7 | 37.4 | 17.7 | 972 | 0.007 | 0.038 |
| 160 | 200Ma_1000ppm_orb_24p5-0p06-090 | 200 | 1000 | | 1338.7 | 37.4 | 18.1 | 997 | 0.021 | 0.001 |
| 161 | 200Ma_1000ppm_orb_24p5-0p06-180 | 200 | 1000 | | 1338.7 | 37.4 | 18.1 | 996 | 0.002 | -0.009 |
| 162 | 200Ma_1000ppm_orb_24p5-0p06-270 | 200 | 1000 | | 1338.7 | 37.4 | 18.1 | 995 | 0.033 | -0.013 |
| 163 | 200Ma_1500ppm | 200 | 1500 | | 1338.7 | 37.4 | 19.7 | 1052 | -0.006 | 0.015 |
| 164 | 200Ma_1700ppm | 200 | 1700 | proxy | 1338.7 | 37.4 | 20.2 | 1074 | 0.019 | 0.023 |
| 165 | 200Ma_2000ppm | 200 | 2000 | | 1338.7 | 37.4 | 20.9 | 1102 | 0.005 | 0.024 |
| 166 | 200Ma_4000ppm | 200 | 4000 | | 1338.7 | 37.4 | 23.4 | 1223 | -0.017 | 0.044 |
| 167 | 205Ma_600ppm | 205 | 600 | COPSE | 1338.1 | 37.1 | 15.0 | 873 | 0.001 | 0.034 |
| 168 | 205Ma_1000ppm | 205 | 1000 | | 1338.1 | 37.1 | 17.8 | 971 | 0.005 | 0.016 |
| 169 | 205Ma_1500ppm | 205 | 1500 | proxy | 1338.1 | 37.1 | 19.6 | 1045 | 0.008 | 0.035 |
| 170 | 210Ma_800ppm | 210 | 800 | COPSE | 1337.6 | 36.6 | 16.6 | 925 | 0.043 | 0.051 |
| 171 | 210Ma_1000ppm | 210 | 1000 | | 1337.6 | 36.6 | 17.7 | 967 | -0.005 | 0.019 |
| 172 | 210Ma_1300ppm | 210 | 1300 | proxy | 1337.6 | 36.6 | 19.0 | 1017 | -0.034 | 0.004 |
| 173 | 215Ma_900ppm | 215 | 900 | COPSE | 1337.0 | 36.9 | 17.4 | 962 | 0.001 | 0.032 |
| 174 | 215Ma_1000ppm | 215 | 1000 | | 1337.0 | 36.9 | 17.9 | 982 | 0.000 | 0.018 |
| 175 | 215Ma_1300ppm | 215 | 1300 | proxy | 1337.0 | 36.9 | 19.2 | 1032 | 0.000 | 0.011 |
| 176 | 220Ma_1000ppm | 220 | 1000 | COPSE | 1336.5 | 30.6 | 18.2 | 1011 | 0.009 | 0.004 |
| 177 | 220Ma_1600ppm | 220 | 1600 | proxy | 1336.5 | 30.6 | 20.4 | 1101 | -0.005 | 0.013 |
| 178 | 225Ma_250ppm | 225 | 250 | | 1336.0 | 30.9 | 9.1 | 719 | 0.001 | 0.047 |
| 179 | 225Ma_500ppm | 225 | 500 | | 1336.0 | 30.9 | 13.8 | 844 | -0.000 | 0.06 |
| 180 | 225Ma_900ppm | 225 | 900 | COPSE | 1336.0 | 30.9 | 17.3 | 962 | 0.000 | -0.005 |
| 181 | 225Ma_1000ppm | 225 | 1000 | | 1336.0 | 30.9 | 17.9 | 983 | 0.001 | 0.003 |
| 182 | 225Ma_1000ppm_S0ini | 225 | 1000 | | 1332.7 | 30.9 | 17.5 | 968 | 0.000 | 0 |
| 183 | 225Ma_1000ppm_S0ini_VegFix | 225 | 1000 | | 1332.7 | 30.9 | 18.4 | 1052 | 0.000 | -0.021 |
| 184 | 225Ma_1000ppm_VegHom | 225 | 1000 | | 1336.0 | 30.9 | 18.3 | 1032 | 0.000 | -0.013 |
| 185 | 225Ma_1000ppm_orb_22p0-0p00-000 | 225 | 1000 | | 1336.0 | 30.9 | 17.7 | 981 | 0.000 | 0.005 |
| 186 | 225Ma_1000ppm_orb_24p5-0p06-090 | 225 | 1000 | | 1336.0 | 30.9 | 18.3 | 1003 | -0.000 | 0.005 |
| 187 | 225Ma_1000ppm_orb_24p5-0p06-180 | 225 | 1000 | | 1336.0 | 30.9 | 18.2 | 997 | 0.002 | 0.004 |
| 188 | 225Ma_1000ppm_orb_24p5-0p06-270 | 225 | 1000 | | 1336.0 | 30.9 | 18.3 | 993 | -0.004 | -0.025 |
| 189 | 225Ma_1500ppm | 225 | 1500 | | 1336.0 | 30.9 | 19.9 | 1059 | -0.006 | 0.002 |
| 190 | 225Ma_2000ppm | 225 | 2000 | proxy | 1336.0 | 30.9 | 21.1 | 1111 | -0.022 | 0.008 |
| 191 | 225Ma_4000ppm | 225 | 4000 | | 1336.0 | 30.9 | 23.6 | 1227 | 0.001 | 0.016 |
| 192 | 230Ma_800ppm | 230 | 800 | COPSE | 1335.4 | 31.6 | 16.5 | 936 | 0.001 | 0.008 |
| 193 | 230Ma_1000ppm | 230 | 1000 | | 1335.4 | 31.6 | 17.7 | 979 | 0.009 | -0.004 |

| | | | | | | | | | | |
|---|---|---|---|---|---|---|---|---|---|---|
| 194 | 230Ma_1900ppm | 230 | 1900 | proxy | 1335.4 | 31.6 | 20.7 | 1099 | -0.000 | 0.001 |
| 195 | 235Ma_800ppm | 235 | 800 | COPSE | 1334.9 | 32.0 | 16.6 | 934 | 0.010 | 0.038 |
| 196 | 235Ma_1000ppm | 235 | 1000 | | 1334.9 | 32.0 | 17.9 | 979 | 0.003 | -0.004 |
| 197 | 235Ma_1400ppm | 235 | 1400 | proxy | 1334.9 | 32.0 | 19.4 | 1040 | -0.003 | -0 |
| 198 | 240Ma_800ppm | 240 | 800 | proxy | 1334.3 | 31.7 | 16.5 | 942 | 0.026 | 0.008 |
| 199 | 240Ma_900ppm | 240 | 900 | COPSE | 1334.3 | 31.7 | 17.2 | 964 | 0.041 | 0.022 |
| 200 | 240Ma_1000ppm | 240 | 1000 | | 1334.3 | 31.7 | 17.8 | 984 | 0.004 | 0.016 |
| 201 | 245Ma_400ppm | 245 | 400 | proxy | 1333.8 | 31.3 | 12.9 | 818 | -0.001 | 0.049 |
| 202 | 245Ma_1000ppm | 245 | 1000 | COPSE | 1333.8 | 31.3 | 18.1 | 992 | 0.001 | -0.015 |
| 203 | 250Ma_250ppm | 250 | 250 | | 1333.2 | 30.8 | 10.3 | 740 | 0.001 | 0.016 |
| 204 | 250Ma_500ppm | 250 | 500 | | 1333.2 | 30.8 | 14.9 | 869 | -0.001 | 0.022 |
| 205 | 250Ma_700ppm | 250 | 700 | proxy | 1333.2 | 30.8 | 16.8 | 933 | 0.000 | 0.005 |
| 206 | 250Ma_1000ppm | 250 | 1000 | | 1333.2 | 30.8 | 18.5 | 998 | 0.001 | -0.009 |
| 207 | 250Ma_1000ppm_S0ini | 250 | 1000 | | 1332.7 | 30.8 | 18.5 | 996 | -0.011 | -0.01 |
| 208 | 250Ma_1000ppm_S0ini_VegFix | 250 | 1000 | | 1332.7 | 30.8 | 18.8 | 1040 | -0.003 | -0.009 |
| 209 | 250Ma_1000ppm_VegHom | 250 | 1000 | | 1333.2 | 30.8 | 18.8 | 1039 | -0.003 | -0.005 |
| 210 | 250Ma_1000ppm_orb_22p0-0p00-000 | 250 | 1000 | | 1333.2 | 30.8 | 18.4 | 995 | -0.005 | -0.005 |
| 211 | 250Ma_1000ppm_orb_24p5-0p06-090 | 250 | 1000 | | 1333.2 | 30.8 | 18.7 | 1023 | -0.048 | 0.021 |
| 212 | 250Ma_1000ppm_orb_24p5-0p06-180 | 250 | 1000 | | 1333.2 | 30.8 | 18.8 | 1016 | -0.012 | 0.008 |
| 213 | 250Ma_1000ppm_orb_24p5-0p06-270 | 250 | 1000 | | 1333.2 | 30.8 | 18.9 | 1008 | 0.014 | 0.005 |
| 214 | 250Ma_1100ppm | 250 | 1100 | COPSE | 1333.2 | 30.8 | 18.9 | 1015 | 0.003 | -0.011 |
| 215 | 250Ma_1500ppm | 250 | 1500 | | 1333.2 | 30.8 | 20.3 | 1070 | -0.001 | -0.02 |
| 216 | 250Ma_2000ppm | 250 | 2000 | | 1333.2 | 30.8 | 21.4 | 1120 | 0.009 | -0.011 |
| 217 | 250Ma_4000ppm | 250 | 4000 | | 1333.2 | 30.8 | 23.9 | 1238 | 0.002 | -0.011 |
| 218 | 255Ma_800ppm | 255 | 800 | proxy | 1332.7 | 29.6 | 17.6 | 967 | -0.001 | -0.004 |
| 219 | 255Ma_1000ppm | 255 | 1000 | | 1332.7 | 29.6 | 18.6 | 1007 | 0.002 | -0 |
| 220 | 255Ma_1300ppm | 255 | 1300 | COPSE | 1332.7 | 29.6 | 19.8 | 1054 | 0.002 | 0.016 |

## References

Boucot, A. J., Xu, C., Scotese, C. R., & Morley, R. J. (2013). *Phanerozoic Paleoclimate: An Atlas of Lithologic Indicators of Climate.* SEPM Society for Sedimentary Geology. doi: 10.2110/sepmcsp.11

Cao, W., Williams, S., Flament, N., Zahirovic, S., Scotese, C. R., & Müller, R. D. (2018). Palaeolatitudinal distribution of lithologic indicators of climate in a palaeogeographic framework. *Geological Magazine*, *156*(2), 331-354. doi: 10.1017/S0016756818000110

Farnsworth, A., Lunt, D. J., O'Brien, C. L., Foster, G. L., Inglis, G. N., Markwick, P., ... Robinson, S. A. (2019a). Climate Sensitivity on Geological Timescales Controlled by Nonlinear Feedbacks and Ocean Circulation. *Geophysical Research Letters*, *46*. doi: 10.1029/2019GL083574

Laugié, M., Donnadieu, Y., Ladant, J.-B., Green, J. A. M., Bopp, L., & Raisson, F. (2020). Stripping back the modern to reveal the Cenomanian–Turonian climate and temperature gradient underneath. *Climate of the Past*, *16*(3), 953–971. doi: 10.5194/cp-16-953-2020

Mills, B. J., Krause, A. J., Scotese, C. R., Hill, D. J., Shields, G. A., & Lenton, T. M. (2019). Modelling the long-term carbon cycle, atmospheric CO2, and Earth surface temperature from late Neoproterozoic to present day. *Gondwana Research*, *67*, 172 - 186. doi: 10.1016/j.gr.2018.12.001

Scotese, C. R. (2008). The PALEOMAP Project PaleoAtlas for ArcGIS, Volume 2, Cretaceous paleogeographic and plate tectonic reconstructions. *PALEOMAP Project, Arlington, Texas.*

Scotese, C. R. (2016). *Global Climate Change (Modern Times to 540 million years ago).* Retrieved from https://www.researchgate.net/publication/303811641_Global_Climate_Change_Modern_Times_to_540_million_years_ago

Scotese, C. R., & Wright, N. (2018). PALEOMAP paleodigital elevation models (PaleoDEMS) for the Phanerozoic. Retrieved from https://www.earthbyte.org/paleodem-resource-scotese-and-wright-2018

Steinig, S., Dummann, W., Park, W., Latif, M., Kusch, S., Hofmann, P., & Flögel, S. (2020). Evidence for a regional warm bias in the Early Cretaceous TEX86 record. *Earth and Planetary Science Letters*, *539*, 116184. doi: 10.1016/j.epsl.2020.116184

Valdes, P. J., Scotese, C. R., & Lunt, D. J. (2020). Deep Ocean Temperatures through Time. *Climate of the Past Discussions*, 1–37. doi: 10.5194/cp-2020-83

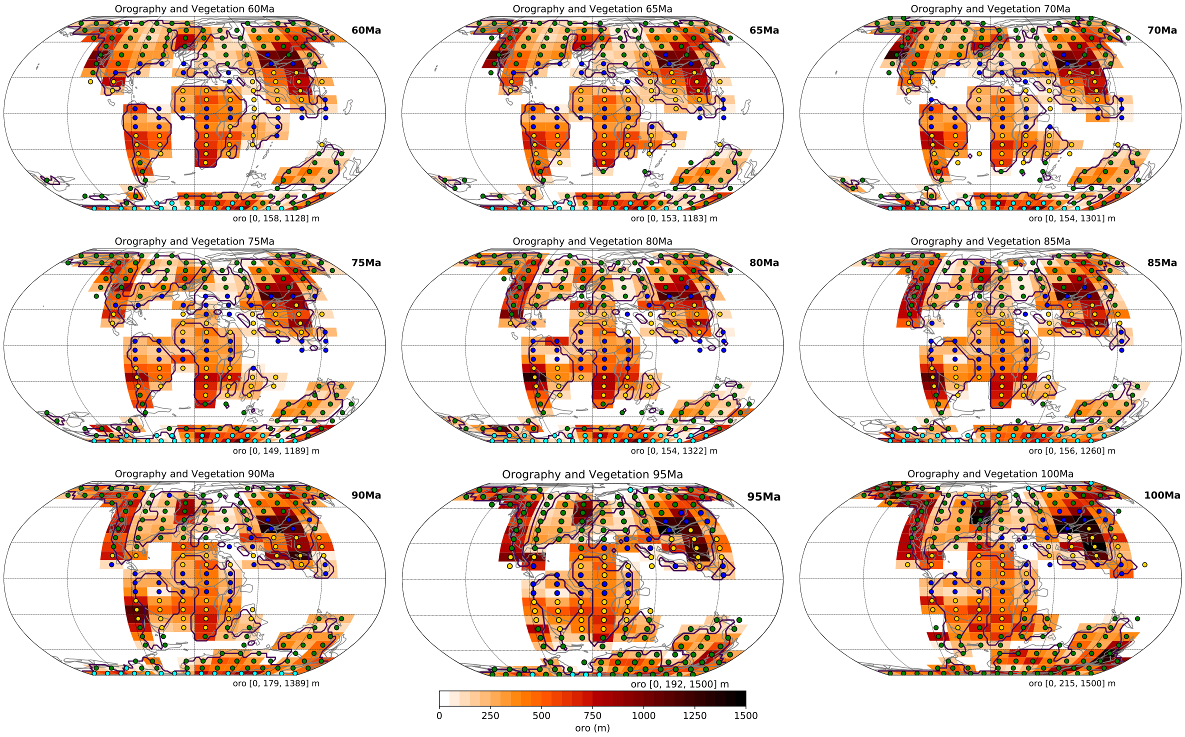


**Figure S1.** Orography (colors, based on Scotese and Wright (2018)) and Vegetation cover (dots) on the atmosphere model grid for all timeslices. The black lines indicate the land-sea mask on the ocean model grid resolution. Dots indicate the vegetation patterns (`VegHet`) approximated from the proxy-inferred climate zones from (Boucot et al., 2013; Scotese, 2016). Blue: tropical/boreotropical (75 % tree, 20 % grass/shrub, 5 % bare soil), yellow: arid (15 %, 35 %, 60 %), green: warm temperate (70 %, 20 %, 10 %), cyan: cold temperate (65 %, 15 %, 20 %), grey: polar (10 %, 25 %, 65 %)

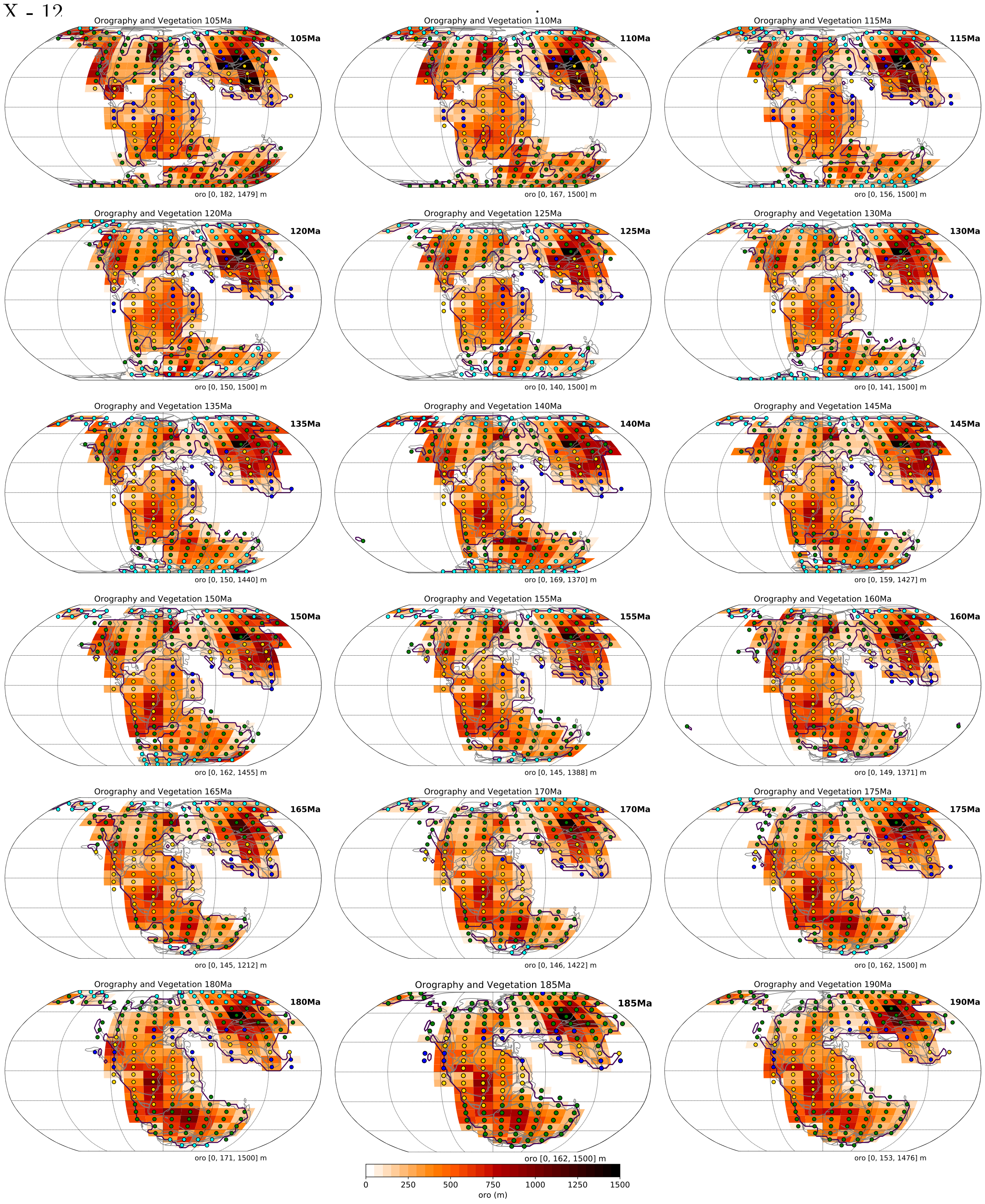


... Fig. S1 continued

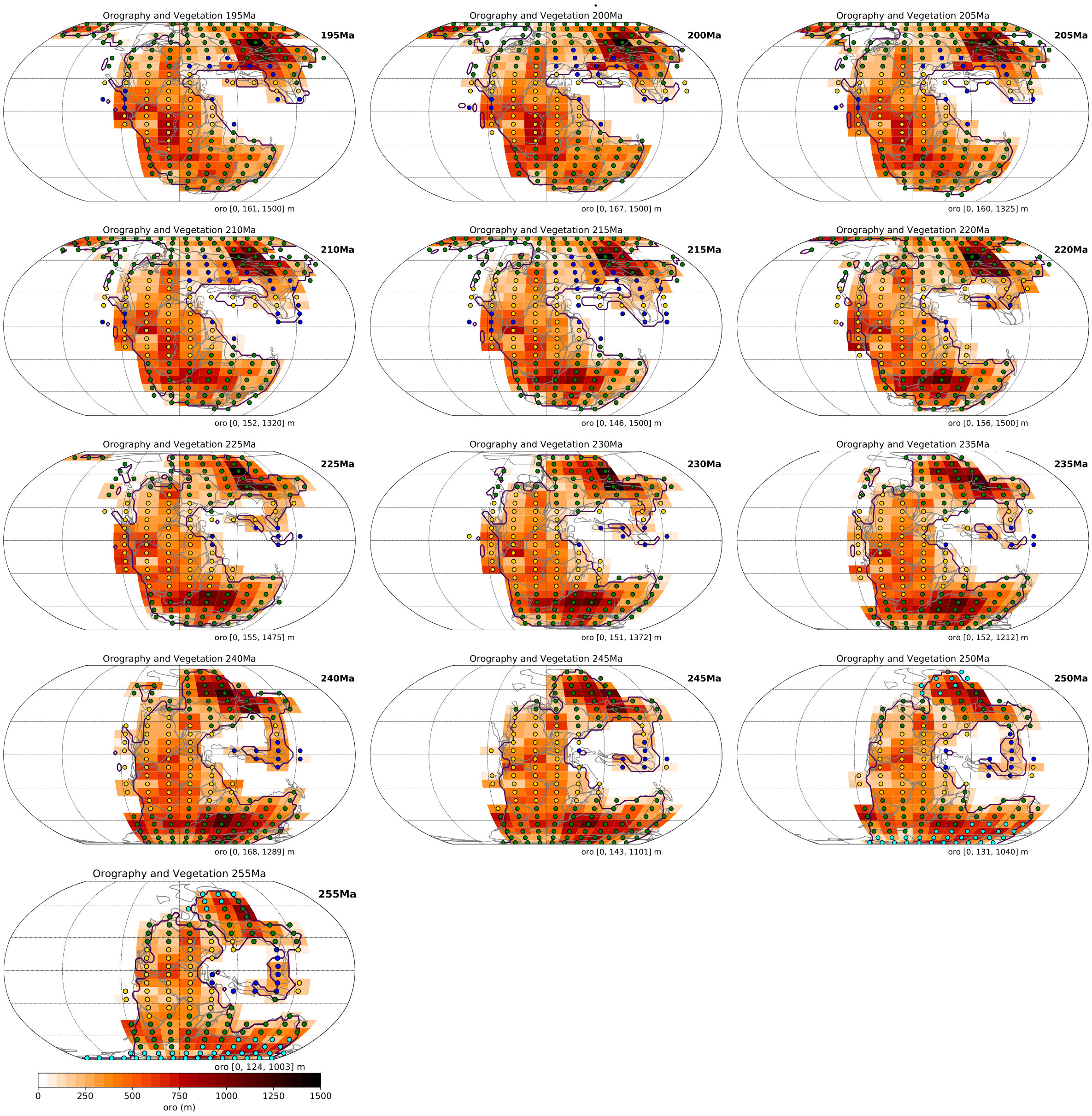


... Fig. S1 continued

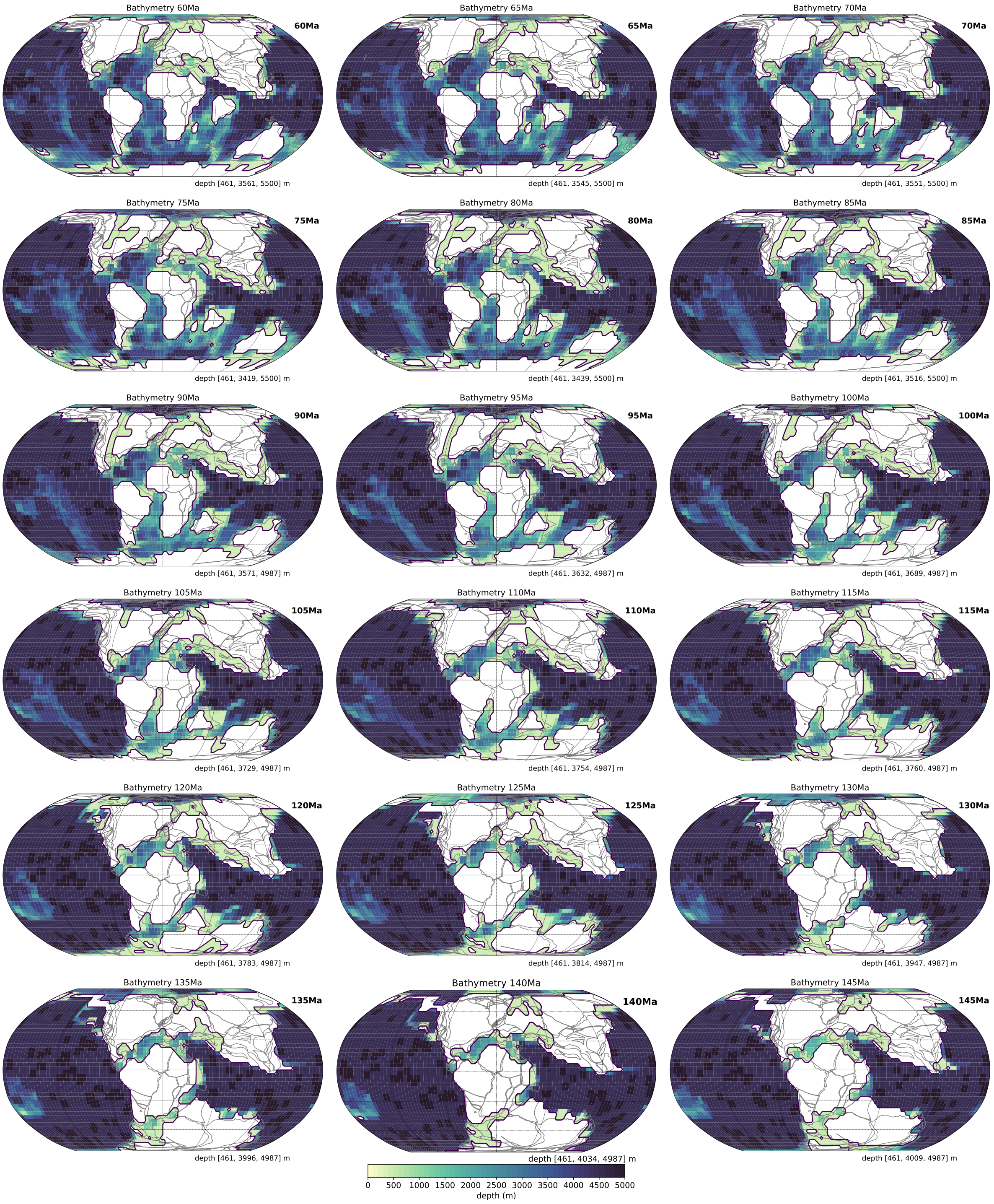


**Figure S2.** Bathymetry for all timeslices as implemented in the simulations based on Scotese and Wright (2018). Adjustments were made due to model requirements (see Supp. Sec. 1 for further explanation).

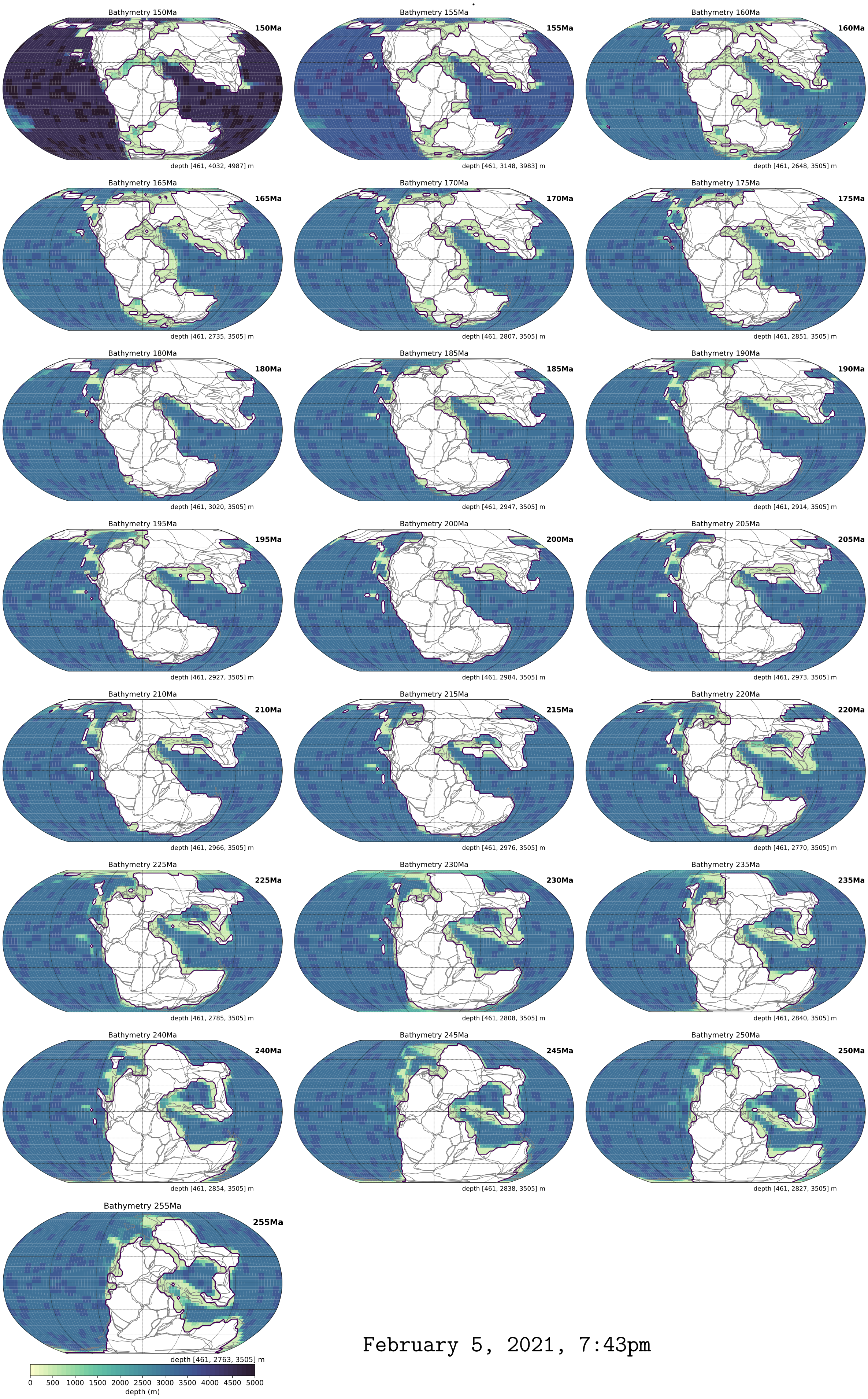




... Fig. S2 continued

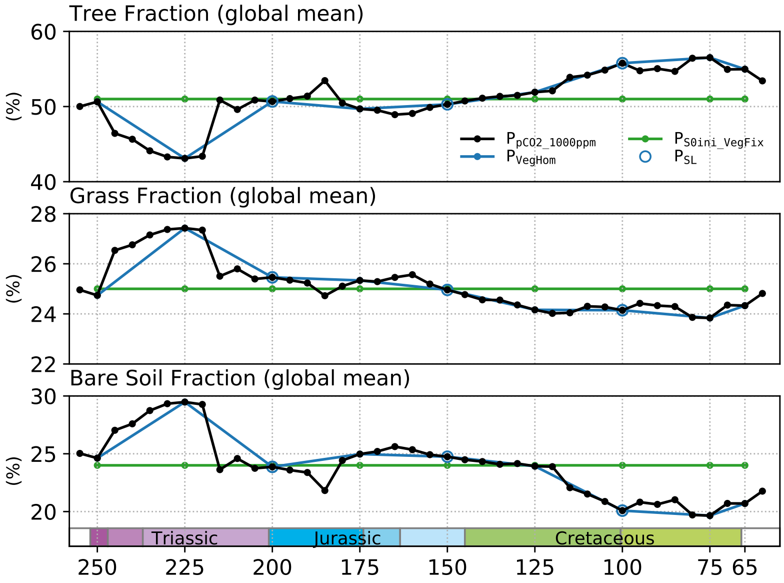


**Figure S3.** Global mean fractions of tree, grass/shrub and bare soil land cover fraction in different boundary condition pathways.

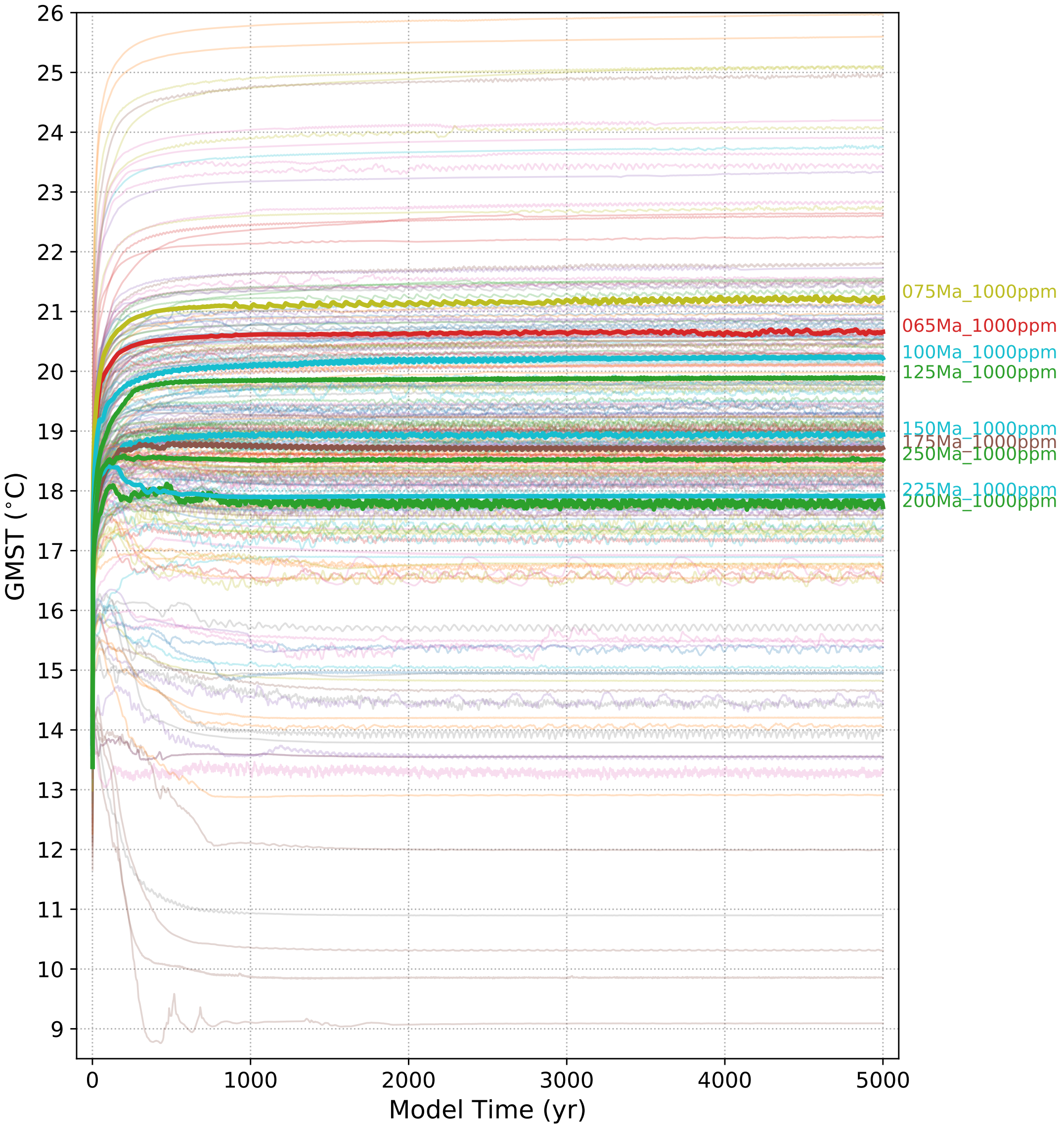


**Figure S4.** Global mean surface air temperature (GMST) during spin-up for all simulation runs. For the highlighted representative subset of runs ($\mathtt{P}_{\mathtt{pCO2\_1000ppm}}$ for $\mathtt{T}_{\mathtt{25Myr}}$), mean ocean temperatures and salinity timeseries are shown in Fig. S21.

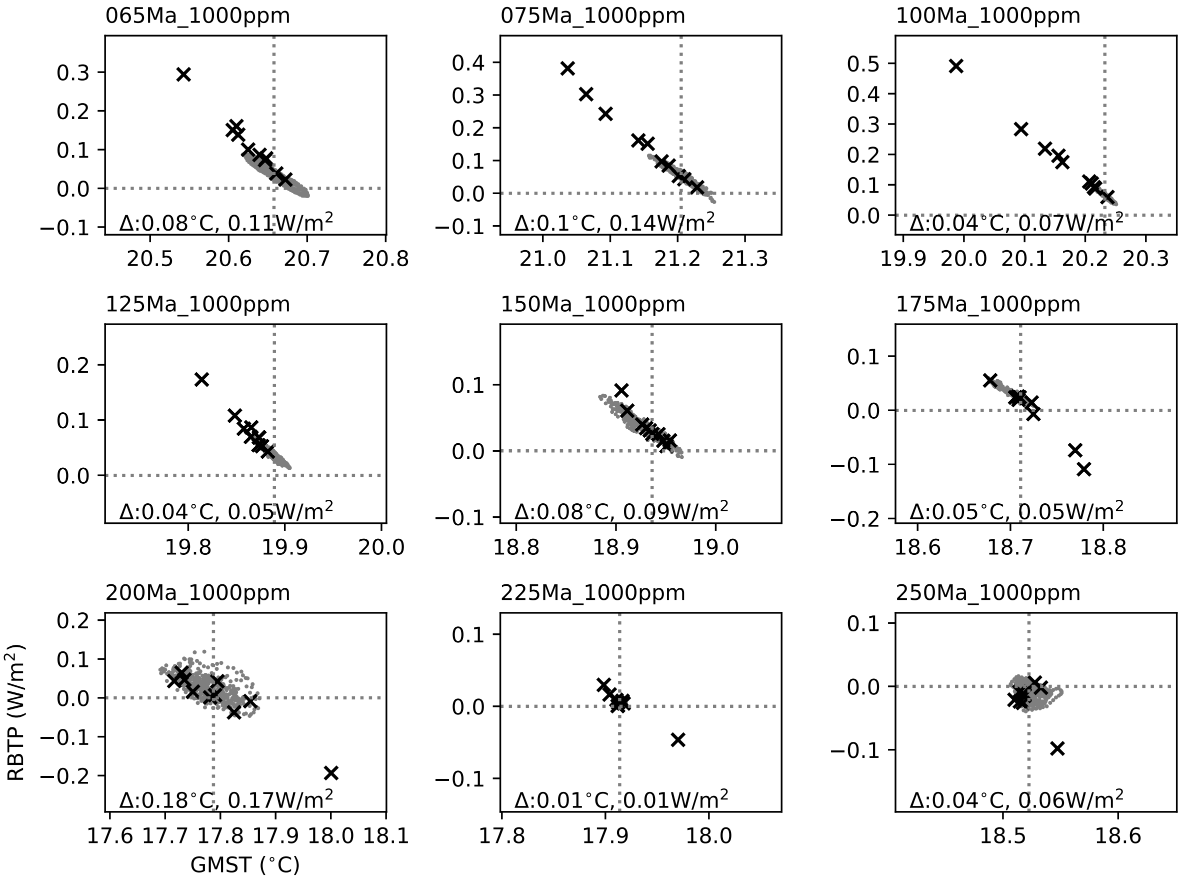


**Figure S5.** Global mean top-of-the-atmosphere radiation balance (RBTP) over the global mean surface air temperature (GMST) during spinup (Gregory Plot), for the $\mathtt{T_{25Myr}}$ timeslices of the $\mathtt{P_{pCO2\_1000ppm}}$ pathway. Crosses represent 500 yr timesteps from model year 500 to 5000, while grey dots represent annual data over the last 500 years of the simulations (years 4500–5000). The maximum variation of GMST and RBTP over the last 500 years is also given in each panel. The vertical dashed line indicates the average GMST over the last 500 years.

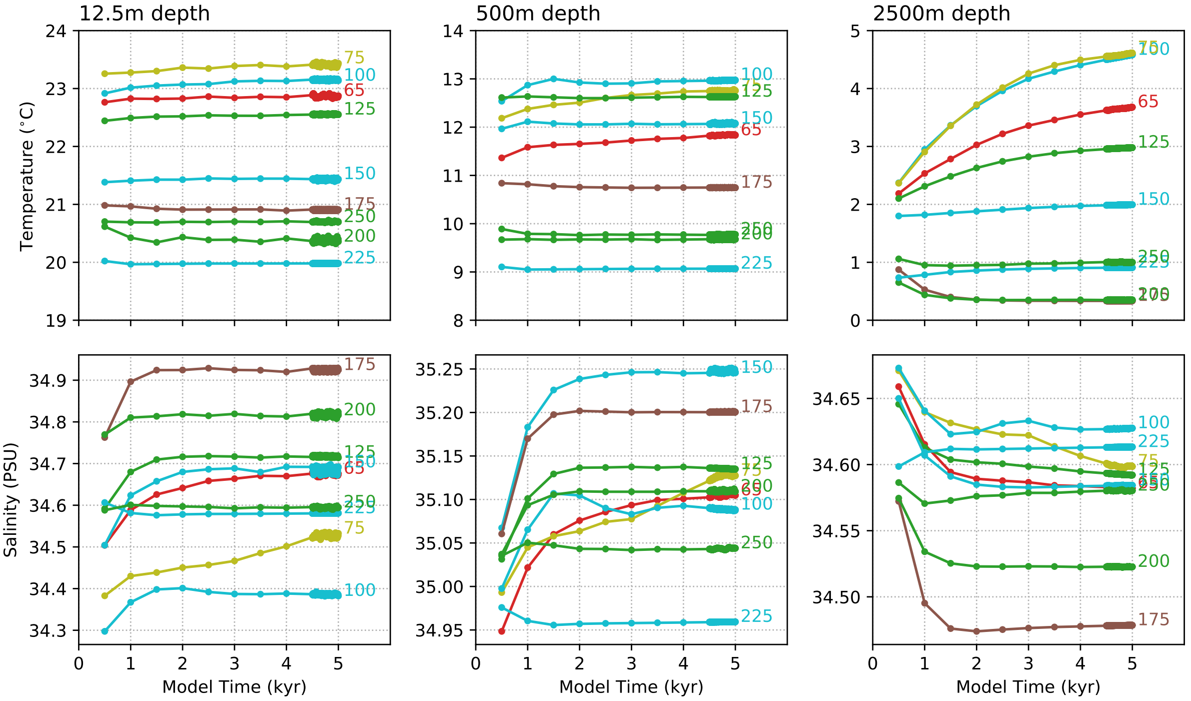


**Figure S6.** Global mean seawater temperature and salinity at three depth levels during spinup for a subset of the simulation runs ($P_{pCO2\_1000ppm}$ for $T_{25Myr}$, also see Figs. S4,S5).

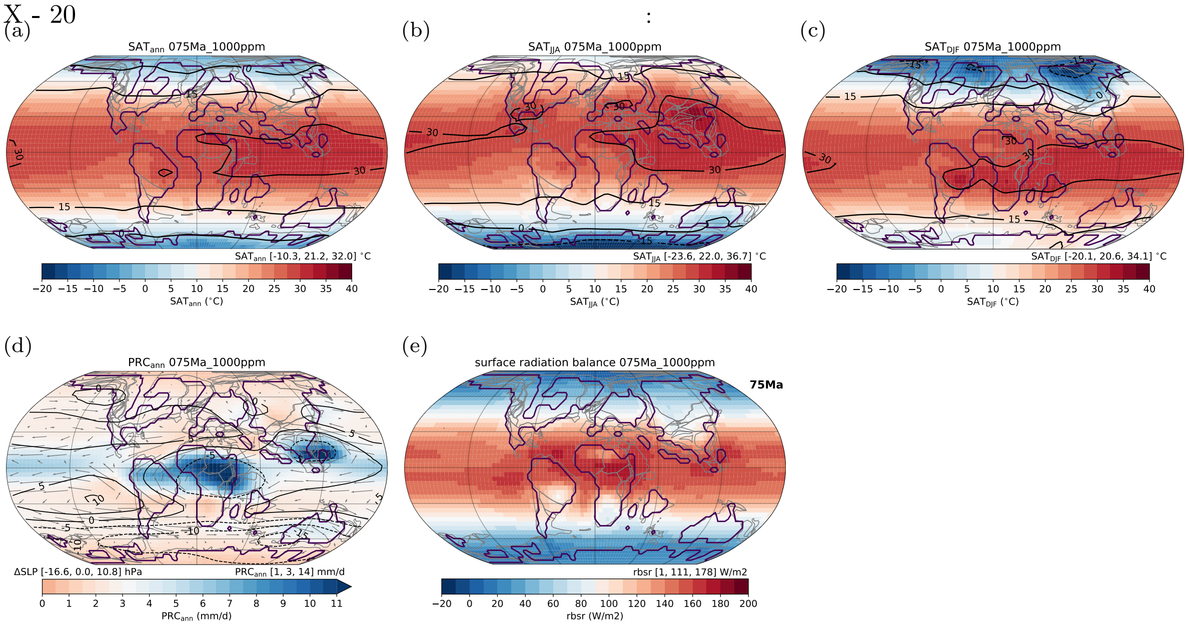


**Figure S7.** Annual mean surface air temperatures (a), precipitation rates (d) and surface radiation balance (e) for the $\mathtt{P_{pCO2\_1000ppm}}$ pathway at 75 Ma. Seasonal JJA and DJF surface air temperatures (b,c) for the same simulation. Similar plots are included in the accompanying data repository for all runs (AllRuns_Maps.pdf)

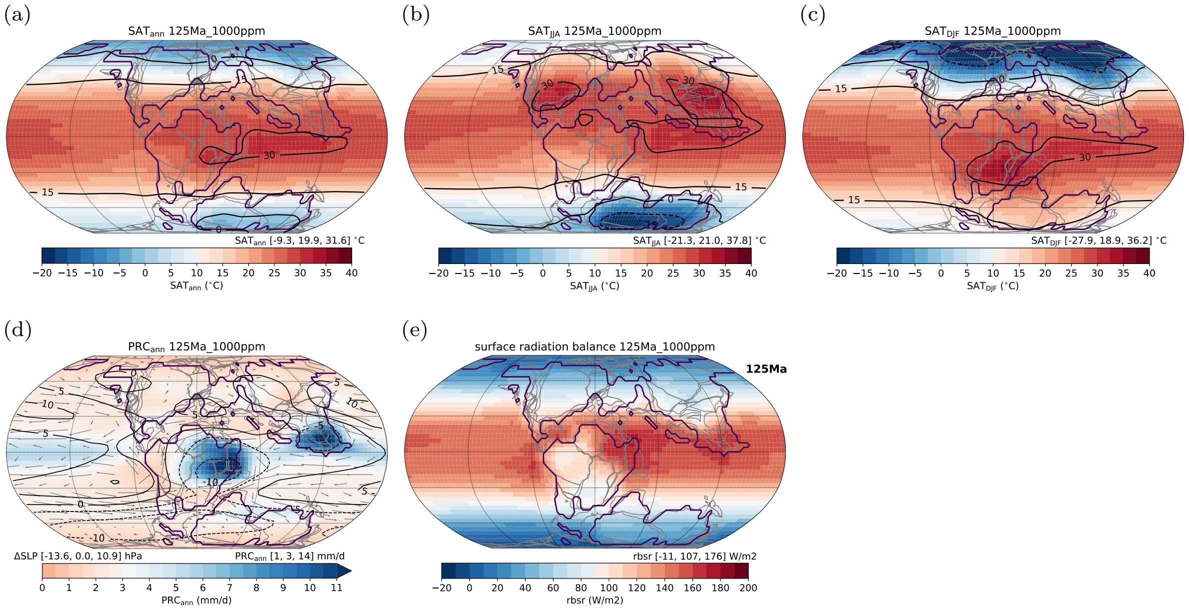


**Figure S8.** Same as Fig. S7 but at 125 Ma

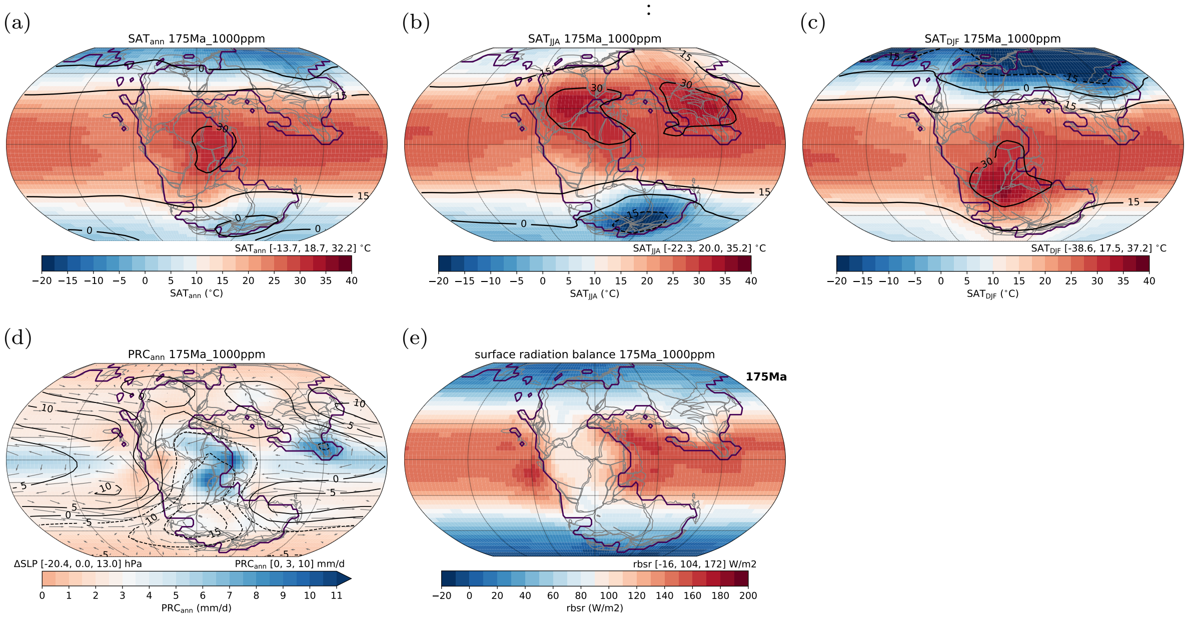


**Figure S9.** Same as Fig. S7 but at 175 Ma

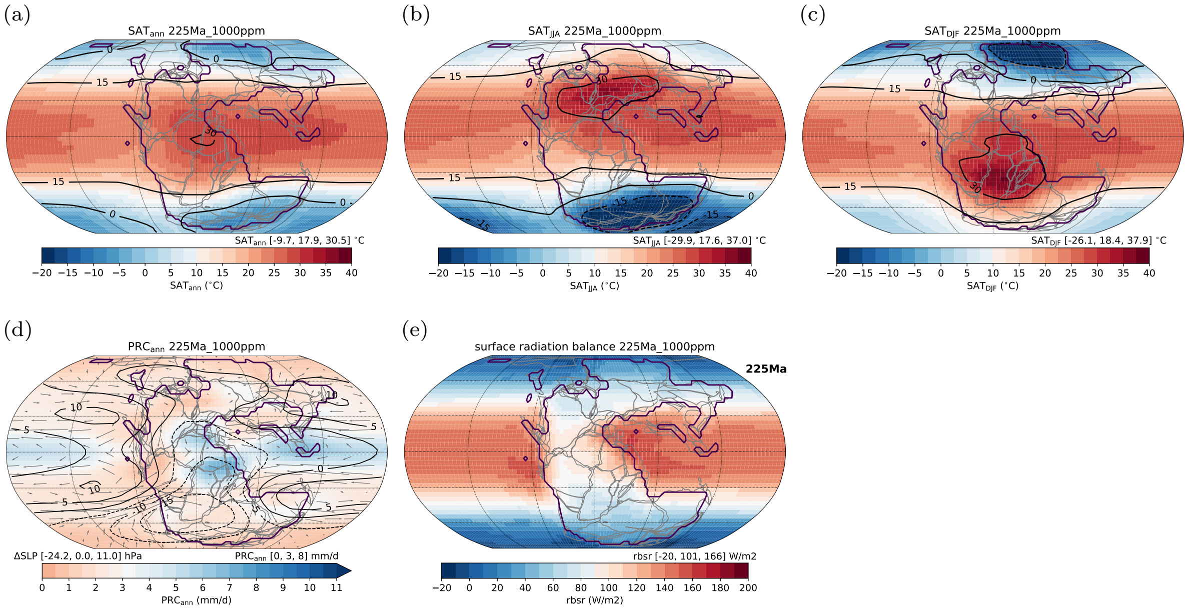


**Figure S10.** Same as Fig. S7 but at 225 Ma

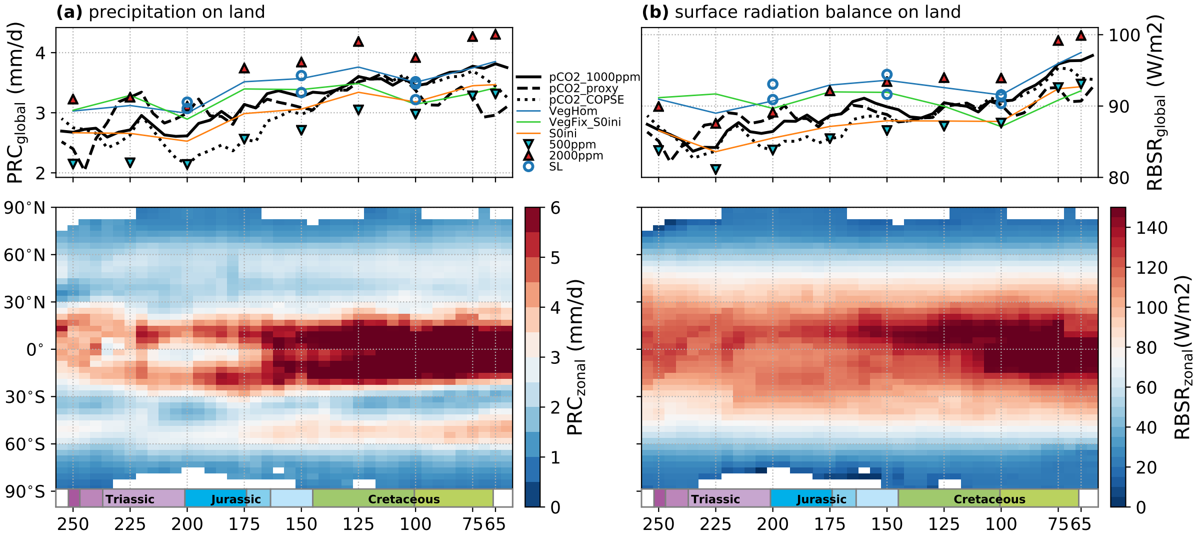


**Figure S11.** Zonal and global mean precipitation (a) and surface radiation balance (b) on land. Zonal mean values in the bottom panels are from the $P_{\mathtt{pCO2\_1000ppm}}$ baseline pathway, while the top panels also include results from the other pathways.

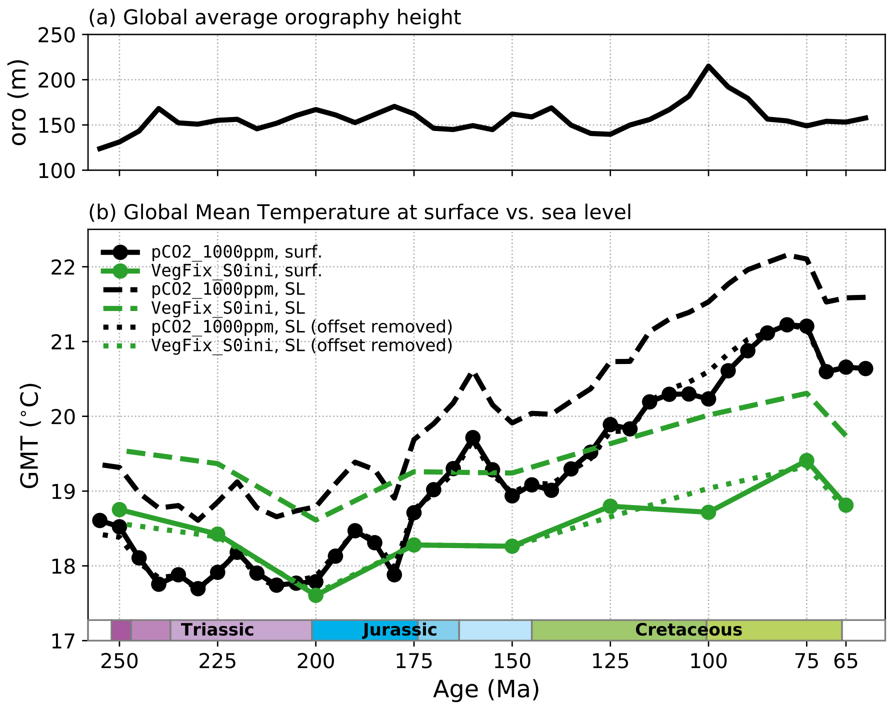


**Figure S12.** (a): Globally averaged height of the orography (Scotese & Wright, 2018) as implemented in the CLIMBER-3$\alpha$ model simulations. (b): Global mean surface air temperatures (GMST, solid lines) compared to lapse-rate corrected global mean temperatures at sea-level (dashed lines), for $P_{\texttt{pCO2\_1000ppm}}$ (black) and $P_{\texttt{VegFix\_S0ini}}$ (green). Dotted lines are also sea-level temperatures, but the offset to GMST at 150 Ma was removed to illustrate differences in the evolution through time.

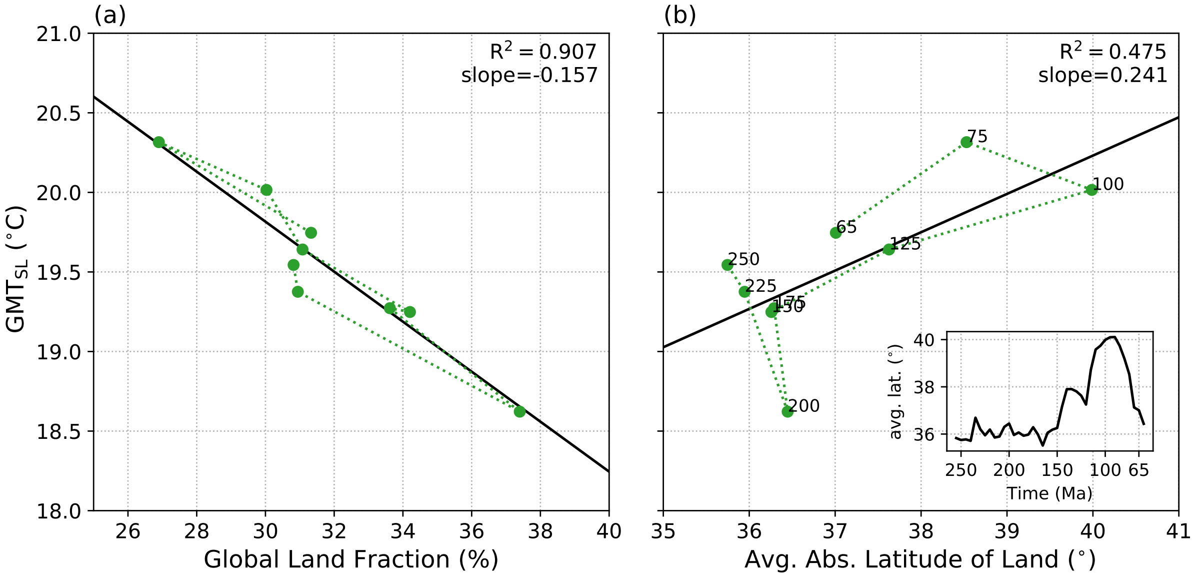


**Figure S13.** Correlation of lapse-rate corrected global mean air temperatures at sea level ($GMT_{SL}$) with (a) the global land area fraction and (b) the average absolute latitude of land area the time evolution of which is also shown in the inset. For the simulations of the $P_{\mathtt{VegFix\_SOini}}$ pathway.

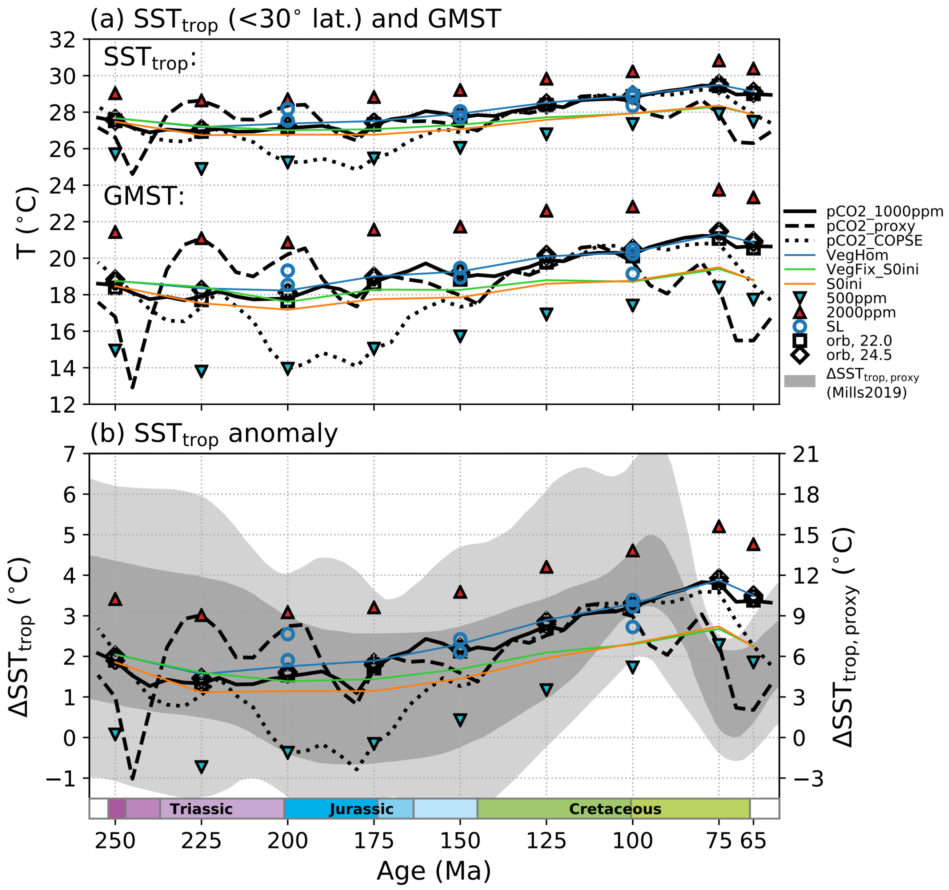


**Figure S14.** (a): Simulated global mean surface air temperatures and tropical sea surface temperatures (latitudes $< 30°$). (b): The gray shading indicates proxy-based tropical SST anomalies ($\Delta SST_{trop}$, relative to the present) from (Mills et al., 2019). For this the GMST envelope from Mills et al. (2019, Fig. 4a) was scaled back to ($\Delta SST_{trop}$) using Eq. 1 from the supplement to (Mills et al., 2019). Lines and markers indicate $\Delta SST_{trop}$ obtained for the CLIMBER-3$\alpha$ simulations, using the the scaling factor of 1.79 (as determined in Fig. S15). See Supp. Sec. 6 for further explanation.

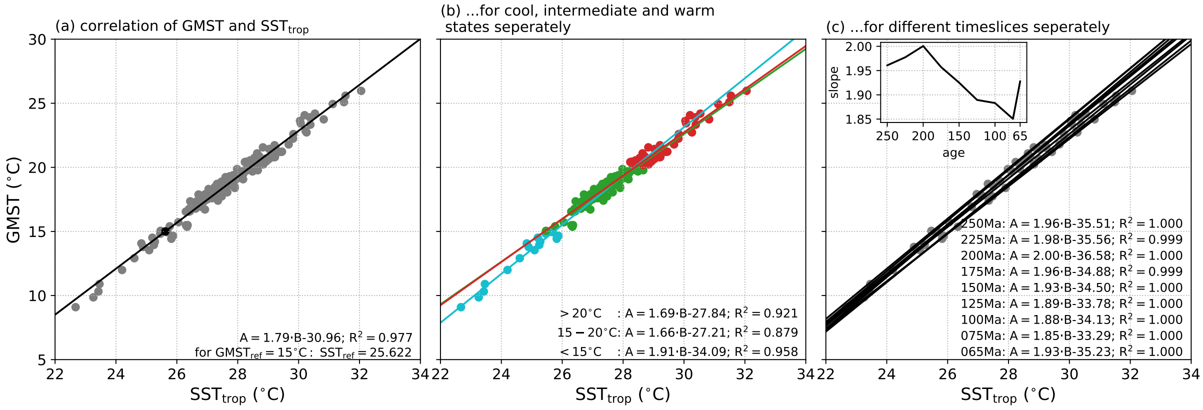


**Figure S15.** Correlation of simulated global mean surface air temperatures and tropical sea surface temperatures (latitudes $< 30°$). (a): For all simulations of the $\mathtt{P_{pCO2\_1000ppm}}$ pathway and all $pCO_2$ sensitivity experiments (each represented by one gray dot). (b): For cool (GMST $< 15\,°C$, cyan), intermediate ($15\,°C <$ GMST $< 20\,°C$, green) and warm (GMST $> 20\,°C$, red) states separately. (c): Separately for the $\mathtt{T_{25Myr}}$ timeslices including their climate states at $pCO_2 = [250, 500, 1000, 1500, 2000, 4000]$ ppm. The inset indicates the obtained scaling factor for these timeslices. See Supp. Sec. 6 for further explanation.

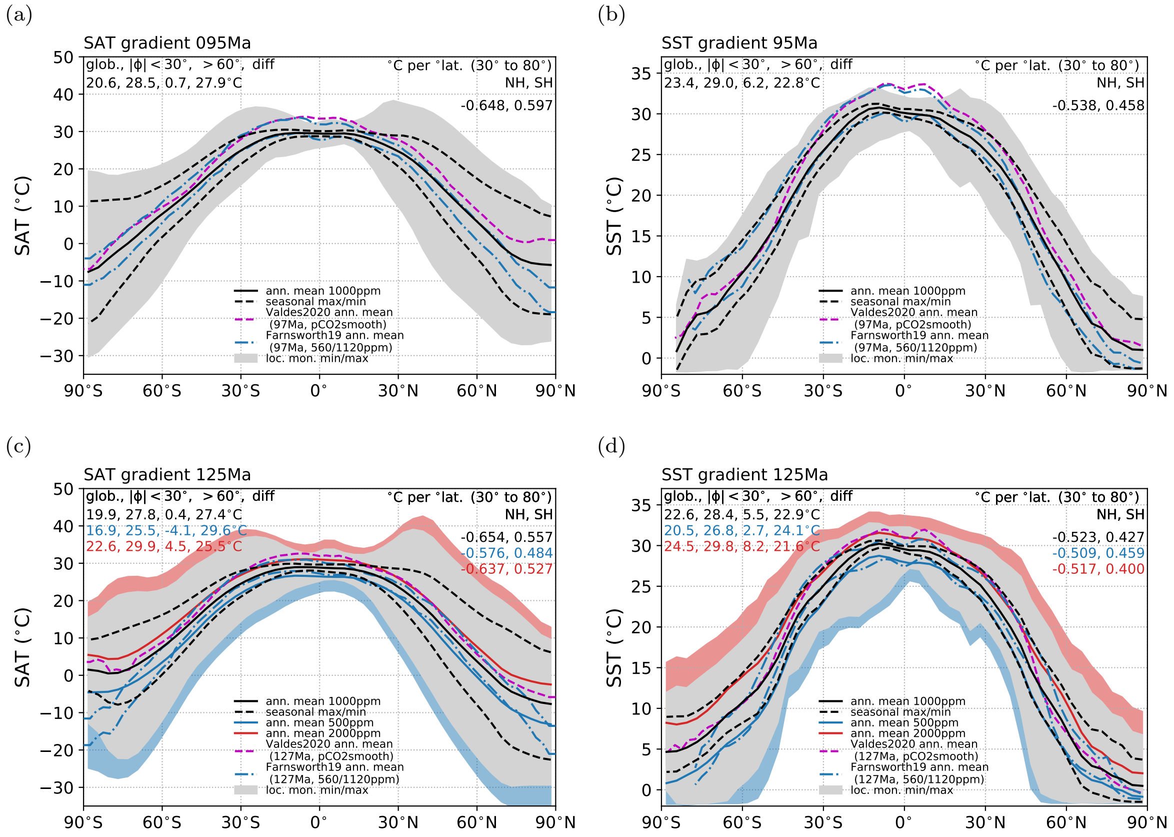


**Figure S16. Simulated zonal mean SAT (left) and SST (right) profiles for the 95 and 125 Ma timeslices.** For 1000ppm: annual mean (solid black line), seasonal min./max. (dashed black lines). For 500/1000/2000 ppm: monthly min./max. (blue/grey/red shading). Additionally annual mean for 500 ppm (solid blue line), 2000 ppm (solid red line), Valdes et al. (2020) data (dashed magenta line, `pCO2_smooth`), Farnsworth et al. (2019a) data (dashed blue line). Upper left corners: Numerical values of the global, low latitude (<30 °), high latitude (>60 °) averages and the difference between the latter two. Upper right corners: average meridional temperature gradient in °C per ° lat. between 30 and 80 ° latitude. Similar plots for all 40 Mesozoic timeslices are included in the accompanying data repository (AllTimeslices_MeridionalGradient_SST.pdf and AllTimeslices_MeridionalGradient_SST.pdf).

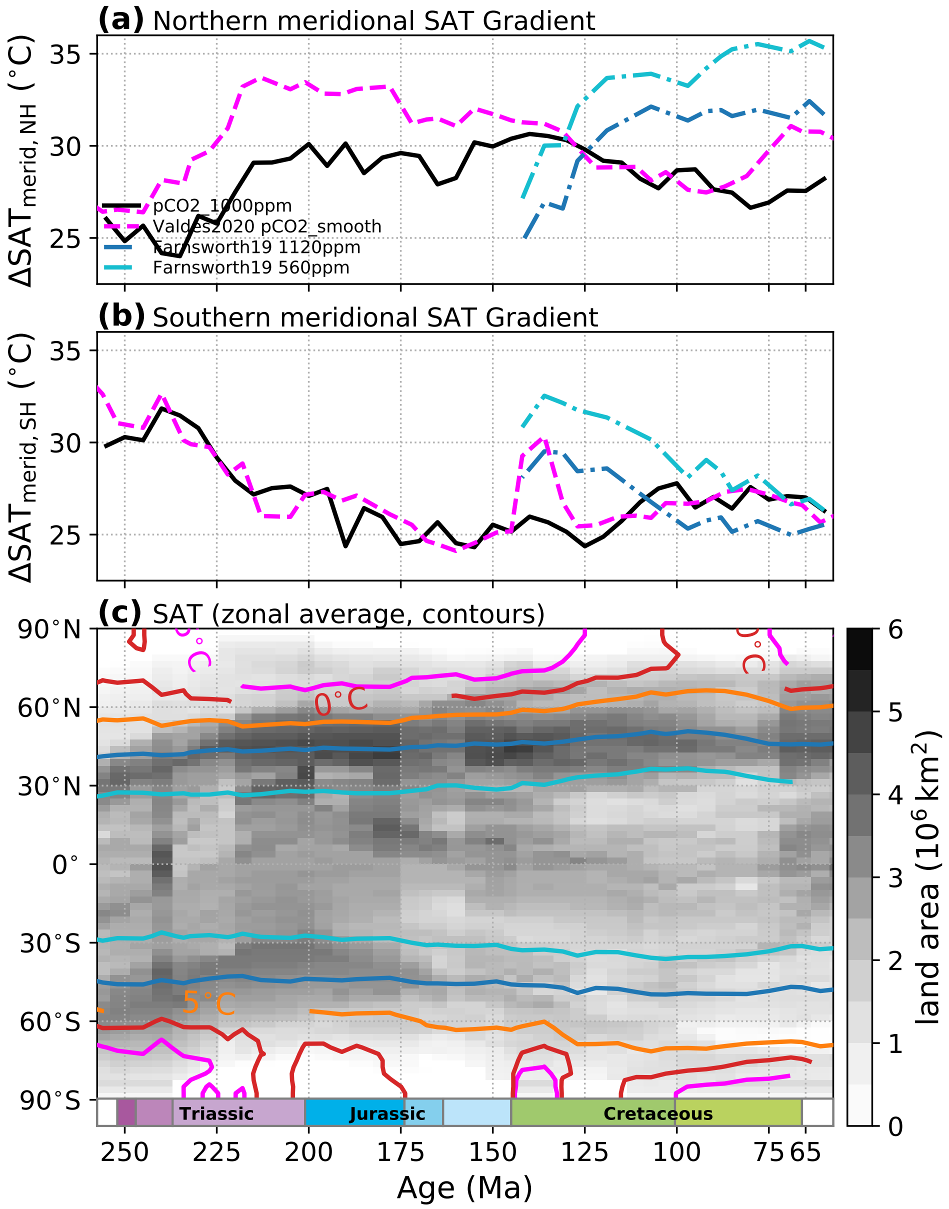


**Figure S17. Evolution of simulated zonal mean Surface Air Temperatures (SAT, c) and the thermal contrast between low-latitude and northern (a) and southern (c) high-latitude regions for data from Valdes et al. (2020) and Farnsworth et al. (2019a). (For comparison with Fig. 5.)** (c): Contours indicate annual zonal mean SAT for all runs of the `pCO2_smooth` pathway from Valdes et al. (2020). The grey shading indicates the changing latitudinal distribution of land area in the model paleogeographies. (a,b): Annual zonal mean SAT difference between low-latitude (<30° lat.) an northern (a) and southern (b) high-latitude regions (>60° lat.) through time.

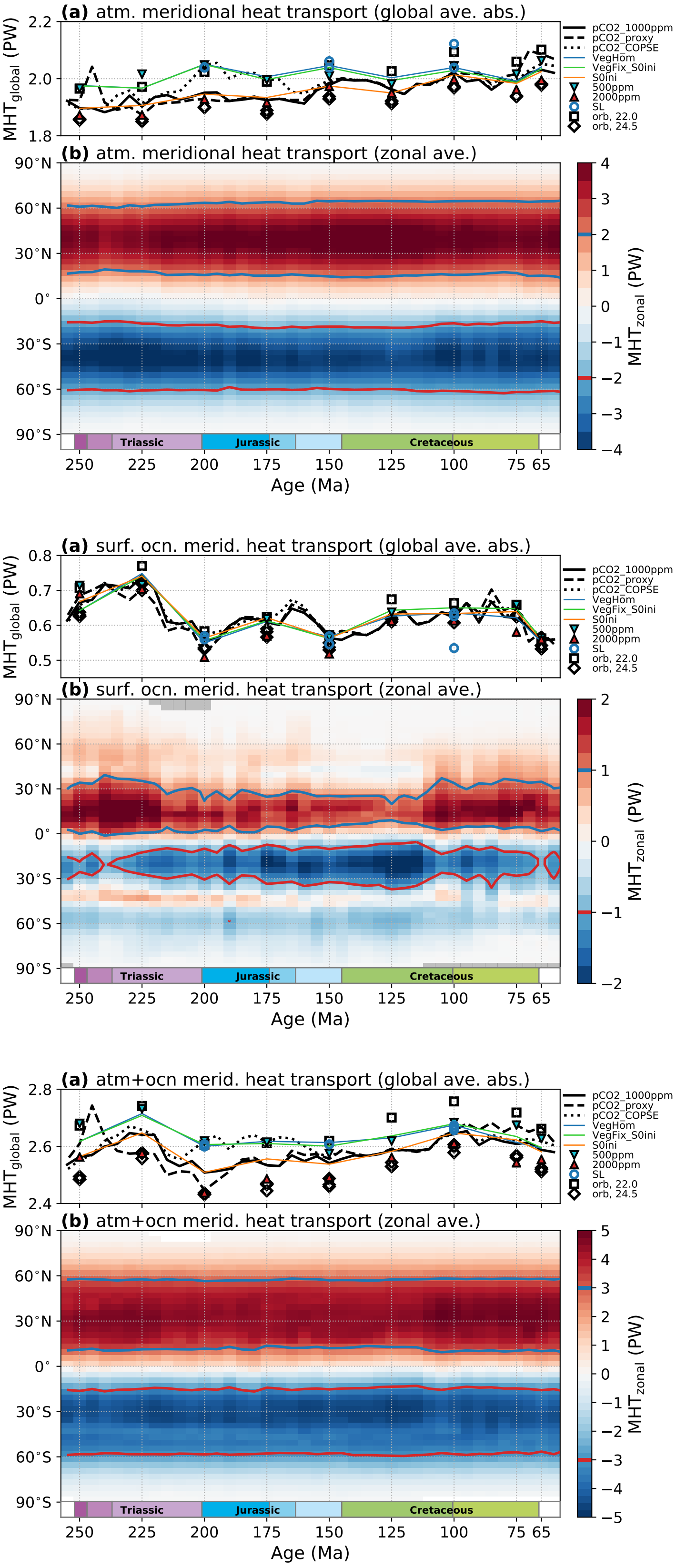


**Figure S18.** Meridional heat transport (MHT) by the atmosphere (top), ocean (middle) and in total (bottom). For the respective bottom panels, zonal mean values from all timeslices of the $P_{pCO2\_1000ppm}$ pathway have been aggregated. The upper panels indicate global mean values of the absolute MHT values.

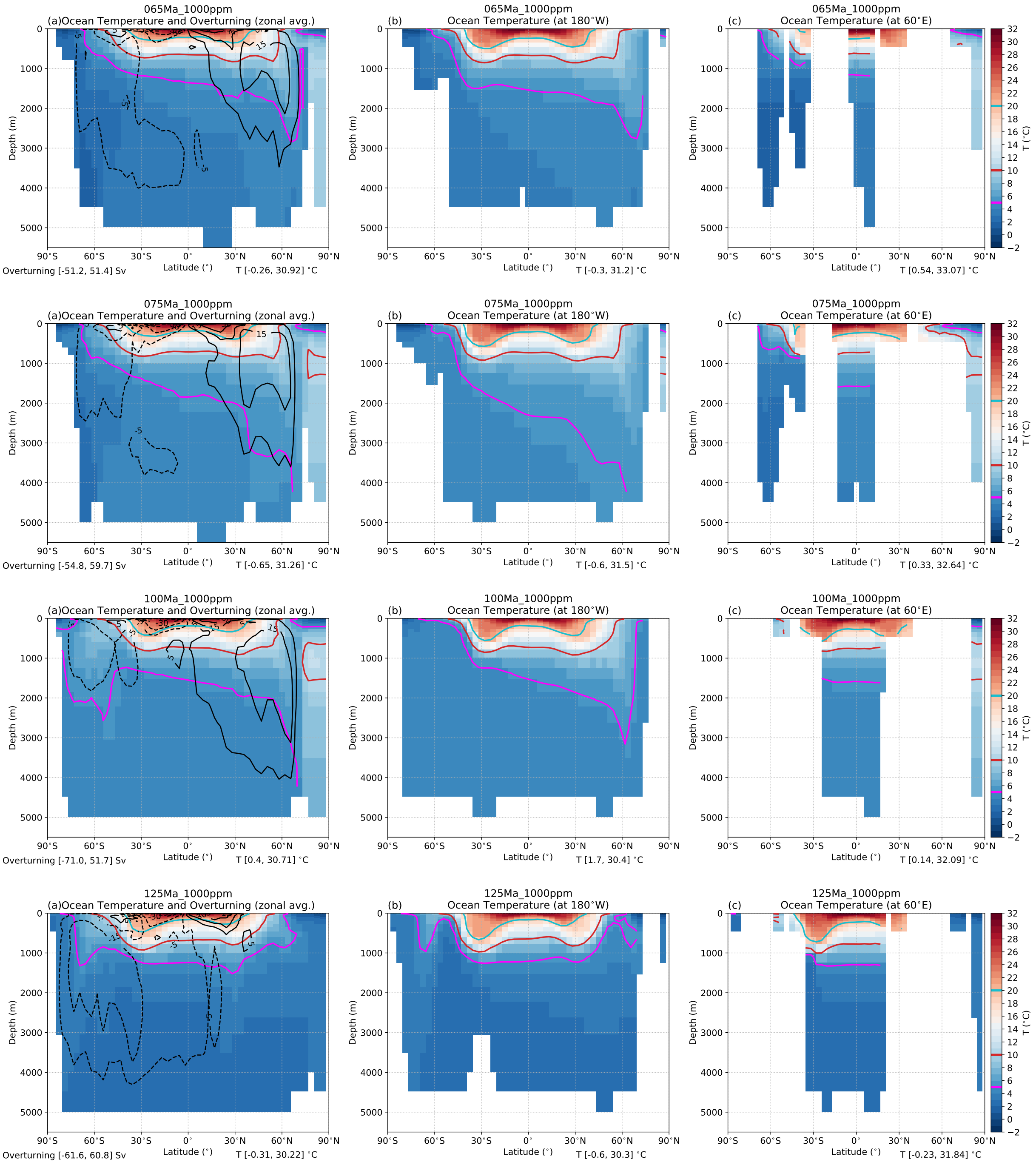


**Figure S19.** Meridional cross-sections showing the annual mean ocean temperatures as a zonal mean (a), at 180 °W (b) and 60 °E (c). In (a), the black contours indicate the global mean meriodional overturning streamfunction.

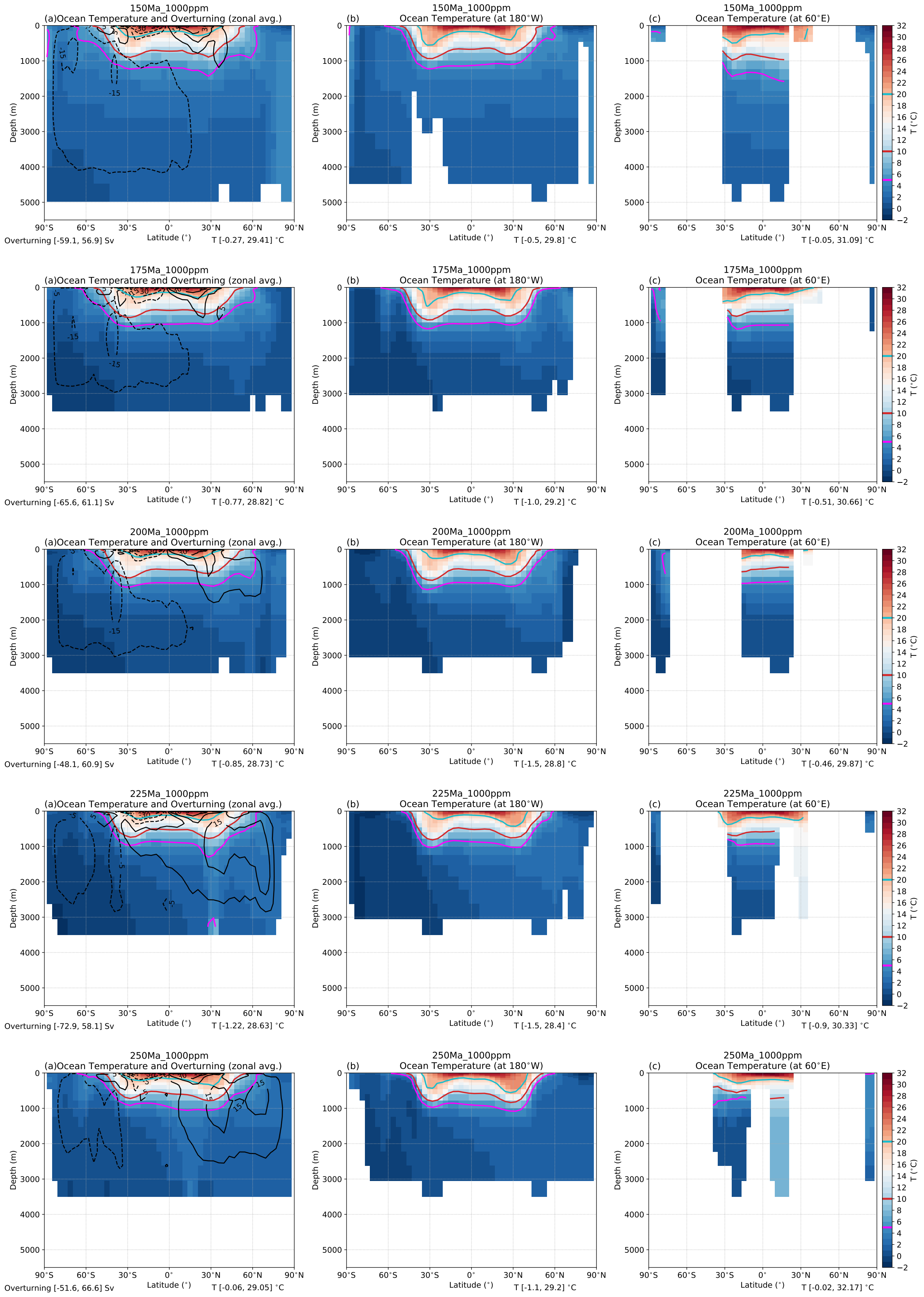


... Fig. S19 continued

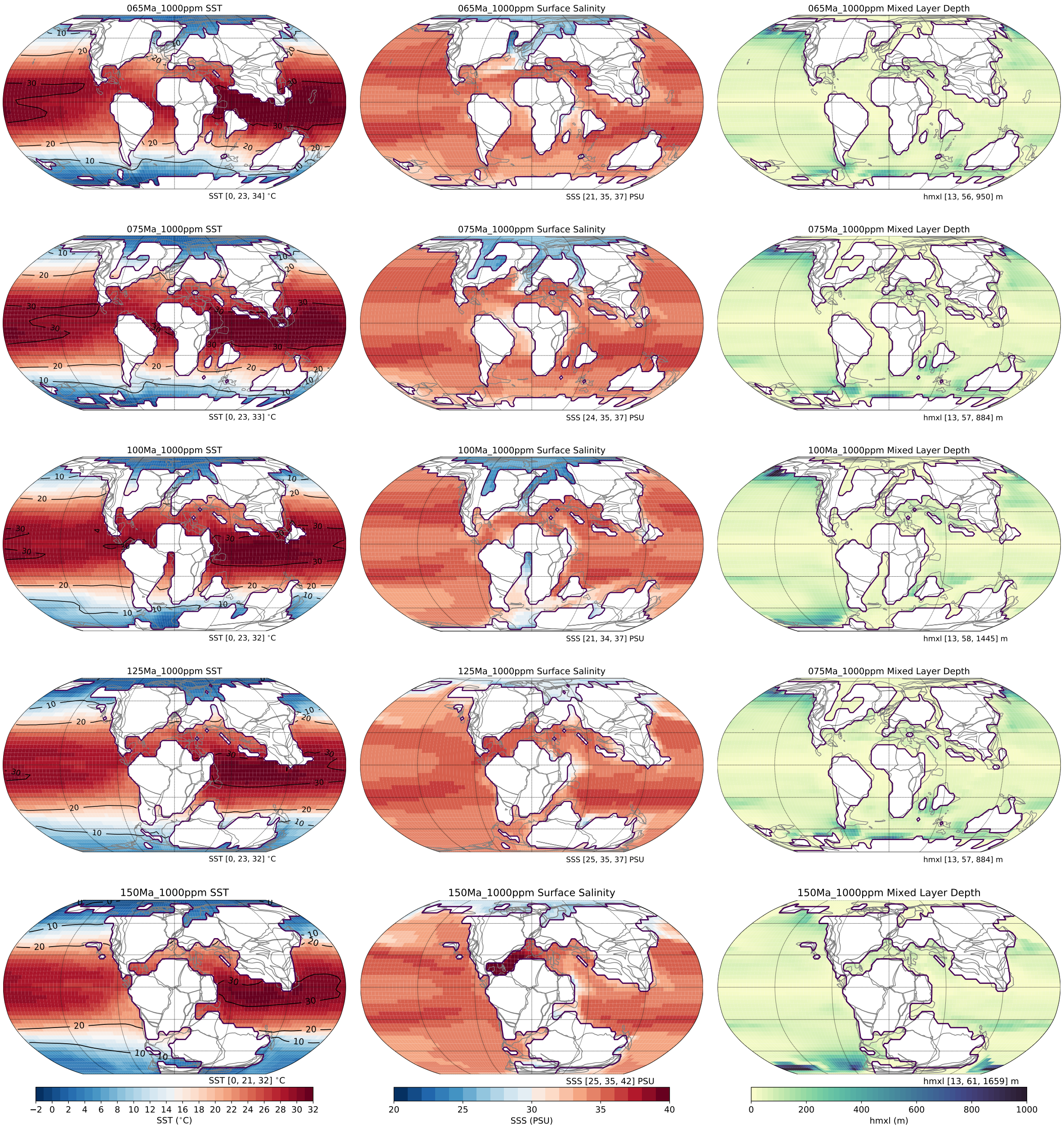


**Figure S20.** Annual mean Sea Surface Temperatures (left), Surface Salinity (middle) and Mixed Layer Depth (right). For the $P_{pCO2\_1000ppm}$ pathway.

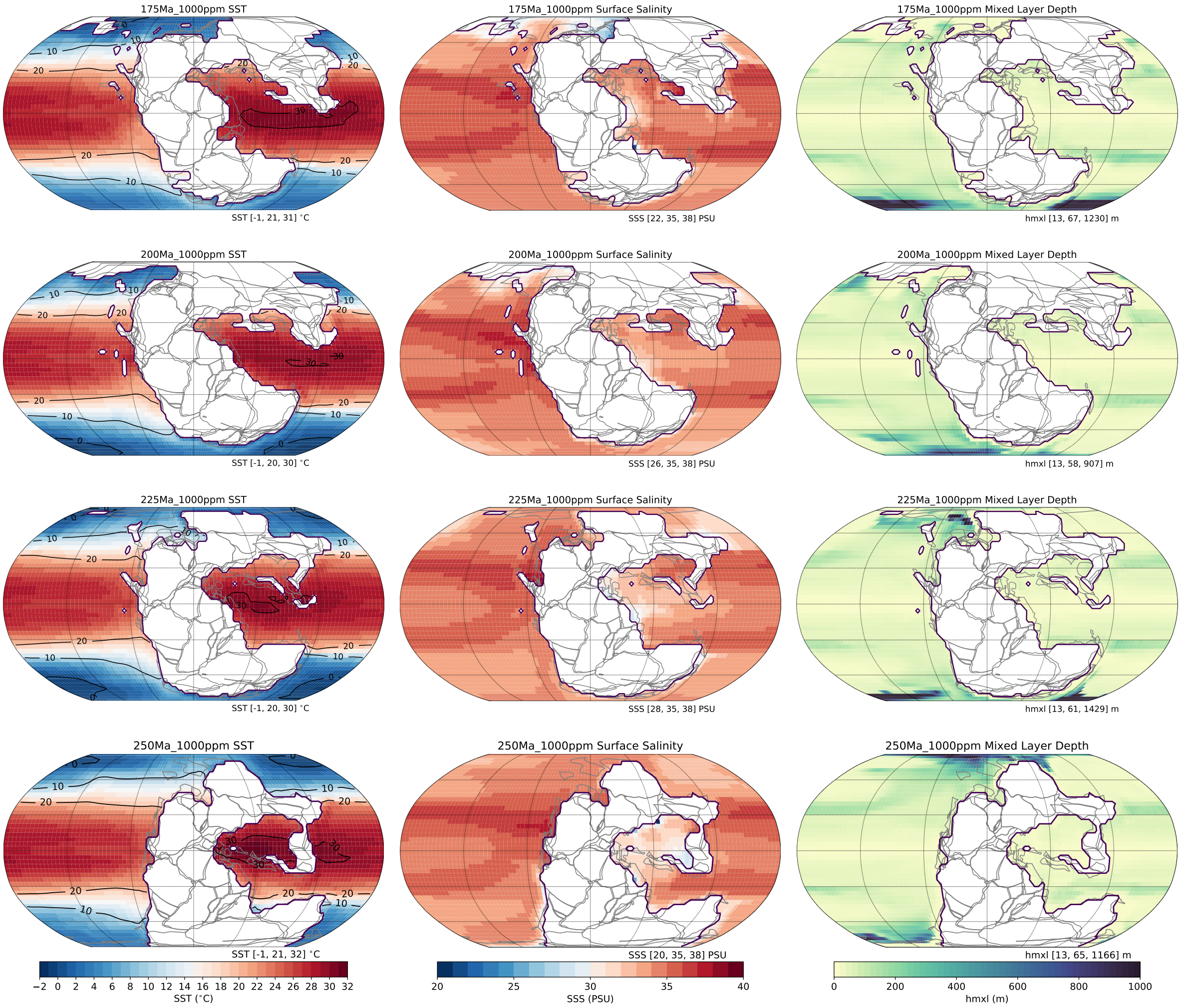


... Fig. S20 continued

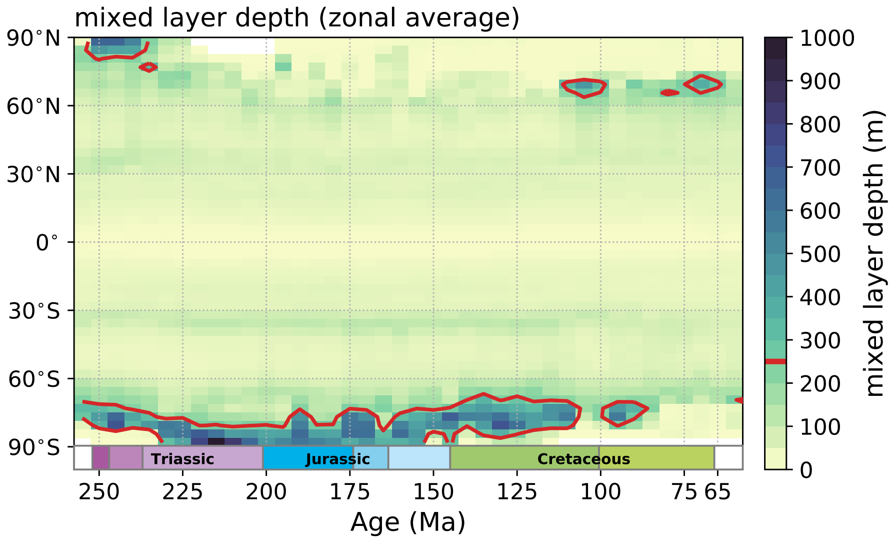


**Figure S21.** Zonal and annual mean mixed layer depth, aggregated for the simulations of the $P_{\mathtt{pCO2\_1000ppm}}$ pathway.

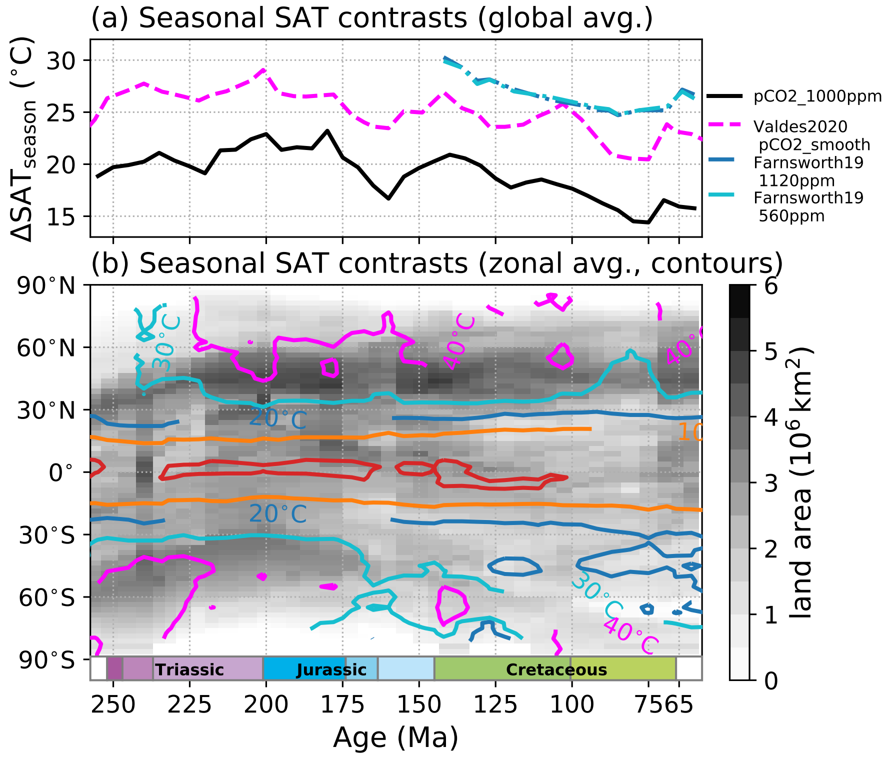


**Figure S22. Simulated seasonality of surface air temperatures (SAT).** With data from Valdes et al. (2020, $P_{\mathrm{pCO2\_smooth}}$ pathway) and Farnsworth et al. (2019a) for comparison with the results from CLIMBER-3α presented in Fig. 3. (b): Zonal averages of the seasonal differences (max. difference between the DJF, MAM, JJA and SON seasonal averages) for all timeslices of the (Valdes et al., 2020) simulations (contours). The grey shading shows the changing latitudinal land area distribution implemented in the simulations. (a): Globally averaged continental SAT seasonality.
